# Indium-Catalyzed Direct Conversion of Lactones into Thiolactones Using a Disilathiane as a Sulfur Source

**DOI:** 10.3390/molecules23061339

**Published:** 2018-06-02

**Authors:** Yohei Ogiwara, Ken Takano, Shuhei Horikawa, Norio Sakai

**Affiliations:** Department of Pure and Applied Chemistry, Faculty of Science and Technology, Tokyo University of Science, 2641 Yamazaki, Noda, Chiba 278-8510, Japan; yoheiogiwara@rs.tus.ac.jp (Y.O.); 7218534@ed.tus.ac.jp (K.T.); 7216665@alumni.tus.ac.jp (S.H.)

**Keywords:** indium catalyst, disilathiane, lactones, thiolactones

## Abstract

An indium-catalyzed reaction of lactones and a disilathiane leading to thiolactones is described. The direct synthesis of thiolactones from lactones with an appropriate sulfur source is one of the most attractive approaches in organic and pharmaceutical chemistry. In this context, we found an indium-catalyzed direct conversion of lactones into thiolactones in the presence of elemental sulfur and a hydrosilane via formation of the disilathiane in situ. On the basis of the previous reaction, the application utilizing the disilathiane as a sulfur source was performed herein for the efficient synthesis of a variety of thiolactone derivatives from lactones by an indium catalyst.

## 1. Introduction

The introduction of a sulfur atom to organic molecules is a significant topic in synthetic chemistry because it potentially provides complex and important sulfur-containing compounds directly. Therefore, a search for an undiscovered sulfur source that could be applicable to organic sulfur chemistry is imperative, and extensive efforts have been devoted to the development of molecular transformations utilizing a novel sulfur source by many research groups thus far [1,2,3,4,5,6]. In this context, our group has demonstrated that the copper-catalyzed construction of diaryl sulfides from aryl iodides and hexamethyldisilathiane, (Me_3_Si)_2_S [7]. In the reaction, the disilathiane functioned as an effective S1 source of sulfides, and the results suggested to us that the strategy employing the disilathiane could be acceptable for any other sulfur-introduction reactions [8,9,10,11,12].

**Scheme 1:**
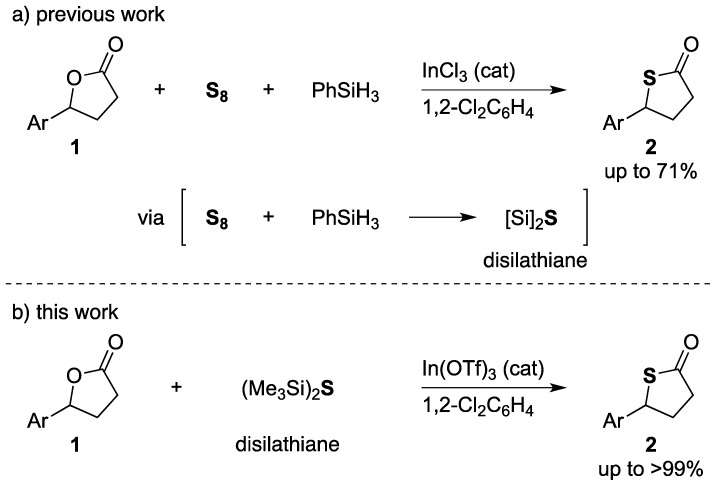
Indium-catalyzed conversion of lactones **1** to thiolactones **2** using (**a**) S_8_ and a hydrosilane, and (**b**) a disilathiane.

Recently, we also reported the indium-catalyzed reductive conversion of lactones **1** into thiolactones **2** using a combination of elemental sulfur (S_8_) and a hydrosilane, wherein the generation of a disilathiane ([Si]_2_S) from S_8_ and a hydrosilane is a key process for the formation of thiolactones **2** (Scheme 1a) [13]. Although the in situ formation strategy of the disilathiane is a useful and an easily handled procedure, the yields of thiolactones **2** obtained by the method remained at low to moderate levels. We envisioned that the problem could be overcome by utilizing the activated disilathiane, which can be easily prepared from S_8_ and a hydrosilane in advance. Herein, we describe the indium-catalyzed direct formation of thiolactones **2** from lactones **1** using hexamethyldisilathiane as an effective S1 source (Scheme 1b).

## 2. Results and Discussion

On the bases of our previous study on the InCl_3_-catalyzed transformation of lactones **1** to thiolactones **2** employing elemental sulfur (S_8_) and a hydrosilane, optimization studies utilizing a disilathiane as a sulfur source were initially conducted (Table 1). When γ-phenyl-γ-butyrolactone (**1a**) was treated with 1.1 equiv of hexamethyldisilathiane, (Me_3_Si)_2_S, in the presence of 5 mol % of InCl_3_ in 1,2-dichlorobenzene at 80 °C for 24 h, the corresponding γ-butyrothiolactone **2a** was obtained in a 77% GC yield (entry 1). The formation of **2a** was also observed in cases with other catalysts, such as InBr_3_, InI_3_, In(OAc)_3_, In(OTf)_3_, and Cu(OTf)_2_ in good yields (entries 2–6). Especially, In(OTf)_3_ proved to be the most effective catalyst for the reaction, shown in entry 5, which provided **2a** in a 99% GC yield with a 94% isolated yield. In contrast, in the absence of the catalyst, thiolactone was not generated (entry 7). We next chose chlorobenzene, 1,2-dichloroethane, and toluene as potential solvents for the transformation based on our previous results [13]. Although these solvents were also acceptable to the reaction, these yields were not higher than those using 1,2-Cl_2_C_6_H_4_ as a solvent (entries 8–10 vs. entry 5). The reaction with a lower catalyst loading (1 mol % of In(OTf)_3_) made it possible to form **2a** in a 97% GC yield with an 83% isolated yield (entry 11), and then a gram-scale application using **1a** (5 mmol) and (Me_3_Si)_2_S (5.5 mmol) provided 0.70 g (3.9 mmol) of thiolactone **2a** (entry 12). Employing a stoichiometric amount of TfOH instead of In(OTf)_3_ catalyst provided thiolactone quantitatively (entry 13), whereas its application to the TfOH-catalyzed reaction did not proceed well (entry 14).

**Figure 1:**
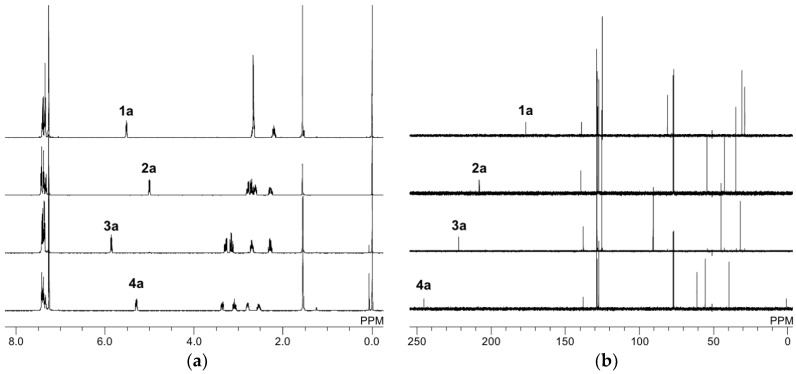
NMR spectra of **1a**–**4a**: (**a**) ^1^H-NMR (500 MHz, CDCl_3_, rt), (**b**) ^13^C-NMR (126 MHz, CDCl_3_, rt).

Conversion of lactone **1a** into thiolactone **2a**, and not into the other possible sulfur-containing compounds, such as the thionolactone **3a** and the dithiolactone **4a**, can be easily discriminated by ^1^H and ^13^C-NMR spectroscopy (Table 2 and Figure 1). In the ^1^H-NMR spectrum of the thiolactone **2a**, a signal for the methine proton (C*H*) next to the oxygen appeared at 5.01 ppm in CDCl_3_, which is clearly different to those of the corresponding protons of **1a** (5.52 ppm), **3a** (5.86 ppm), and **4a** (5.29 ppm). The chemical shifts of the carbonyl (*C*=O) or the thiocarbonyl (*C*=S) in the ^13^C-NMR spectrum can also identify the difference between **1a** (176.9 ppm in CDCl_3_), **2a** (207.9 ppm), **3a** (221.9 ppm), and **4a** (245.3 ppm). For most of the isolated products by this procedure shown below (e.g., Table 3), therefore, these structures were assigned as thiolactone **2** forms by ^1^H and ^13^C-NMR analyses (The detailed spectral data are summarized in Section 3.4. Product Characterization and the Supplementary Materials).

Examination of the In(OTf)_3_-catalyzed direct transformation of several lactones **1** was then conducted under the conditions described for entry 10 in Table 1. The results of the present reaction utilizing an In(OTf)_3_/(Me_3_Si)_2_S system (conditions A) and our previous yields of thiolactones **2** by an InCl_3_-catalyzed reaction using S_8_/PhSiH_3_ (conditions B) [13] are summarized in Table 3. In most of the substrates, the conditions A showed a better reactivity for the conversion of **1** into **2** than that of conditions B. Reactions of γ-aryl-γ-butyrolactons **1b**–**1k** bearing various functional groups at the aryl ring, such as methyl, phenyl, methoxy, and halogen, afforded the corresponding γ-aryl-γ-butyrothiolactones **2b**–**2k** (entries 1–10). Among these entries, the yield of a 4-methoxy-substituted one **2h** was not sufficient (entry 7), probably because of benzylic C–O bond cleavage prior to the reaction with disilathiane due to the strong electron donation by the 4-methoxy substituent. Similar results were also obtained in the case of our previous investigation employing an S_8_/PhSiH_3_ system prominently, which provided 4-(4-methoxyphenyl)butanoic acid as a side product in a 30% yield (entry 7, conditions B). A tetralin and a thiophene ring were also acceptable to γ-aryl substituents of the butyrolactons **1l** and **1m**, forming **2l** and **2m** (entries 11 and 12). The simple unsubstituted γ-butyrolactons **1n**, phthalide (**1o**), and its derivative **1p** were converted into the products **2n**–**2p**, respectively (entries 13–15). When δ-lactones **1q**–**1t** were used as starting substrates, the formation of the expected six-membered thiolactones **2q**–**2t** were observed (entries 16–19).

**Scheme 2:**
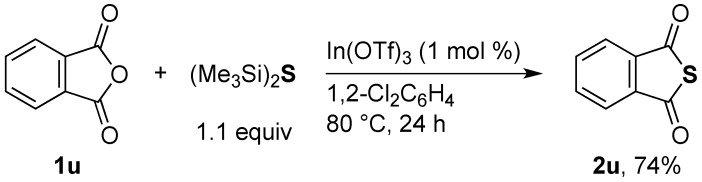
Reaction of phthalic anhydride.

Finally, the present procedure was evaluated using substrates involving an ester moiety, but not lactones. When phthalic anhydride (**1u**) was treated with a disilathiane, the corresponding reaction proceeded to give thiophthalic anhydride (**2u**) in a 74% isolated yield (Scheme 2). 

**Scheme 3:**
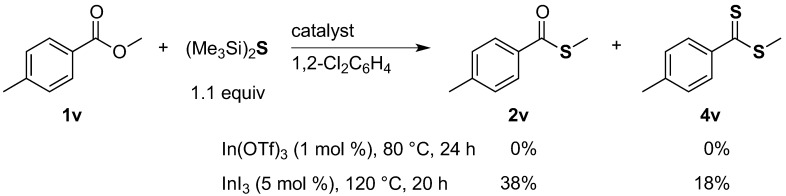
Reaction of an acyclic ester.

Although an acyclic ester, the methyl benzoate derivative **1v** was not acceptable to the transformation under the optimal conditions; the use of 5 mol % of InI_3_ catalyst at 120 °C for 20 h improved the reactivity for the reaction, leading to the expected thioester **2v** in a 38% isolated yield. Along with the formation of the thioester **2v** in those conditions, the unexpected dithioester **4v** was also isolated in an 18% yield (Scheme 3).

## 3. Materials and Methods

### 3.1. General Information

^1^H- and ^13^C-NMR spectra were recorded on a 300 or 500 MHz spectrometer. Chemical shifts in the ^1^H- and ^13^C-NMR spectra were reported in ppm relative to the residual solvent peaks, such as those of chloroform (δ 7.26 for ^1^H and δ 77.0 for ^13^C) or of the internal reference tetramethylsilane (δ 0.00 for both ^1^H and ^13^C). High-resolution mass spectra (HRMS) were measured using NBA (3-nitrobenzylalcohol) as a matrix. GC analyses were performed using a DB-5 capillary column (30 m × 0.25 mm, film thickness = 0.25 μm). Reactions were monitored by TLC analysis of the reaction aliquots. Column chromatography was performed using a silica gel. All indium compounds and hexamethyldisilathiane were commercially available and were used without further purification. 1,2-Dichlorobenzene was distilled from CaH_2_. The lactones **1a**, **1f**, **1n**, **1o**, **1q**, and **1t**, phthalic anhydride (**1u**), and an ester **1v** were purchased and used without further purification. The lactones **1b** and **1l** were prepared by the gallium-catalyzed reductive cyclization of keto acids [14]. The lactones **1c**, **1d**, **1e**, **1g**, **1h**, **1i**, **1j**, **1k**, **1m**, **1p**, **1r**, and **1s** were prepared via a modified literature method [15].

### 3.2. General Procedure A for the Indium-Catalyzed Conversion of Lactones or Their Derivatives ***1*** into Thiolactones ***2*** Using a Disilathiane (In the Case of ***1*** in Solid State at Room Temperature)

To a screw-capped tube, lactone or the derivative **1** (0.50 mmol) was added. The tube was sealed and moved into a glovebox, then In(OTf)_3_ (2.8 mg, 0.0050 mmol) was added. The tube was sealed again and removed from the glovebox. 1,2-Dichlorobenzene (0.5 mL) and hexamethyldisilathiane (98.1 mg, 0.550 mmol) were successively added, and after the tube was sealed, the mixture was heated at 80 °C for 24 h. The resulting mixture was cooled to room temperature and chloroform was added. The mixture was transferred into a round-bottom flask, which was then evaporated under reduced pressure. The crude material was purified by silica gel column chromatography (hexane/EtOAc) followed by gel permeation chromatography (GPC) in some cases.

### 3.3. General Procedure B for the Indium-Catalyzed Conversion of Lactones ***1*** into Thiolactones ***2*** Using a Disilathiane (In the Case of ***1*** in Liquid State at Room Temperature)

To a screw-capped tube, In(OTf)_3_ (2.8 mg, 0.0050 mmol) was added in a glovebox. The tube was then sealed and removed from the glovebox, and 1,2-dichlorobenzene (0.5 mL), lactone **1** (0.50 mmol), and hexamethyldisilathiane (98.1 mg, 0.550 mmol) were added in this order. After the tube was sealed, the mixture was heated at 80 °C for 24 h. The resulting mixture was cooled to room temperature and chloroform was added. The mixture was transferred into a round-bottom flask, which was then evaporated under reduced pressure. The crude material was purified by silica gel column chromatography (hexane/EtOAc) followed by gel permeation chromatography (GPC) in some cases.

### 3.4. Product Characterization

*Dihydro-5-phenyl-2(3H)-thiophenone* (**2a**) [13]. General procedure A was followed with *5-phenyldihydrofuran-2-one* (**1a**, 80.2 mg). Column chromatography (10/1 hexane/EtOAc) afforded **2a** as a colorless oil (73.4 mg, 83%): ^1^H-NMR (CDCl_3_, 500 MHz) δ 2.24–2.32 (m, 1 H, C*H*_2_), 2.59–2.82 (m, 3 H, C*H*_2_, C*H*_2_), 5.01 (dd, *J* = 10.0, 5.5 Hz, 1 H, C*H*), 7.30–7.44 (m, 5 H, Ar*H*); ^13^C-NMR (CDCl_3_, 126 MHz) δ 35.0, 42.9, 54.3, 127.4, 128.1, 128.9, 139.4, 207.9; MS (EI) *m*/*z* (%) 178 (M^+^, 78), 117 (100).

*Dihydro-5-(2-methylphenyl)-2(3H)-thiophenone* (**2b**) [13]. General procedure B was followed with *5-(2-methylphenyl)dihydrofuran-2-one* (**1b**, 89.9 mg). Column chromatography (10/1 hexane/EtOAc) afforded **2b** as a colorless oil (69.7 mg, 73%): ^1^H-NMR (CDCl_3_, 500 MHz) δ 2.26–2.34 (m, 1 H, C*H*_2_), 2.42 (s, 3 H, C*H*_3_), 2.55–2.61 (m, 1 H, C*H*_2_), 2.67–2.74 (m, 1 H, C*H*_2_), 2.78–2.84 (m, 1 H, C*H*_2_), 5.25 (dd, *J* = 9.5, 5.5 Hz, 1 H, C*H*), 7.19–7.27 (m, 3 H, Ar*H*), 7.56 (d, *J* = 8.0 Hz, 1 H, Ar*H*); ^13^C-NMR (CDCl_3_, 126 MHz) δ 19.5, 33.4, 42.7, 50.3, 126.5, 126.7, 127.8, 130.7, 135.7, 137.2, 208.0; MS (EI) *m*/*z* (%) 192 (M^+^, 82), 117 (100).

*Dihydro-5-(3-methylphenyl)-2(3H)-thiophenone* (**2c**) [13]. General procedure B was followed with *5-(3-methylphenyl)dihydrofuran-2-one* (**1c**, 92.3 mg). Column chromatography (10/1 hexane/EtOAc) afforded **2c** as a dark yellow oil (81.9 mg, 82%): ^1^H-NMR (CDCl_3_, 500 MHz) δ 2.23–2.31 (m, 1 H, C*H*_2_), 2.37 (s, 3 H, C*H*_3_), 2.57–2.81 (m, 3 H, C*H*_2_, C*H*_2_), 4.97 (dd, *J* = 10.0, 5.5 Hz, 1 H, C*H*), 7.13 (d, *J* = 7.5 Hz, 1 H, Ar*H*), 7.21–7.28 (m, 3 H, Ar*H*); ^13^C-NMR (CDCl_3_, 126 MHz) δ 21.4, 35.0, 42.9, 54.3, 124.4, 128.1, 128.7, 128.9, 138.6, 139.3, 208.0; MS (EI) *m*/*z* (%) 192 (M^+^, 100).

*Dihydro-5-(4-methylphenyl)-2(3H)-thiophenone* (**2d**) [13]. General procedure A was followed with *5-(4-methylphenyl)dihydrofuran-2-one* (**1d**, 87.5 mg). Column chromatography (10/1 hexane/EtOAc) afforded **2d** as a colorless oil (61.3 mg, 64%): ^1^H-NMR (CDCl_3_, 500 MHz) δ 2.21–2.29 (m, 1 H, C*H*_2_), 2.35 (s, 3 H, C*H*_3_), 2.56–2.78 (m, 3 H, C*H*_2_, C*H*_2_), 4.96 (dd, *J* = 10.0, 5.5 Hz, 1 H, C*H*), 7.17 (d, *J* = 8.0 Hz, 2 H, Ar*H*), 7.30 (d, *J* = 8.0 Hz, 2 H, Ar*H*); ^13^C-NMR (CDCl_3_, 126 MHz) δ 21.0, 35.0, 42.9, 54.1, 127.2, 129.5, 136.4, 137.9, 208.0; MS (EI) *m*/*z* (%) 192 (M^+^, 82), 117 (100).

*Dihydro-5-(2,5-dimethylphenyl)-2(3H)-thiophenone* (**2e**) [13]. General procedure A was followed with *5-(2,5-dimethylphenyl)dihydrofuran-2-one* (**1e**, 94.5 mg). Column chromatography (10/1 hexane/EtOAc) afforded **2e** as a yellow oil (66.6 mg, 65%): ^1^H-NMR (CDCl_3_, 500 MHz) δ 2.24–2.31 (m, 1 H, C*H*_2_), 2.33 (s, 3 H, C*H*_3_), 2.36 (s, 3 H, C*H*_3_), 2.53–2.58 (m, 1 H, C*H*_2_), 2.65–2.73 (m, 1 H, C*H*_2_), 2.76–2.82 (m, 1 H, C*H*_2_), 5.22 (dd, *J* = 10.0, 5.5 Hz, 1 H, C*H*), 7.01 (d, *J* = 7.5 Hz, 1 H, Ar*H*), 7.07 (d, *J* = 7.5 Hz, 1 H, Ar*H*), 7.36 (s, 1 H, Ar*H*); ^13^C-NMR (CDCl_3_, 126 MHz) δ 19.0, 21.0, 33.4, 42.7, 50.3, 127.1, 128.5, 130.6, 132.5, 136.2, 136.9, 208.1; MS (EI) *m*/*z* (%) 206 (M^+^, 85), 131 (100).

*Dihydro-5-([1,1′-biphenyl]-4-yl)-2(3H)-thiophenone* (**2f**). General procedure A was followed with *5-[1,1′-Biphenyl]-4-yldihydro-2(3H)-furanone* (**1f**, 96.2 mg). Column chromatography (10/1 hexane/EtOAc) and GPC afforded **2f** as a colorless solid (61.6 mg, 61%): m.p. 120–121 °C; ^1^H-NMR (CDCl_3_, 500 MHz) δ 2.78–2.36 (m, 1 H, C*H*_2_), 2.63–2.83 (m, 3 H, C*H*_2_, C*H*_2_), 5.05 (dd, *J* = 10.0, 5.5 Hz, 1 H, C*H*), 7.35–7.61 (m, 9 H, Ar*H*); ^13^C-NMR (CDCl_3_, 126 MHz) δ 35.0, 42.9, 54.1, 127.0, 127.5, 127.6, 127.9, 128.8, 138.4, 140.4, 141.1, 207.8; MS (EI) *m*/*z* (%) 254 (M^+^, 100); HRMS (EI) calcd for [M]^+^ (C_16_H_14_OS) *m*/*z* 254.0765, found 254.0771.

*Dihydro-5-(3-methoxyphenyl)-2(3H)-thiophenone* (**2g**) [13]. General procedure B was followed with *5-(3-methoxyphenyl)dihydrofuran-2-one* (**1g**, 100.9 mg). Column chromatography (10/1 hexane/EtOAc) afforded **2g** as a yellow oil (86.4 mg, 79%): ^1^H-NMR (CDCl_3_, 500 MHz) δ 2.21–2.30 (m, 1 H, C*H*_2_), 2.59–2.78 (m, 3 H, C*H*_2_, C*H*_2_), 3.81 (s, 3 H, C*H*_3_), 4.96 (dd, *J* = 10.0, 5.5 Hz, 1 H, C*H*), 6.85 (dd, *J* = 8.0, 3.0 Hz, 1 H, Ar*H*), 6.97 (s, 1 H, Ar*H*), 7.00 (d, *J* = 8.0 Hz, 1 H, Ar*H*), 7.28 (dd, *J* = 8.0, 8.0 Hz, 1 H, Ar*H*); ^13^C-NMR (CDCl_3_, 126 MHz) δ 34.8, 42.7, 54.2, 55.2, 113.1, 113.3, 119.6, 129.8, 140.9, 159.8, 207.8; MS (EI) *m*/*z* (%) 208 (M^+^, 100).

*Dihydro-5-(4-methoxyphenyl)-2(3H)-thiophenone* (**2h**) [13]. General procedure B was followed with *5-(4-methoxyphenyl)dihydrofuran-2-one* (**1h**, 97.7 mg). Column chromatography (10/1 hexane/EtOAc) afforded **2h** as a colorless solid (23.2 mg, 22%): m.p. 74–75 °C; ^1^H-NMR (CDCl_3_, 500 MHz) δ 2.21–2.29 (m, 1 H, C*H*_2_), 2.56–2.59 (m, 1 H, C*H*_2_), 2.65–2.80 (m, 2 H, C*H*_2_, C*H*_2_), 3.81 (s, 3 H, C*H*_3_), 4.97 (dd, *J* = 10.5, 5.5 Hz, 1 H, C*H*), 6.90 (d, *J* = 8.0 Hz, 2 H, Ar*H*), 7.35 (d, *J* = 8.0 Hz, 2 H, Ar*H*); ^13^C-NMR (CDCl_3_, 126 MHz) δ 35.2, 43.1, 54.0, 55.3, 114.2, 128.6, 131.3, 159.4, 208.1; MS (EI) *m*/*z* (%) 208 (M^+^, 79), 147 (100).

*Dihydro-5-(4-fluorophenyl)-2(3H)-thiophenone* (**2i**) [13]. General procedure B was followed with *5-(4-fluorophenyl)dihydrofuran-2-one* (**1i**, 96.3 mg). Column chromatography (10/1 hexane/EtOAc) afforded **2i** as a pale green oil (82.3 mg, 79%): ^1^H-NMR (CDCl_3_, 297 MHz) δ 2.17–2.29 (m, 1 H, C*H*_2_), 2.56–2.82 (m, 3 H, C*H*_2_, C*H*_2_), 4.99 (dd, *J* = 9.8, 5.0 Hz, 1 H, C*H*), 7.03–7.08 (m, 2 H, Ar*H*), 7.38–7.42 (m, 2 H, Ar*H*); ^13^C-NMR (CDCl_3_, 75 MHz) δ 35.1, 42.8, 53.5, 115.7 (d, *J*_C–F_ = 20.9 Hz), 129.0 (d, *J*_C–F_ = 9.0 Hz), 135.2 (d, *J*_C–F_ = 3.7 Hz), 162.3 (d, *J*_C–F_ = 246.8 Hz), 207.4; MS (EI) *m*/*z* (%) 196 (M^+^, 72), 135 (100).

*Dihydro-5-(4-chlorophenyl)-2(3H)-thiophenone* (**2j**) [13]. General procedure A was followed with *5-(4-chlorophenyl)dihydrofuran-2-one* (**1j**, 98.1 mg). Column chromatography (10/1 hexane/EtOAc) afforded **2j** as a colorless solid (85.5 mg, 87%): m.p. 45–48 °C; ^1^H-NMR (CDCl_3_, 500 MHz) δ 2.18–2.26 (m, 1 H, C*H*_2_), 2.59–2.80 (m, 3 H, C*H*_2_, C*H*_2_), 4.97 (dd, *J* = 10.0, 5.5 Hz, 1 H, C*H*), 7.34–7.35 (m, 4 H, Ar*H*); ^13^C-NMR (CDCl_3_, 126 MHz) δ 34.9, 42.7, 53.5, 128.7, 129.0, 133.8, 138.0, 207.2; MS (EI) *m*/*z* (%) 214 (M^+^+2, 23), 212 (M^+^, 63), 117 (100).

*Dihydro-5-(4-bromophenyl)-2(3H)-thiophenone* (**2k**) [13]. General procedure A was followed with *5-(4-bromophenyl)dihydrofuran-2-one* (**1k**, 120.1 mg). Column chromatography (10/1 hexane/EtOAc) afforded **2k** as a colorless solid (96.1 mg, 74%): m.p. 58–60 °C; ^1^H-NMR (CDCl_3_, 500 MHz) δ 2.18–2.26 (m, 1 H, C*H*_2_), 2.58–2.80 (m, 3 H, C*H*_2_, C*H*_2_), 4.95 (dd, *J* = 10.0, 5.5 Hz, 1 H, C*H*), 7.30 (d, *J* = 7.0 Hz, 2 H, Ar*H*), 7.50 (d, *J* = 7.0 Hz, 2 H, Ar*H*); ^13^C-NMR (CDCl_3_, 126 MHz) δ 34.9, 42.7, 53.6, 122.0, 129.1, 132.0, 138.6, 207.2; MS (EI) *m*/*z* (%) 258 (M^+^ + 2, 59), 256 (M^+^, 58), 117 (100).

*Dihydro-5-(5,6,7,8-tetrahydronaphthalen-2-yl)-2(3H)-thiophenone* (**2l**) [13]. General procedure B was followed with *5-(5,6,7,8-Tetrahydronaphthalen-2-yl)dihydrofuran-2(3H)-one* (**1l**, 96.8 mg). Column chromatography (10/1 hexane/EtOAc) and GPC afforded **2l** as a colorless oil (37.4 mg, 36%): ^1^H-NMR (CDCl_3_, 500 MHz) δ 1.78–1.81 (m, 4 H, C*H*_2_), 2.23–2.31 (m, 1 H, C*H*_2_), 2.55–2.80 (m, 7 H, C*H*_2_, C*H*_2_), 4.94 (dd, *J* = 10.5, 5.5 Hz, 1 H, C*H*), 7.07 (d, *J* = 8.0 Hz 1 H, Ar*H*) 7.12–7.15 (m, 2 H, Ar*H*); ^13^C-NMR (CDCl_3_, 126 MHz) δ 23.0, 23.1, 29.1, 29.4, 35.0, 43.0, 54.3, 124.4, 128.1, 129.6, 136.4, 137.3, 137.7, 208.3; MS (EI) *m*/*z* (%) 232 (M^+^, 100).

*Dihydro-5-(thiophen-2-yl)-2(3H)-thiophenone* (**2m**). General procedure B was followed with *dihydro-5-(3-thienyl)-2(3H)-furanone* (**1m**, 89.0 mg). Column chromatography (10/1 hexane/EtOAc) afforded **2m** as a light green oil (22.3 mg, 23%): ^1^H-NMR (CDCl_3_, 500 MHz) δ 2.34–2.42 (m, 1 H, C*H*_2_), 2.65–2.85 (m, 3 H, C*H*_2_, C*H*_2_), 5.29 (dd, *J* = 7.5, 5.5 Hz, 1 H, C*H*), 6.97 (dd, *J* = 5.0, 3.5 Hz, 1 H, Ar*H*), 7.07 (d, *J* = 3.5 Hz, 1 H, Ar*H*), 7.27 (dd, *J* = 5.0, 3.5 Hz, 1 H, Ar*H*); ^13^C-NMR (CDCl_3_, 126 MHz) δ 35.6, 42.3, 49.1, 125.3, 125.6, 126.9, 143.7, 207.0; MS (EI) *m*/*z* (%) 184 (M^+^, 64), 123 (100); HRMS (EI) calcd for [M]^+^ (C_8_H_8_OS_2_) *m*/*z* 184.0017, found 184.0011.

*Thiophthalide* (**2o**) [16]. General procedure A was followed with phthalide (**1o**, 67.6 mg). Column chromatography (10/1 hexane/EtOAc) afforded **2o** as a colorless solid (26.8 mg, 18%): m.p. 68–70 °C; ^1^H-NMR (CDCl_3_, 500 MHz) δ 4.48 (s, 2 H, C*H*_2_), 7.48 (dd, *J* = 7.5, 7.5 Hz, 1 H, Ar*H*), 7.55 (d, *J* = 7.0 Hz, 1 H, Ar*H*), 7.63 (dd, *J* = 7.5, 7.5 Hz, 1 H, Ar*H*), 7.85 (d, *J* = 7.5 Hz, 1 H, Ar*H*); ^13^C-NMR (CDCl_3_, 126 MHz) δ 34.6, 123.9, 126.3, 128.0, 133.1, 135.8, 147.0, 198.0; MS (EI) *m*/*z* (%) 150 (M^+^, 89), 121 (100).

*3-Phenyl-benzo[c]thiophen-1(3H)-one* (**2p**) [13]. General procedure A was followed with *3-phenylisobenzofuran-1-one* (**1p**, 105.9 mg). Column chromatography (10/1 hexane/EtOAc) afforded **2p** as a pale yellow solid (7.5 mg, 85%): m.p. 87–88 °C; ^1^H-NMR (CDCl_3_, 500 MHz) δ 5.91 (s, 1 H, C*H*), 7.25–7.36 (m, 6 H, Ar*H*), 7.48 (dd, *J* = 7.5, 7.5 Hz, 1 H, Ar*H*), 7.56 (dd, *J* = 7.5, 7.5 Hz, 1 H, Ar*H*), 7.86 (d, *J* = 7.5 Hz, 1 H, Ar*H*); ^13^C-NMR (CDCl_3_, 126 MHz) δ 54.6, 123.6, 126.6, 128.29, 128.33, 128.4, 129.1, 133.6, 135.7, 138.8, 151.2, 197.2; MS (EI) *m*/*z* (%) 226 (M^+^, 100).

*Tetrahydro-6-phenyl-2H-benzothiopyran-2-one* (**2q**) [13]. General procedure A was followed with *tetrahydro-6-phenyl-2H-pyran-2-one* (**1q**, 89.3 mg). Colum chromatography (10/1 hexane/EtOAc) afforded **2q** as an orange oil (9.7 mg, 10%): ^1^H-NMR (CDCl_3_, 500 MHz) δ 1.94–2.10 (m, 2 H, C*H*_2_), 2.14–2.19 (m, 1 H, C*H*_2_), 2.37–2.41 (m, 1 H, C*H*_2_), 2.57–2.43 (m, 1 H, C*H*_2_), 2.59–2.78 (m, 1 H, C*H*_2_), 4.65 (dd, *J* = 11.0, 4.0 Hz, 1 H, C*H*), 7.29–7.32 (m, 1 H, Ar*H*), 7.35–7.39 (m, 4 H, Ar*H*); ^13^C-NMR (CDCl_3_, 126 MHz) δ 22.7, 32.4, 40.5, 50.5, 127.7, 128.1, 128.9, 140.4, 201.3; MS (EI) *m*/*z* (%) 192 (M^+^, 59), 104 (100).

*Tetrahydro-6-(4-methylphenyl)-2H-thiopyran-2-one* (**2r**). General procedure A was followed with *tetrahydro-6-(4-methylphenyl)-2*H*-Pyran-2-one* (**1r**, 92.3 mg). Column chromatography (10/1 hexane/EtOAc) and GPC afforded **2r** as a colorless oil (9.5 mg, 9%): ^1^H-NMR (CDCl_3_, 500 MHz) δ 1.93–2.07 (m, 2 H, C*H*_2_), 2.15–2.17 (m, 1 H, C*H*_2_), 2.35 (s, 3 H, C*H*_3_), 2.37–2.44 (m, 1 H, C*H*_2_), 2.72–2.76 (m, 1 H, C*H*_2_), 4.61 (dd, *J* = 11.0, 3.5 Hz, 1 H, C*H*), 7.17 (d, *J =* 7.5 Hz, 2 H, Ar*H*), 7.26 (d, *J* = 7.5 Hz, 2 H, Ar*H*); ^13^C-NMR (CDCl_3_, 126 MHz) δ 21.1, 22.7, 32.4, 40.5, 50.2, 127.5, 129.5, 137.4, 137.9, 201.6; MS (EI) *m*/*z* (%) 206 (M^+^, 62), 118 (100); HRMS (EI) calcd for [M]^+^ (C_12_H_14_OS) *m*/*z* 206.0765, found 206.0766.

*6-(4-Chlorophenyl)tetrahydro-2H-thiopyran-2-one* (**2s**). General procedure A was followed with *6-(4-chlorophenyl)tetrahydro-2H-pyran-2-one* (**1s**, 107.5 mg). Column chromatography (10/1 hexane/EtOAc) and GPC afforded **2s** as a colorless oil (4.8 mg, 4%): ^1^H-NMR (CDCl_3_, 500 MHz) δ 1.93–2.02 (m, 2 H, C*H*_2_), 2.04–2.19 (m, 1 H, C*H*_2_), 2.34–2.39 (m, 1 H, C*H*_2_), 2.56–2.63 (m, 1 H, C*H*_2_), 2.73–2.78 (m, 1 H, C*H*_2_), 4.62 (dd, *J* = 11.0, 4.5 Hz, 1 H, C*H*), 7.30–7.35 (m, 4 H, Ar*H*); ^13^C-NMR (CDCl_3_, 126 MHz) δ 22.6, 32.4, 40.5, 49.7, 129.0, 129.1, 133.9, 139.0, 200.8; MS (EI) *m*/*z* (%) 226 (M^+^+2, 20), 210 (M^+^, 23), 138 (100); HRMS (EI) calcd for [M]^+^ (C_11_H_11_OSCl) *m*/*z* 226.0219, found 226.0241.

*1,4-Dihydro-3H-2-benzothiopyran-3-one* (**2t**) [13]. General procedure A was followed with *1,4-dihydro-3H-2-benzopyran-3-one* (**1t**, 74.3 mg). Column chromatography (10/1 hexane/EtOAc) afforded **2t** as a pale yellow solid (40.9 mg, 50%): m.p. 90–93 °C; ^1^H-NMR (CDCl_3_, 500 MHz) δ 3.79 (s, 2 H, C*H*_2_), 4.22 (s, 2 H, C*H*_2_), 7.21–7.32 (m, 4 H, Ar*H*); ^13^C-NMR (CDCl_3_, 126 MHz) δ 34.2, 49.2, 126.6, 127.4, 128.0, 128.7, 133.7, 134.2, 202.9; MS (EI) *m*/*z* (%) 164 (M^+^, 14), 104 (100).

*Phthalic thioanhydride* (**2u**) [17]. General procedure A was followed with *phthalic anhydride* (**1u**, 77.3 mg). Column chromatography (10/1 hexane/EtOAc) afforded **2u** as a yellow solid (56.1 mg, 74%): m.p. 68–70 °C; ^1^H-NMR (CDCl_3_, 500 MHz) δ 7.81–7.83 (m, 2 H, Ar*H*), 7.97–7.99 (m, 2 H, Ar*H*); ^13^C-NMR (CDCl_3_, 126 MHz) δ 123.8, 135.0, 138.7, 189.8; MS (EI) *m*/*z* (%) 164 (M^+^, 100).

*S-Methyl 4-methylbenzothioate* (**2v**). General procedure A was followed with *methyl 4-methylbenzoate* (**1v**, 75.0 mg). Column chromatography (100/1 hexane/EtOAc) afforded **2v** as a red oil (31.8 mg, 38%): ^1^H-NMR (CDCl_3_, 500 MHz) δ 2.40 (s, 3 H, C*H*_3_), 2.46 (s, 3 H, C*H*_3_), 7.24 (d, *J* = 14.5 Hz, 2 H, Ar*H*), 7.87 (d, *J* = 14.5 Hz, 2 H, Ar*H*); ^13^C-NMR (CDCl_3_, 126 MHz) δ 11.6, 21.7, 127.2, 129.2, 134.5, 144.1, 192.1; MS (EI) *m*/*z* (%) 166 (M^+^, 5), 119 (100).

*Methyl 4-methylbenzodithioate* (**4v**) [18]. General procedure A was followed with *methyl 4-methylbenzoate* (**1v**, 75.0 mg). Column chromatography (100/1 hexane/EtOAc) afforded **4v** as an orange oil (15.9 mg, 18%): ^1^H-NMR (CDCl_3_, 500 MHz) δ 2.38 (s, 3 H, C*H*_3_), 2.77 (s, 3 H, C*H*_3_), 7.18 (d, *J* = 13.5 Hz, 2 H, Ar*H*), 7.94 (d, *J* = 13.5 Hz, 2 H, Ar*H*); ^13^C-NMR (CDCl_3_, 126 MHz) δ 20.5, 21.5, 126.8, 129.0, 142.6, 143.2, 228.8; MS (EI) *m*/*z* (%) 182 (M^+^, 22), 135(100). 

*5-[1,1′-Biphenyl]-4-yldihydro-2(3H)-furanone* (**1f**). A colorless solid: m.p. 100–102 °C; ^1^H-NMR (CDCl_3_, 500 MHz) δ 2.19–2.29 (m, 1 H, C*H*_2_), 2.66–2.72 (m, 3 H, C*H*_2_), 5.54–5.57 (m, 1 H, C*H*), 7.35–7.46 (m, 5 H, Ar*H*), 7.58–7.62 (m, 4 H, Ar*H*); ^13^C-NMR (CDCl_3_, 126 MHz) δ 29.0, 30.9, 81.0, 125.8, 127.1, 127.45, 127.53, 128.8, 138.3, 140.4, 141.4, 176.9; MS (EI) *m*/*z* (%) 238 (M^+^, 100); HRMS (EI) calcd for [M]^+^ (C_16_H_14_O_2_) *m*/*z* 238.0994, found 238.1002.

*Tetrahydro-6-(4-methylphenyl)-2H-pyran-2-one* (**1r**). A colorless solid: m.p. 81–83 °C; ^1^H-NMR (CDCl_3_, 500 MHz) δ 1.80–1.88 (m, 1 H, C*H*_2_), 1.92–1.98 (m, 2 H, C*H*_2_), 2.09–2.14 (m, 1 H, C*H*_2_), 2.34 (s, 3 H, C*H_3_*), 2.51–2.56 (m, 1 H, C*H*_2_), 2.57–2.71 (m, 1 H, C*H*_2_), 5.30 (dd, *J* = 10.5, 3.5 Hz, 1 H, C*H*), 7.17 (d, *J* = 7.5 Hz, 2 H, Ar*H*), 7.22 (d, *J* = 7.5 Hz, 2 H, Ar*H*); ^13^C-NMR (CDCl_3_, 126 MHz) δ 18.4, 21.0, 29.3, 30.3, 81.5, 125.5, 129.1, 136.6, 137.8, 171.4; MS (EI) *m*/*z* (%) 190 (M^+^, 42), 118 (100); HRMS (EI) calcd for [M]^+^ (C_12_H_14_O_2_) *m*/*z* 190.0994, found 190.0995.

*Tetrahydro-6-(4-chlorophenyl)-2H-pyran-2-one* (**1s**). An orange solid: m.p. 91–98 °C; ^1^H-NMR (CDCl_3_, 500 MHz) δ 1.77–1.85 (m, 1 H, C*H*_2_), 1.96–2.01 (m, 2 H, C*H*_2_), 2.12–2.16 (m, 1 H, C*H*_2_), 2.53–2.60 (m, 1 H, C*H*_2_), 2.67–2.73 (m, 1 H, C*H*_2_), 5.32 (dd, *J* = 10.5, 3.0 Hz, 1 H, C*H*), 7.28 (d, *J* = 8.5 Hz, 2 H, Ar*H*), 7.34 (d, *J* = 8.5 Hz, 2 H, Ar*H*); ^13^C-NMR (CDCl_3_, 126 MHz) δ 18.4, 29.3, 30.4, 80.8, 127.0, 128.6, 133.9, 138.2, 171.0; MS (EI) *m*/*z* (%) 212 (M^+^+2, 7), 210 (M^+^, 23), 70 (100); HRMS (EI) calcd for [M]^+^ (C_11_H_11_O_2_Cl) *m*/*z* 210.0448, found 210.0449.

## 4. Conclusions

An indium-catalyzed formation of thiolactones from lactones and a disilathiane was developed. A disilathiane was found to be a novel and an effective sulfur source for this type of conversion, and a wide range of lactone derivatives were successfully converted into the corresponding thiolactones.

## Figures and Tables

**Table 1:**
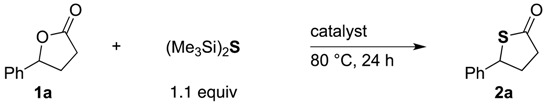
Screening of the reaction conditions for the catalytic conversion of **1a** to **2a** ^*a*^.

Entry	Catalyst	Solvent	GC Yield of 2a
1	InCl_3_ (5 mol %)	1,2-Cl_2_C_6_H_4_	77
2	InBr_3_ (5 mol %)	1,2-Cl_2_C_6_H_4_	62
3	InI_3_ (5 mol %)	1,2-Cl_2_C_6_H_4_	69
4	In(OAc)_3_ (5 mol %)	1,2-Cl_2_C_6_H_4_	78
5	In(OTf)_3_ (5 mol %)	1,2-Cl_2_C_6_H_4_	99 (94) *^b^*
6	Cu(OTf)_2_ (5 mol %)	1,2-Cl_2_C_6_H_4_	94
7	none	1,2-Cl_2_C_6_H_4_	0
8	In(OTf)_3_ (5 mol %)	ClC_6_H_5_	76
9	In(OTf)_3_ (5 mol %)	ClCH_2_CH_2_Cl	95
10	In(OTf)_3_ (5 mol %)	CH_3_C_6_H_5_	59
11	In(OTf)_3_ (1 mol %)	1,2-Cl_2_C_6_H_4_	97 (83) *^b^*
12	In(OTf)_3_ (1 mol %)	1,2-Cl_2_C_6_H_4_	78 ^*b,c*^
13	TfOH (100 mol %)	1,2-Cl_2_C_6_H_4_	99
14	TfOH (15 mol %)	1,2-Cl_2_C_6_H_4_	23

*^a^* Reaction conditions: **1a** (0.5 mmol), (Me_3_Si)_2_S (0.55 mmol), catalyst (0.005–0.025 mmol), solvent (0.5 mL) at 80 °C for 24 h. *^b^* Isolated yield. *^c^* 5 mmol scale.

**Table 2:** Selected ^1^H and ^13^C-NMR spectral data (CDCl_3_, rt) for **1a**, **2a**, **3a**, and **4a**.

	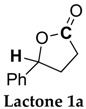	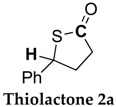	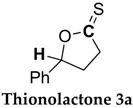	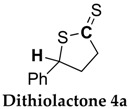
^1^H-NMR	*δ* 5.52 (OC**H**)	*δ* 5.01 (SC**H**)	*δ* 5.86 (OC**H**)	*δ* 5.29 (SC**H**)
^13^C-NMR	*δ* 176.9 (**C**=O)	*δ* 207.9 (**C**=O)	*δ* 221.9 (**C**=S)	*δ* 245.3 (**C**=S)

**Table 3:**
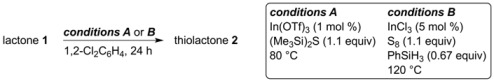
Indium-catalyzed conversion of lactones **1** to the thiolactones **2**.

**Entry**	**Lactone 1**	**Thiolactone 2**	**Isolated Yield of 2 (%)**	
			Conditions A *^a^*	Conditions B *^b^*
	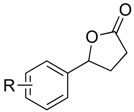		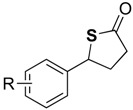	
1	1b (R = 2-Me)	2b	73	71
2	1c (R = 3-Me)	2c	82	55
3	1d (R = 4-Me)	2d	64	66
4	1e (R = 2,5-Me_2_)	2e	65	49
5	1f (R = 4-Ph)	2f	61	n/a *^c^*
6	1g (R = 3-MeO)	2g	79	16
7	1h (R = 4-MeO)	2h	22	8 *^d^*
8	1i (R = 4-F)	2i	79	61
9	1j (R = 4-Cl)	2j	87	60
10	1k (R = 4-Br)	2k	74	60
	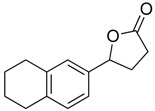		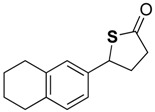	
11	1l	2l	36	34
	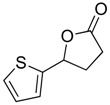		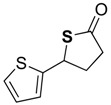	
12	1m	2m	23	0
	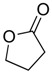		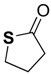	
13	1n	2n	66 *^e^*	n/a *^c^*
	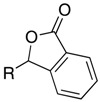		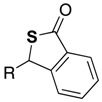	
14	1o (R = H)	2o	18 *^f^*	0
15	1p (R = Ph)	2p	85	10
	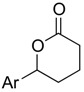		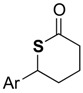	
16	1q (Ar = Ph)	2q	10 *^g^*	14
17	1r (Ar = 4-MeC_6_H_4_)	2r	9	n/a *^c^*
18	1s (Ar = 4-ClC_6_H_4_)	2s	4	n/a *^c^*
	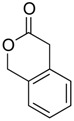		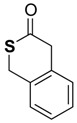	
19	1t	2t	88 *^h^*	23

*^a^* This work: **1** (0.5 mmol), (Me_3_Si)_2_S (0.55 mmol), In(OTf)_3_ (0.005 mmol), 1,2-Cl_2_C_6_H_4_ (0.5 mL) at 80 °C for 24 h. *^b^* Previous work: **1** (0.5 mmol), S_8_ (0.55 mmol of S atom), PhSiH_3_ (0.33 mmol), InCl_3_ (0.025 mmol), 1,2-Cl_2_C_6_H_4_ (0.5 mL) at 120 °C for 24 h. *^c^* Not applicable. *^d^* 4-(4-Methoxyphenyl)butanoic acid (30%) was formed. *^e^* NMR yield. *^f^* 7 d. *^g^* 120 °C, 3 days. *^h^* InCl_3_ (0.025 mmol, 5 mol %), 120 °C, 20 h.

## Supplementary Materials

The following are available online: ^1^H and ^13^C-NMR spectra of **2a**–**2m**; **2o**–**2v**; **4v**; **1f**; **1r** and **1s.**
