# Supplementary material for: Indium-Catalyzed Direct Conversion of Lactones into Thiolactones Using a Disilathiane as a Sulfur Source
Source: Molecules. 2018 Jun 2;23(6):1339. doi: 10.3390/molecules23061339 (PMC6100358; doi:10.3390/molecules23061339)
Supplement: Supplementary file 1 [file molecules-23-01339-s001.pdf]

Supplementary Materials

for

Indium-Catalyzed Direct Conversion of Lactones into Thiolactones Using a Disilathiane as a Sulfur Source

Yohei Ogiwara, Ken Takano, Shuhei Horikawa, and Norio Sakai*

Department of Pure and Applied Chemistry, Faculty of Science and Technology
Tokyo University of Science, 2641 Yamazaki, Noda, Chiba 278-8510, Japan

sakachem@rs.noda.tus.ac.jp

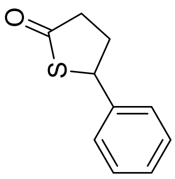

O
S

2a

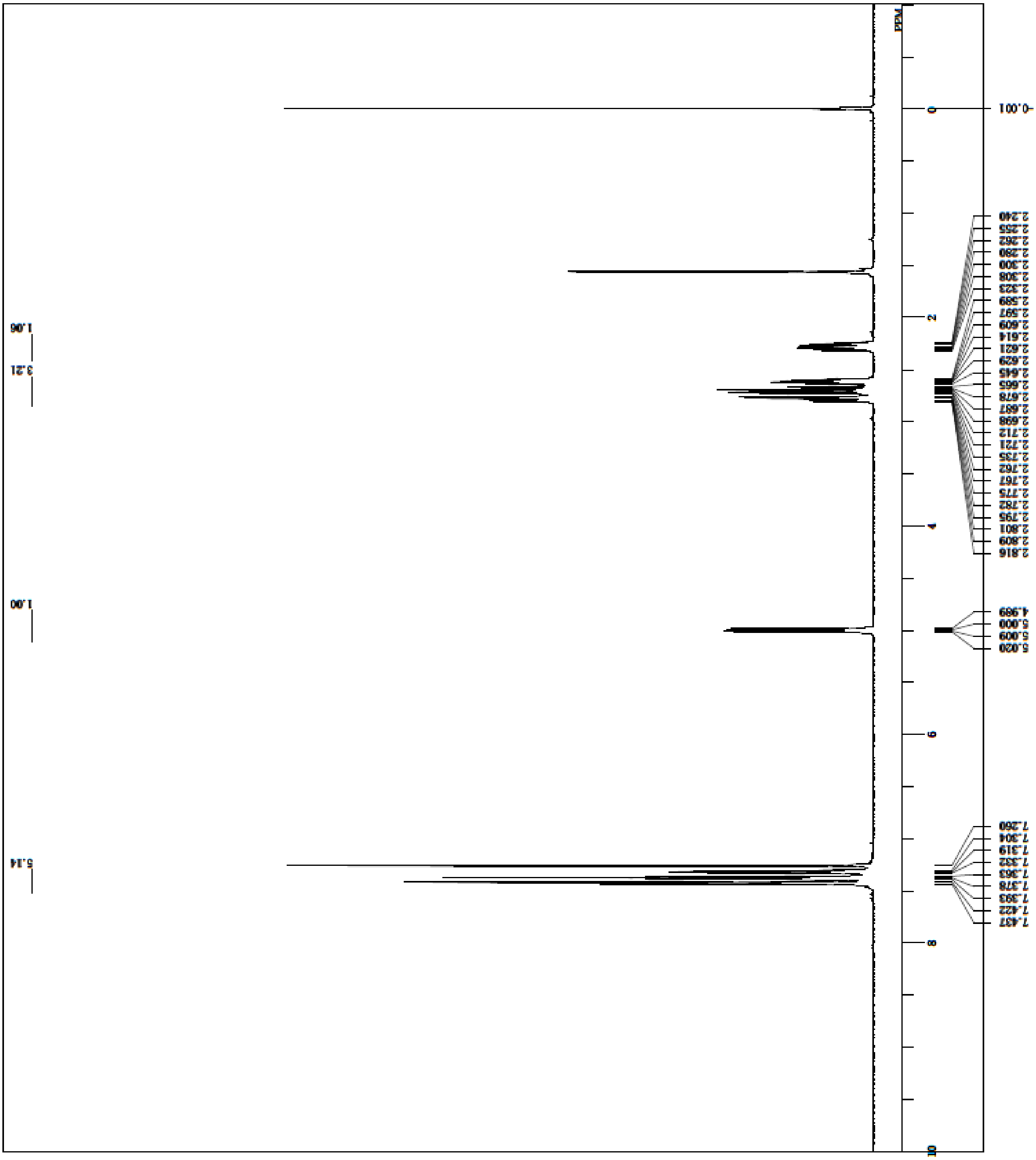

PPM
-0.001
2.240
2.255
2.262
2.280
2.300
2.308
2.323
2.589
2.597
2.609
2.609
2.614
2.621
2.629
2.645
2.665
2.678
2.687
2.698
2.712
2.721
2.735
2.762
2.767
2.775
2.782
2.795
2.801
2.809
2.816
4.999
5.000
5.009
5.020
7.280
7.304
7.319
7.332
7.363
7.378
7.393
7.422
7.437
1.06
3.21
1.00
5.14

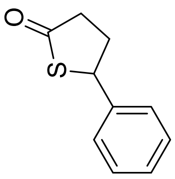

2a

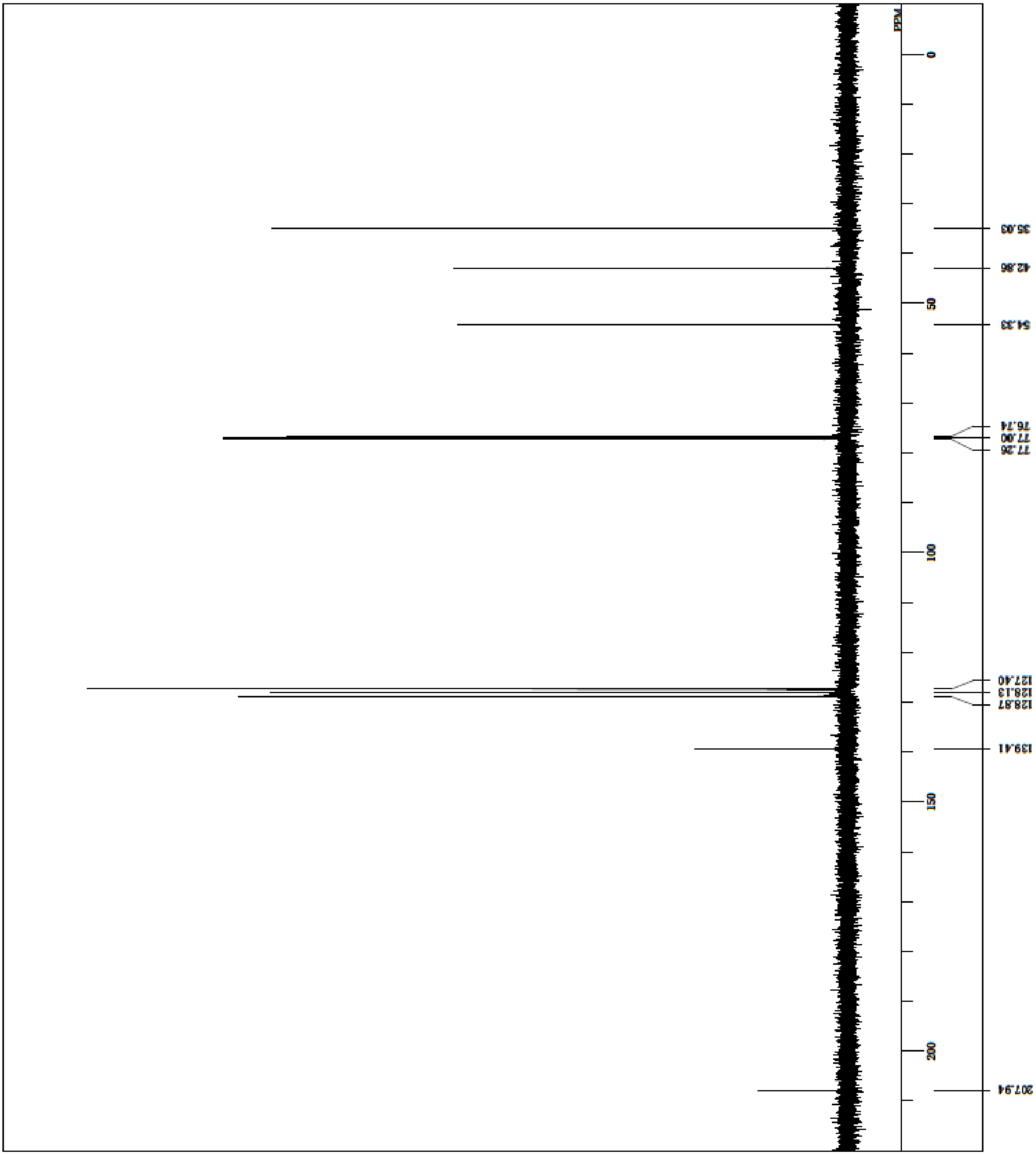
PPM
0
50
100
150
200
35.03
42.86
54.33
76.74
77.00
77.26
127.40
128.13
128.87
139.41
207.94

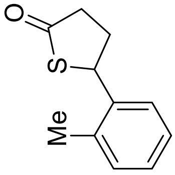
O
S
Me

2b

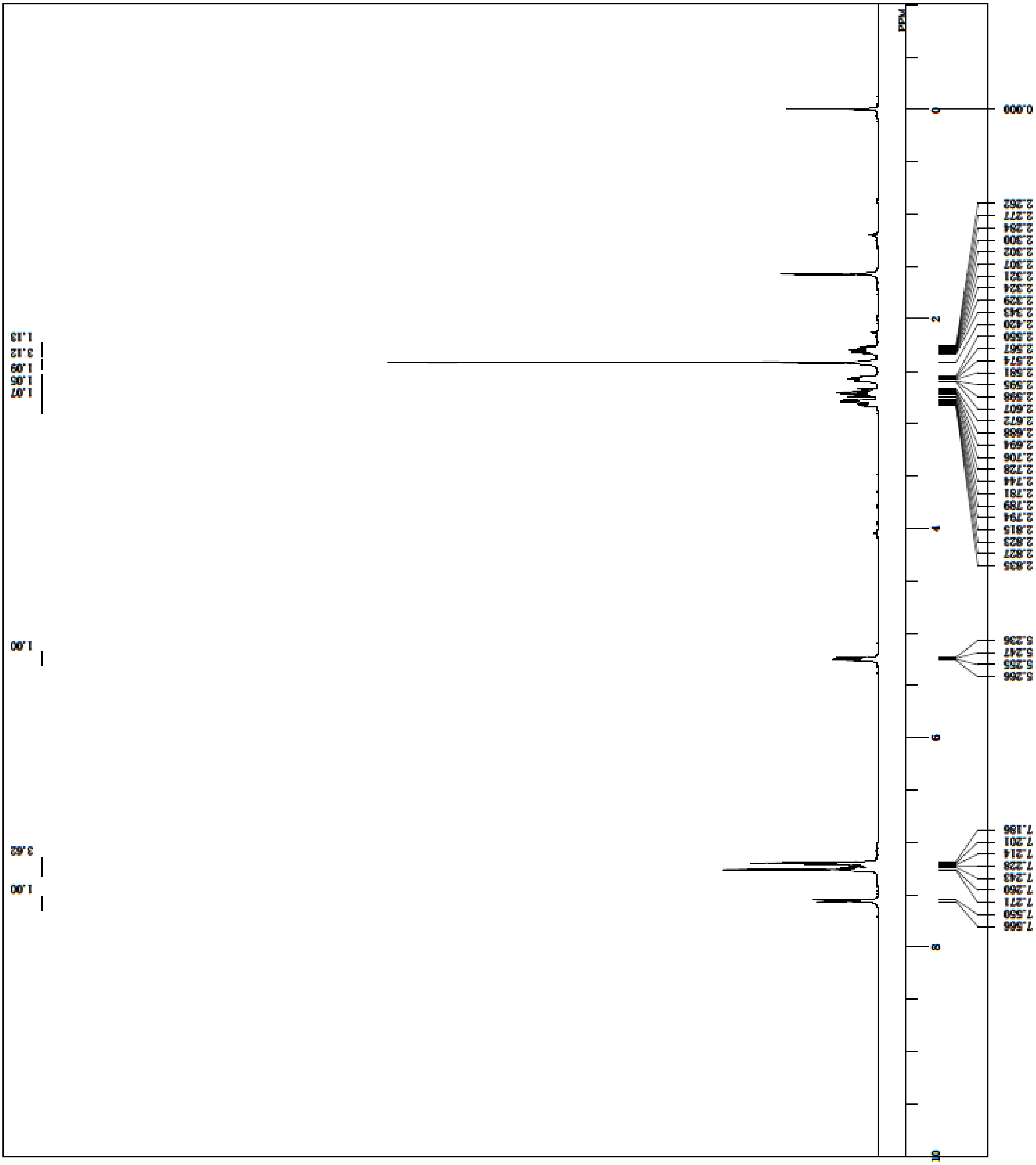
PPM
0
2
4
6
8
10
0.000
2.262
2.277
2.294
2.300
2.302
2.307
2.321
2.324
2.329
2.343
2.420
2.550
2.567
2.574
2.581
2.595
2.598
2.607
2.672
2.688
2.694
2.706
2.728
2.744
2.781
2.789
2.794
2.815
2.823
2.827
2.835
5.236
5.247
5.255
5.266
7.186
7.201
7.214
7.228
7.243
7.260
7.271
7.550
7.566
1.13
3.12
1.09
1.06
1.07
1.00
3.62
1.00

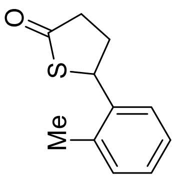
O
S
Me

2b

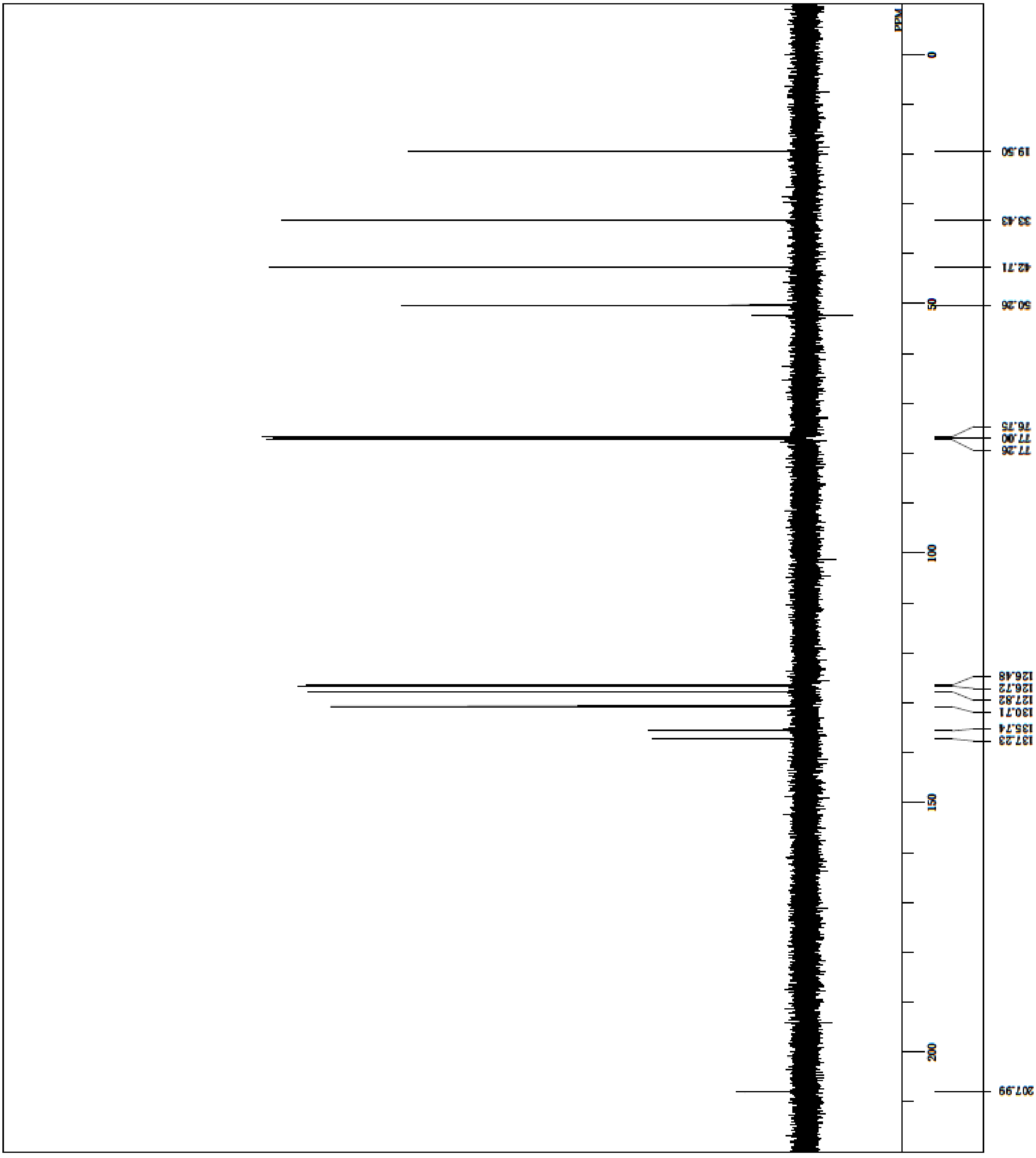
PPM
0
50
100
150
200
19.50
33.43
42.71
50.26
76.75
77.00
77.26
126.48
126.72
127.82
130.71
135.74
137.23
207.99

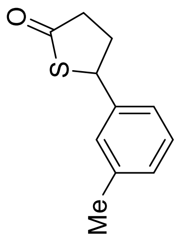

2c

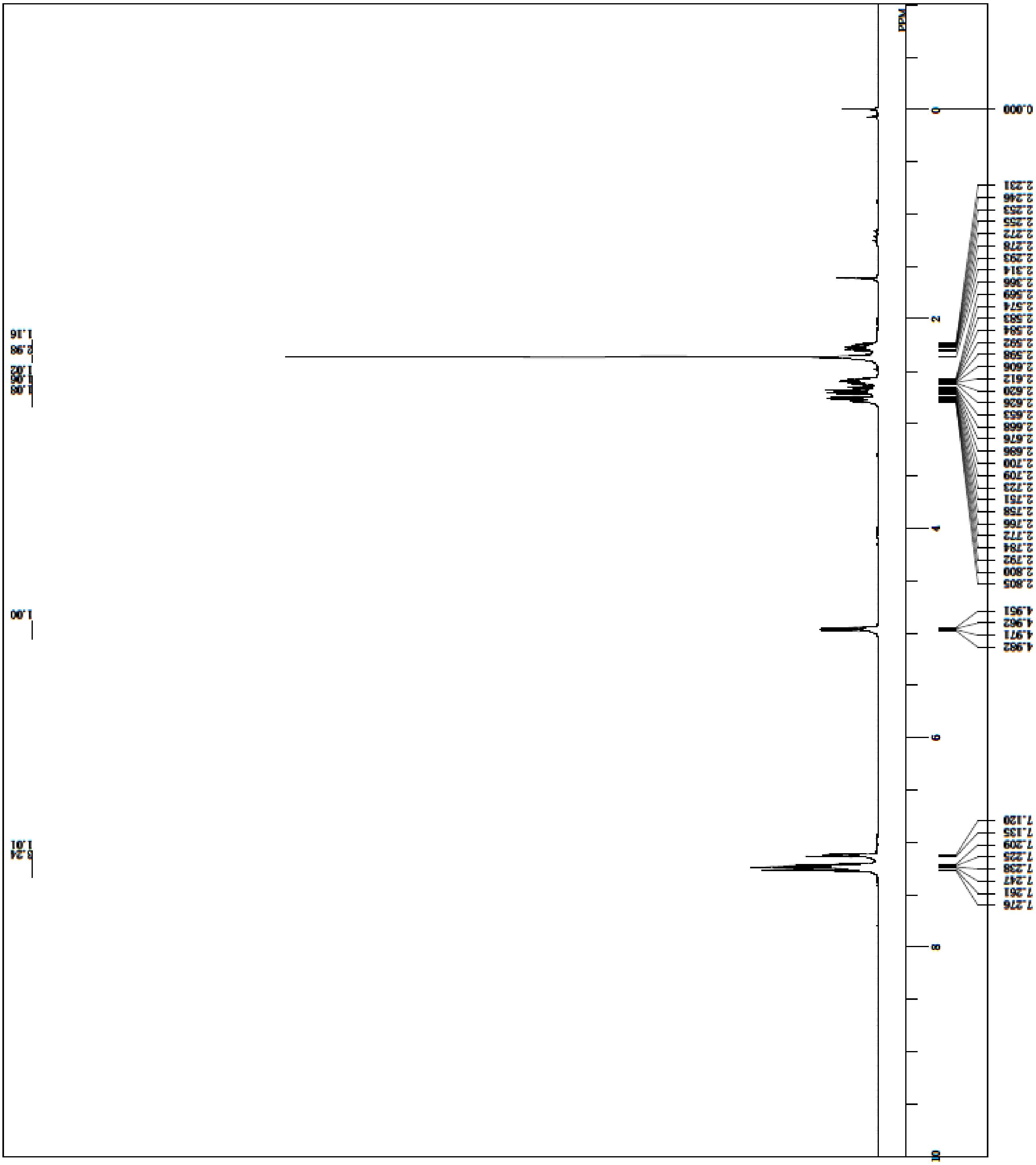

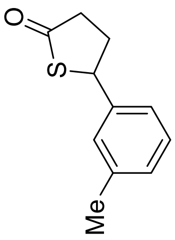
O
S
Me

2c

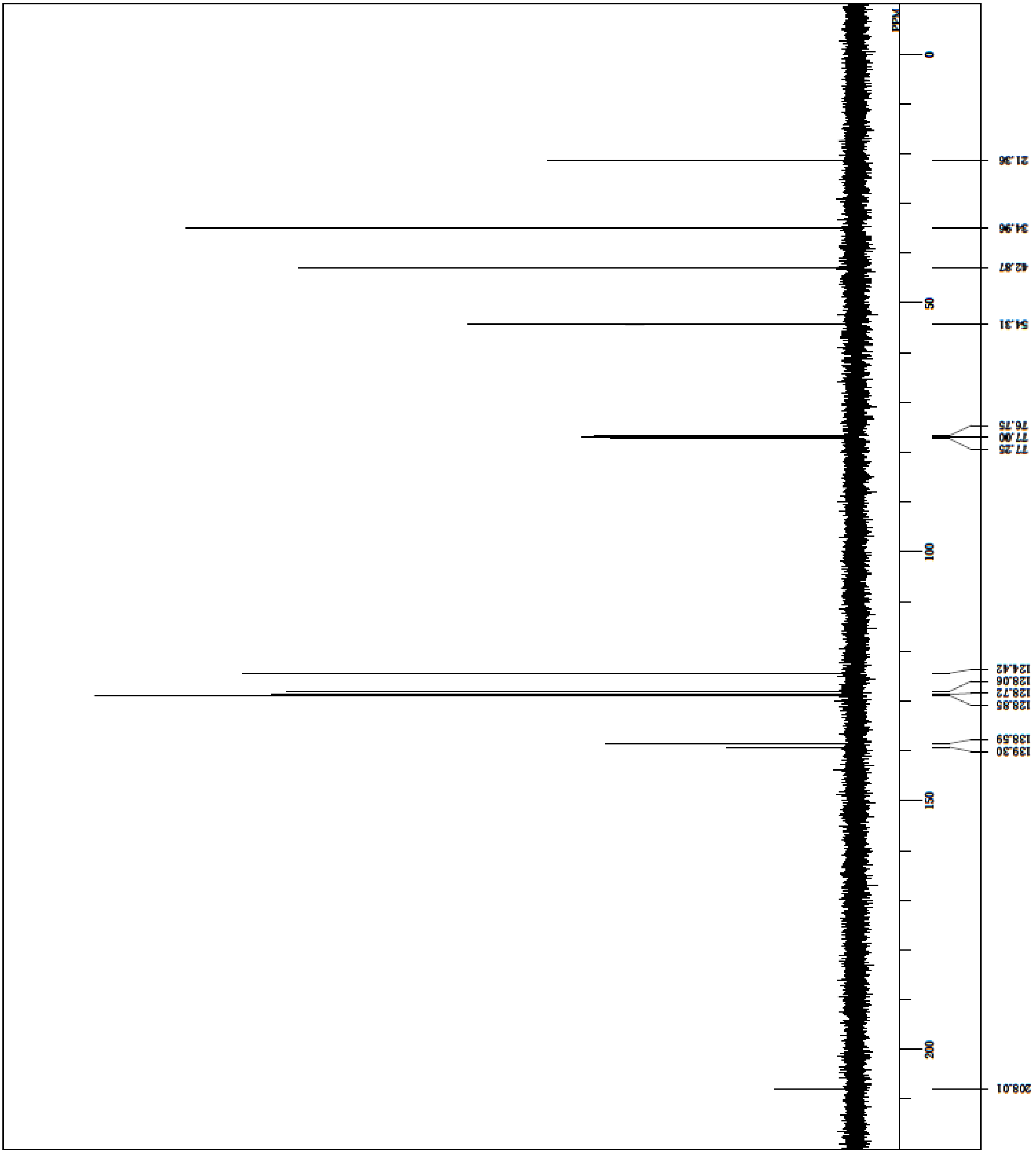
PPM
0
50
100
150
200
21.36
34.96
42.87
54.31
76.75
77.00
77.25
124.42
128.06
128.72
128.85
138.59
139.30
208.01

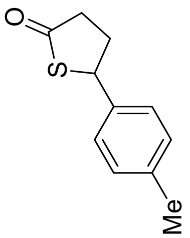

2d

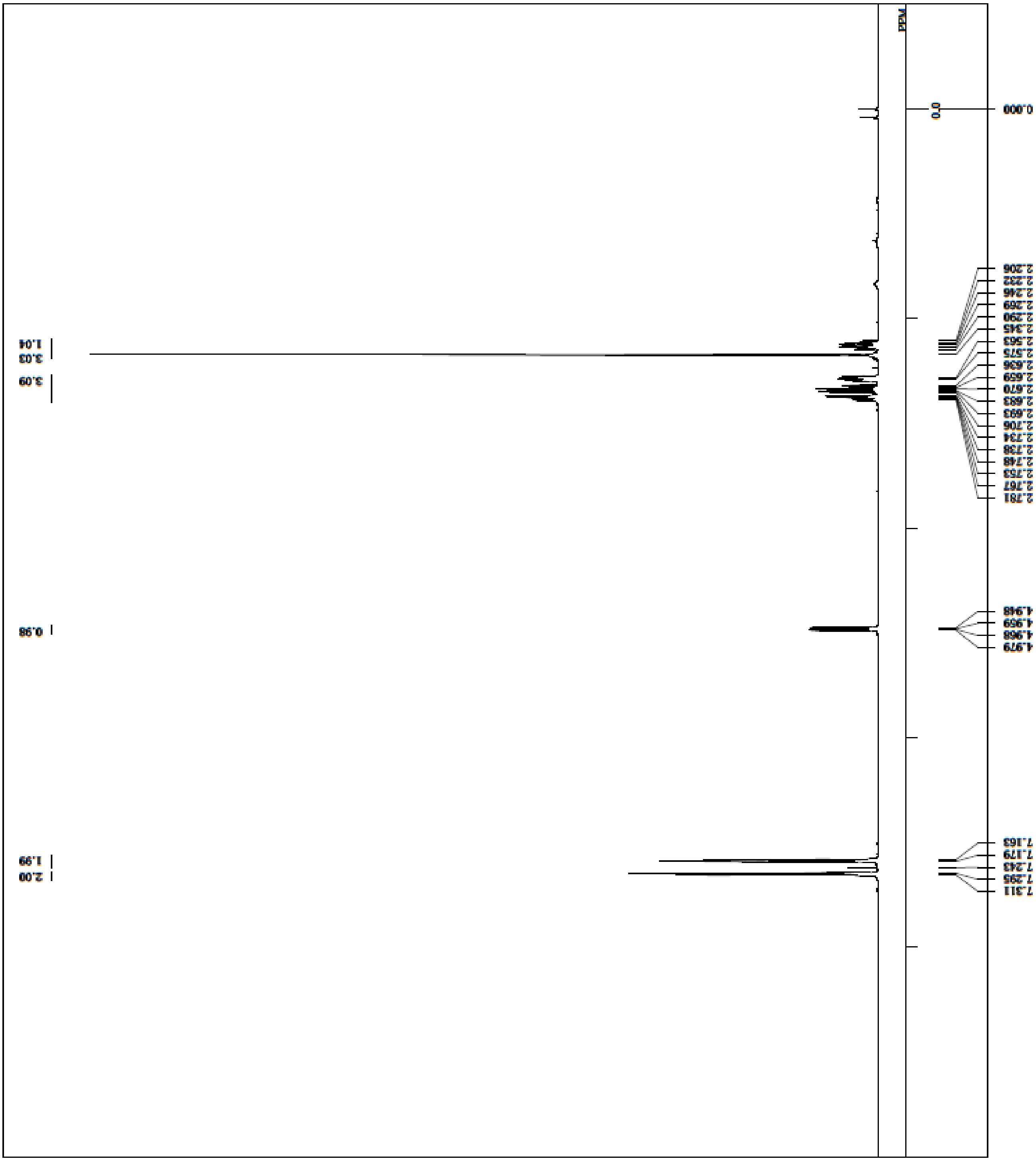

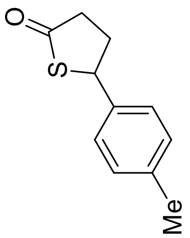
O
S
Me

2d

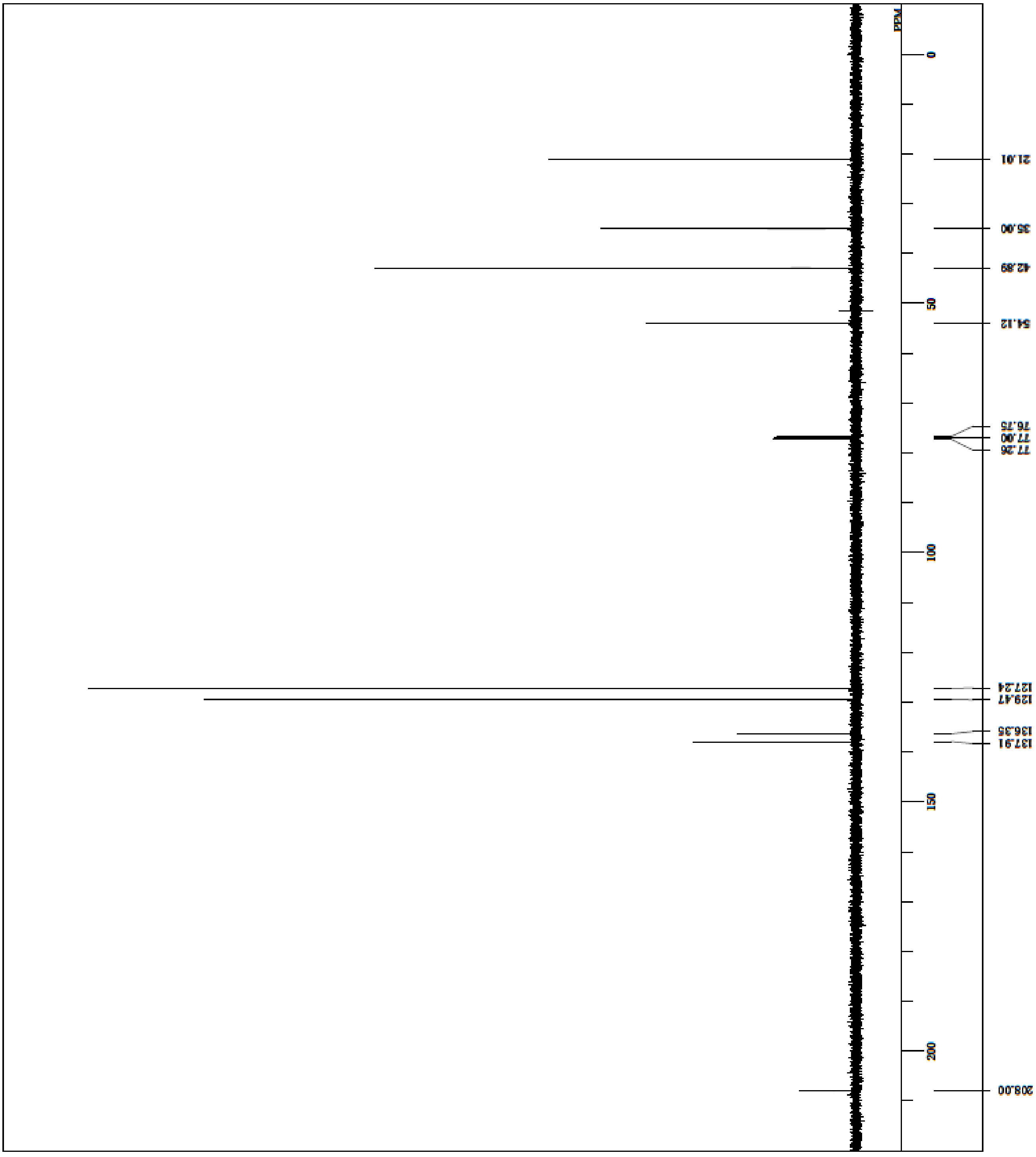
PPM
0
50
100
150
200
21.01
35.00
42.89
54.12
76.75
77.00
77.26
127.24
129.47
136.35
137.91
208.00

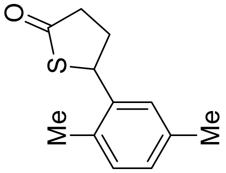

2e

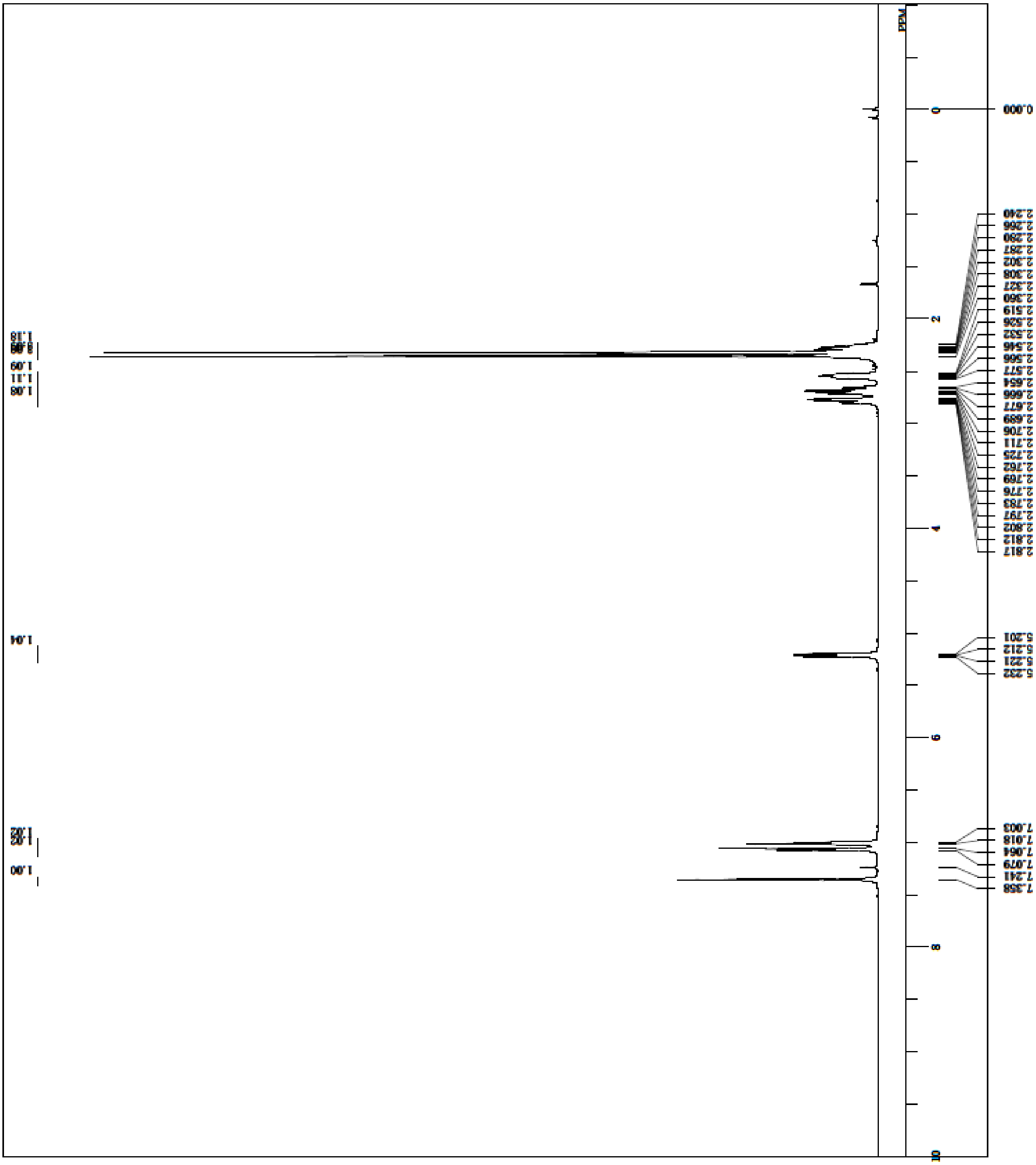

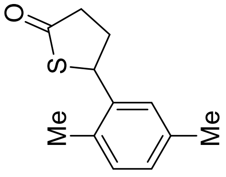
O
S
Me
Me

2e

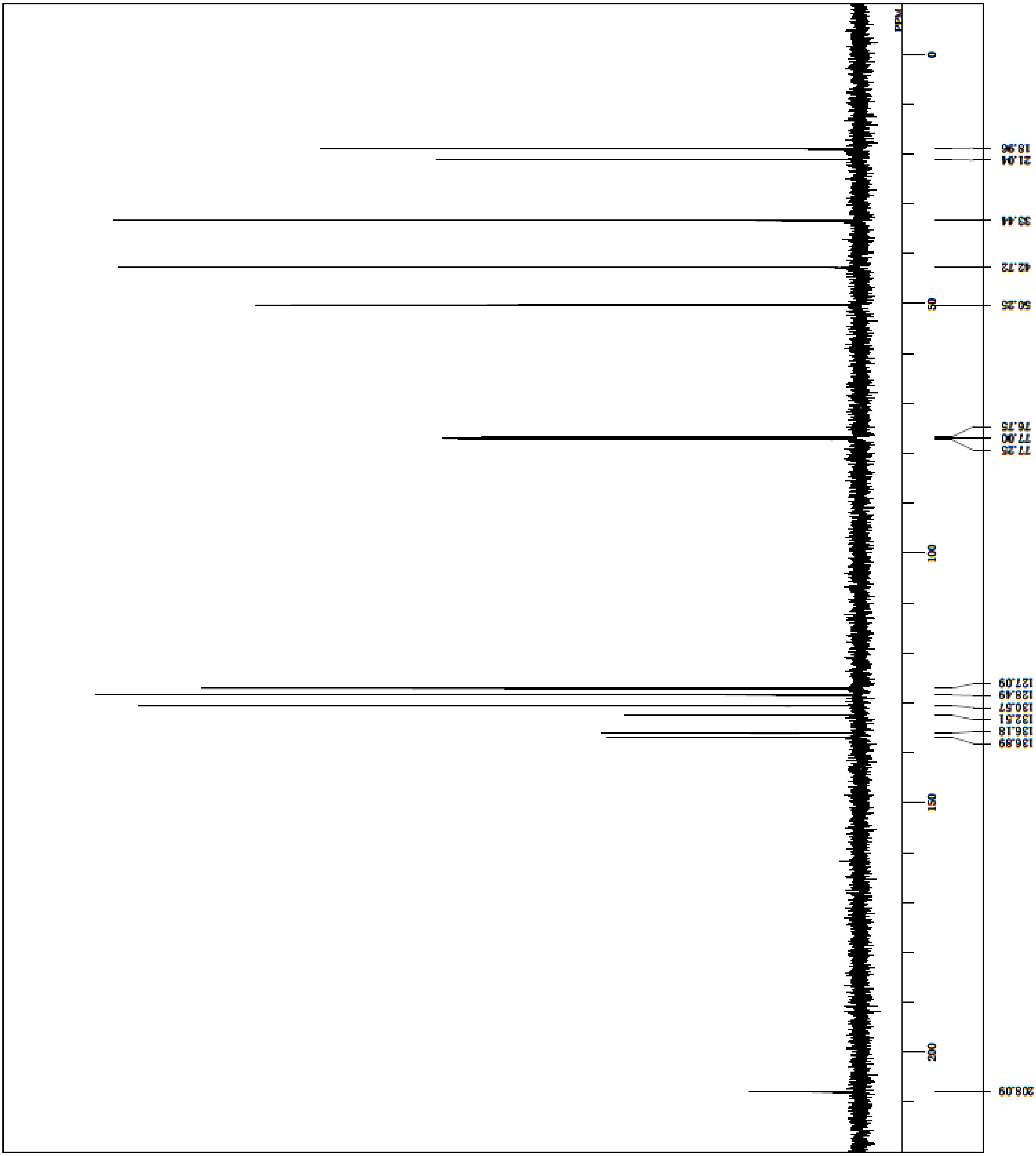
PPM
0
50
100
150
200
18.96
21.04
33.44
42.72
50.25
76.75
77.00
77.25
127.09
128.49
130.57
132.51
136.18
136.89
208.09

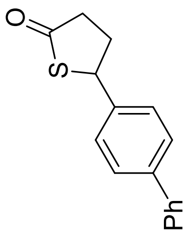

2f

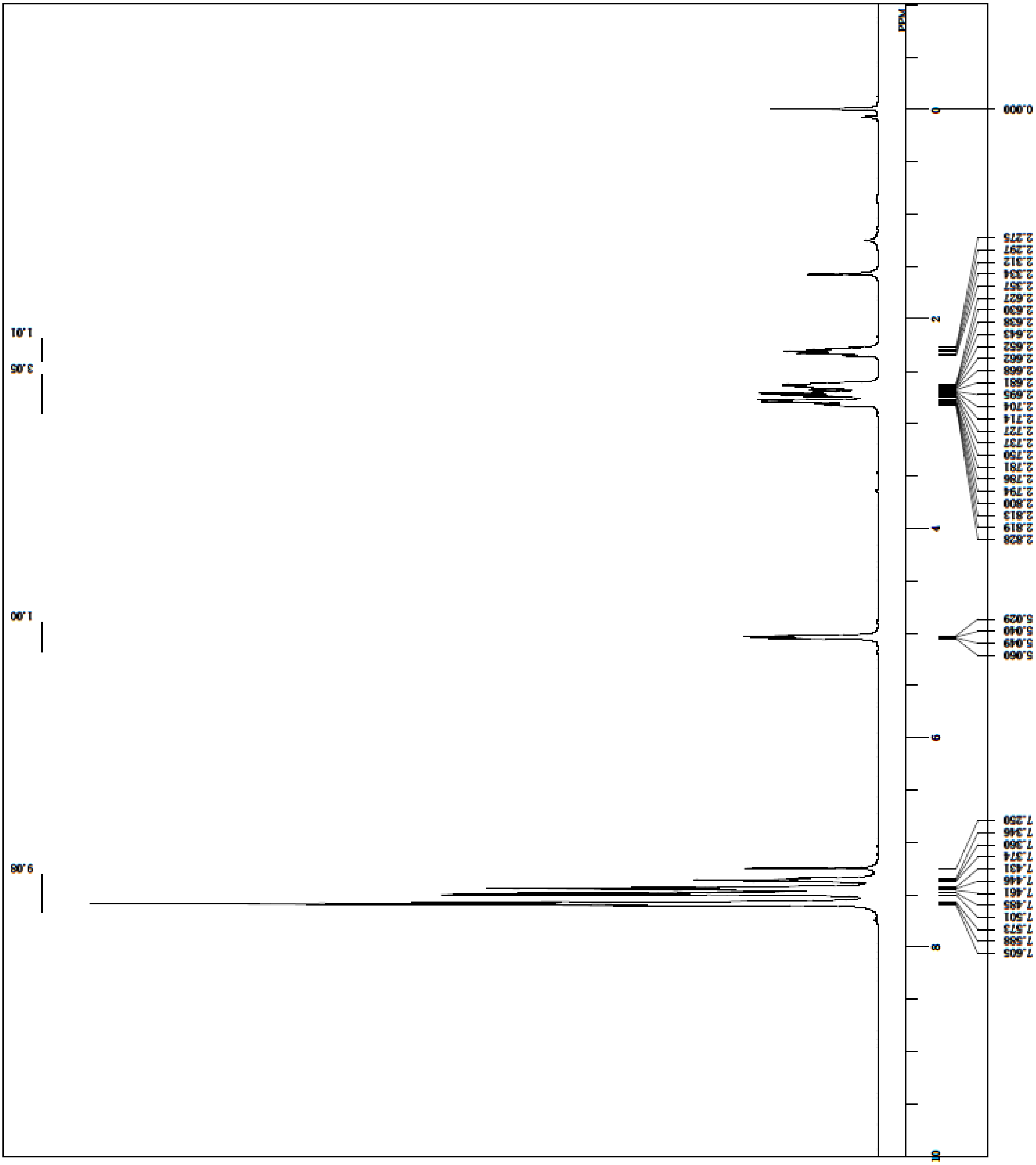

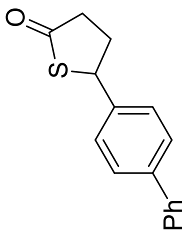

2f

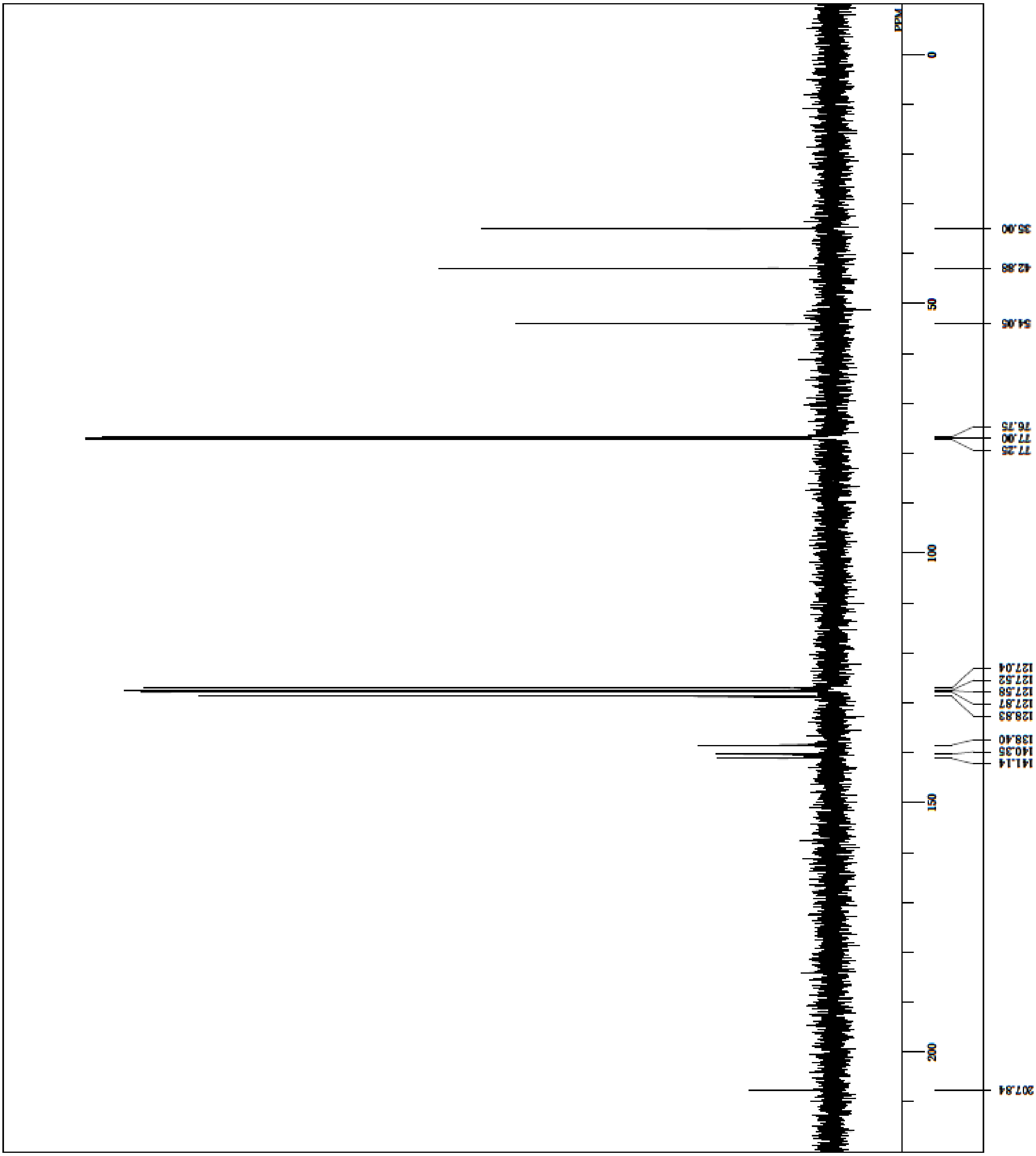
PPM
0
50
100
150
200
35.00
42.88
54.05
76.75
77.00
77.25
127.04
127.52
127.58
127.97
128.83
138.40
140.35
141.14
207.84

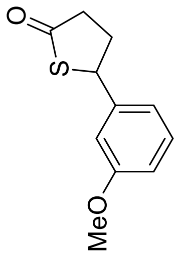

2g

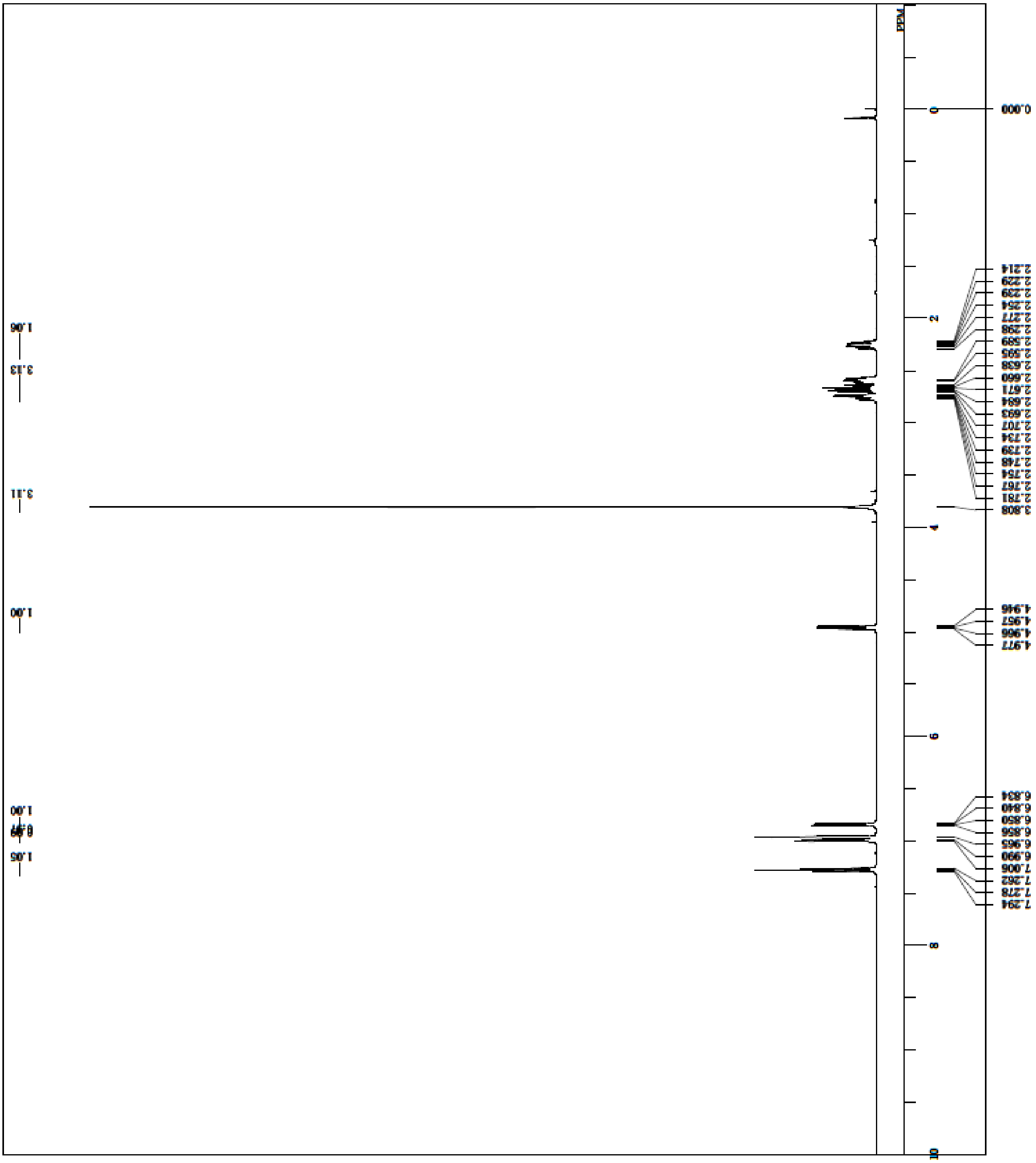

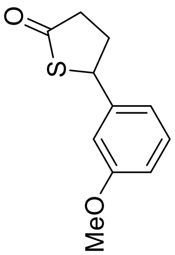
O
S
MeO

2g

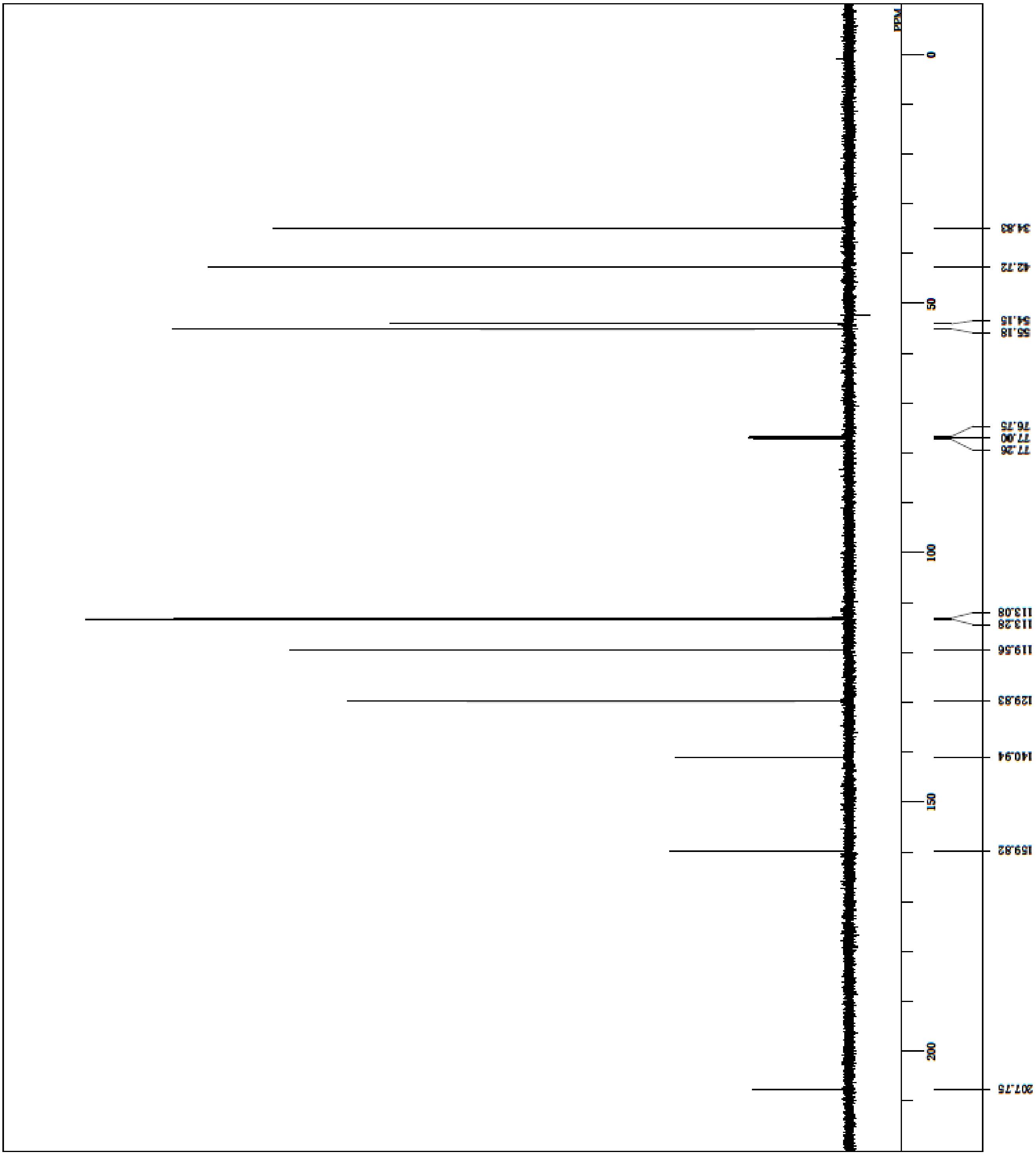
PPM
0
50
100
150
200
34.83
42.72
54.15
55.18
76.75
77.00
77.26
113.08
113.28
119.56
129.83
140.94
159.82
207.75

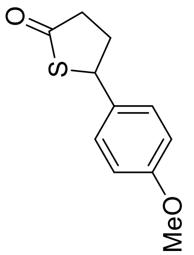

2h

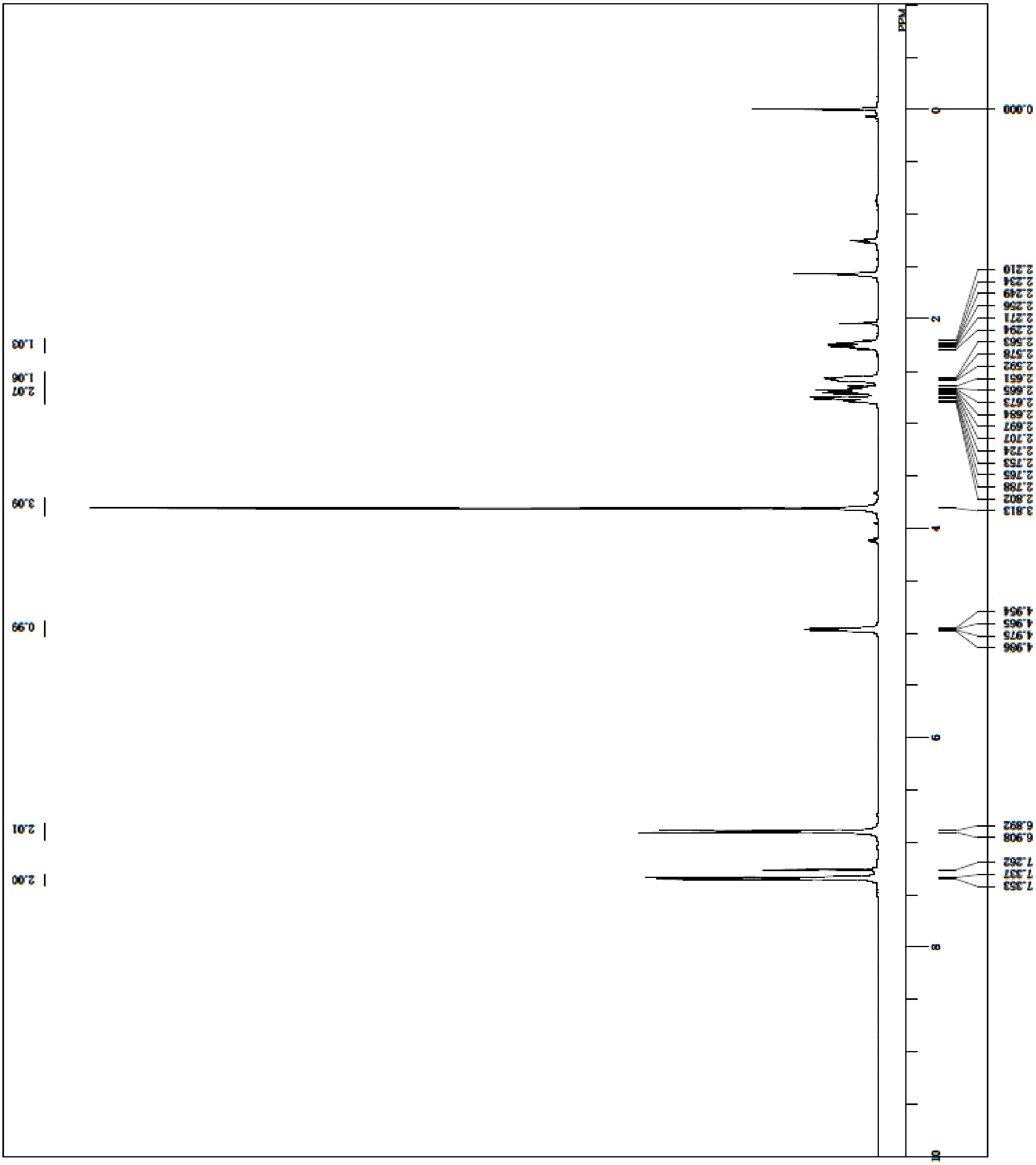

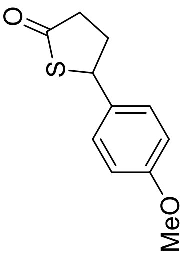

2h

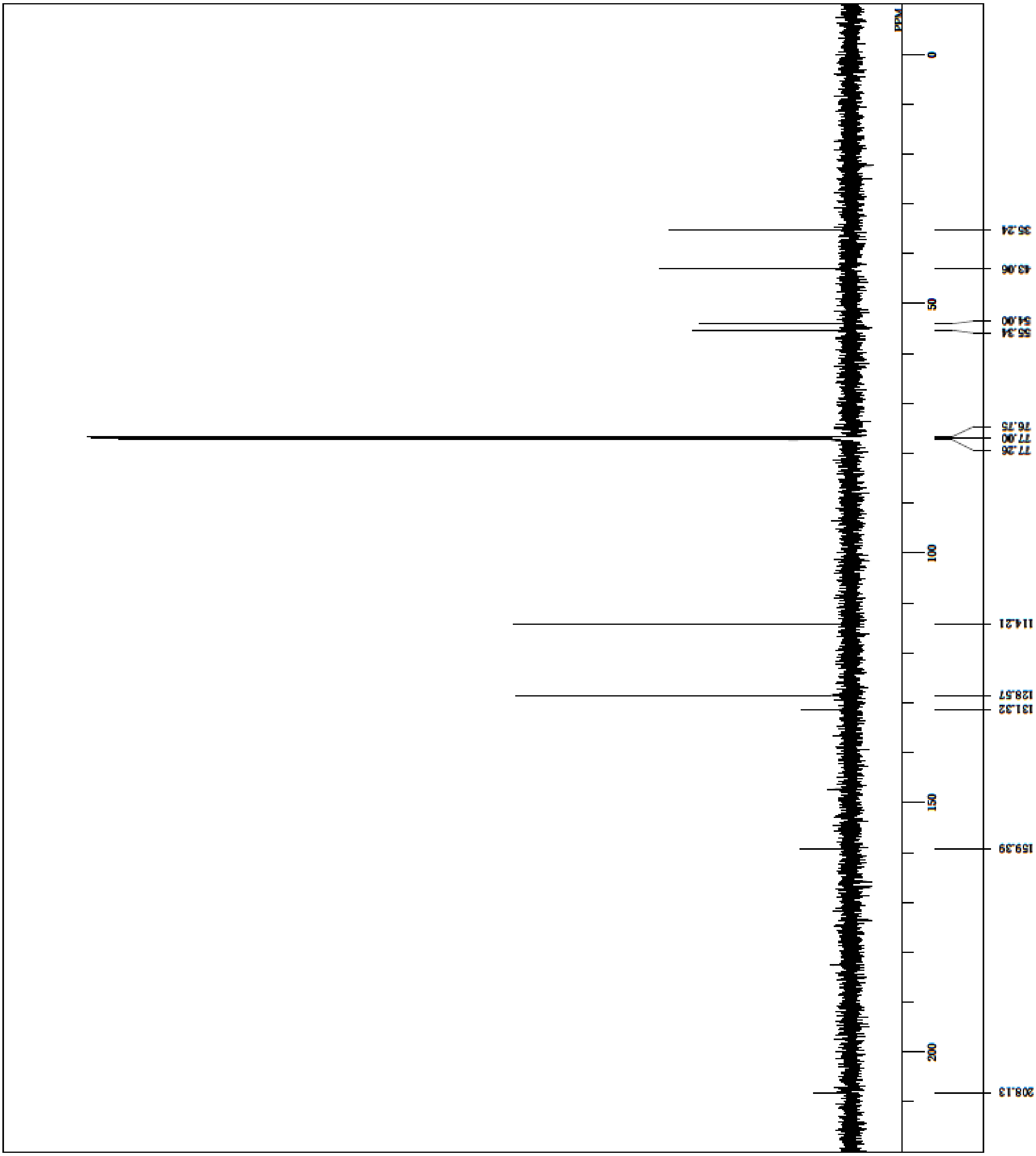

PPM
0
50
100
150
200
35.24
43.06
54.00
55.34
76.75
77.00
77.26
114.21
128.57
131.32
159.39
208.13

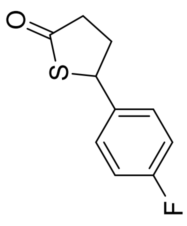

O
S
F

2i

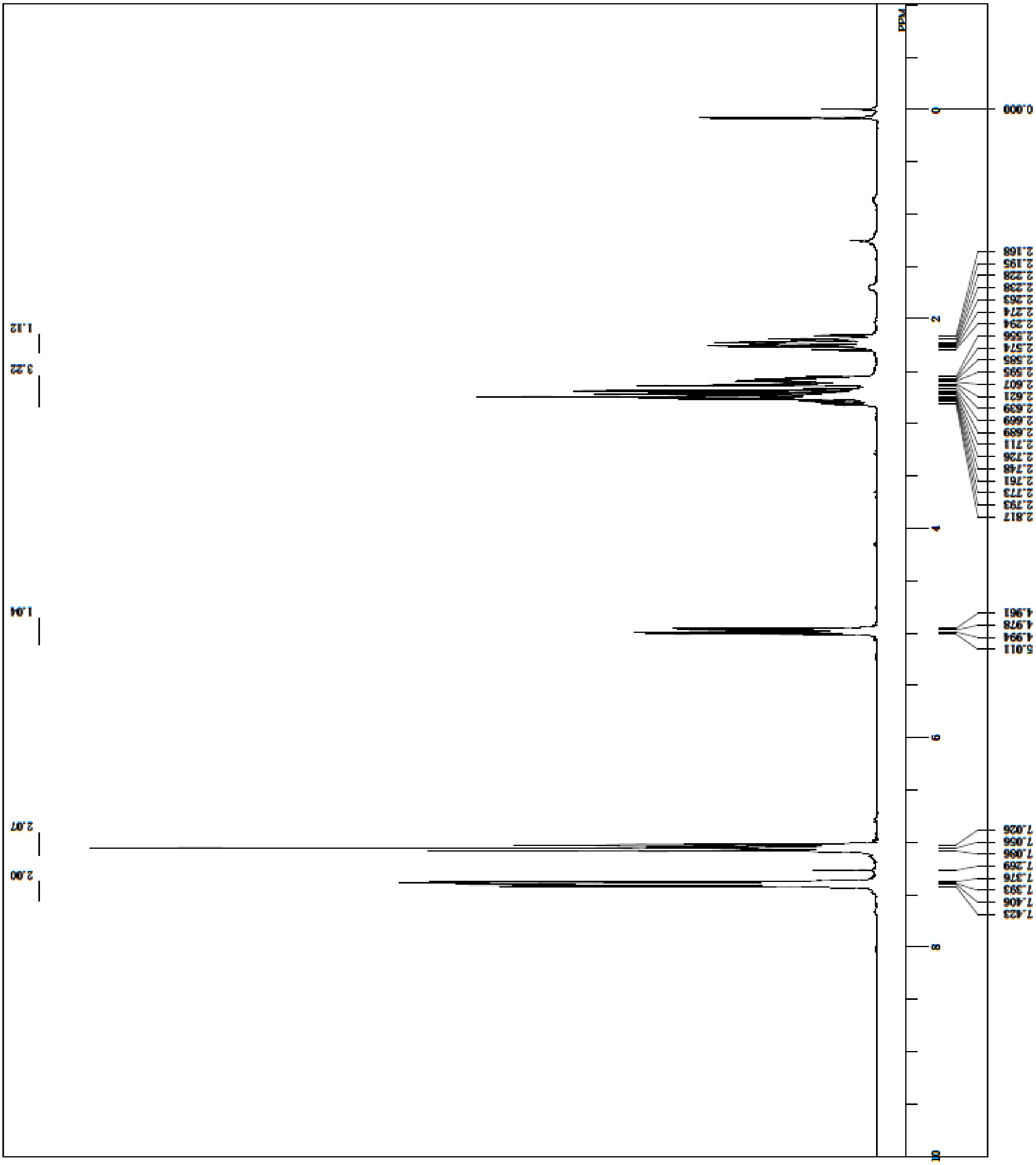

PPM
0
2
4
6
8
10
0.000
2.168
2.195
2.228
2.238
2.263
2.274
2.294
2.556
2.574
2.585
2.595
2.607
2.621
2.639
2.669
2.699
2.711
2.736
2.748
2.761
2.773
2.793
2.817
4.961
4.978
4.994
5.011
7.026
7.056
7.086
7.369
7.376
7.383
7.406
7.423
1.12
3.22
1.04
2.07
2.00

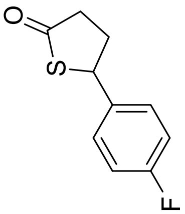

O
S
F

2i

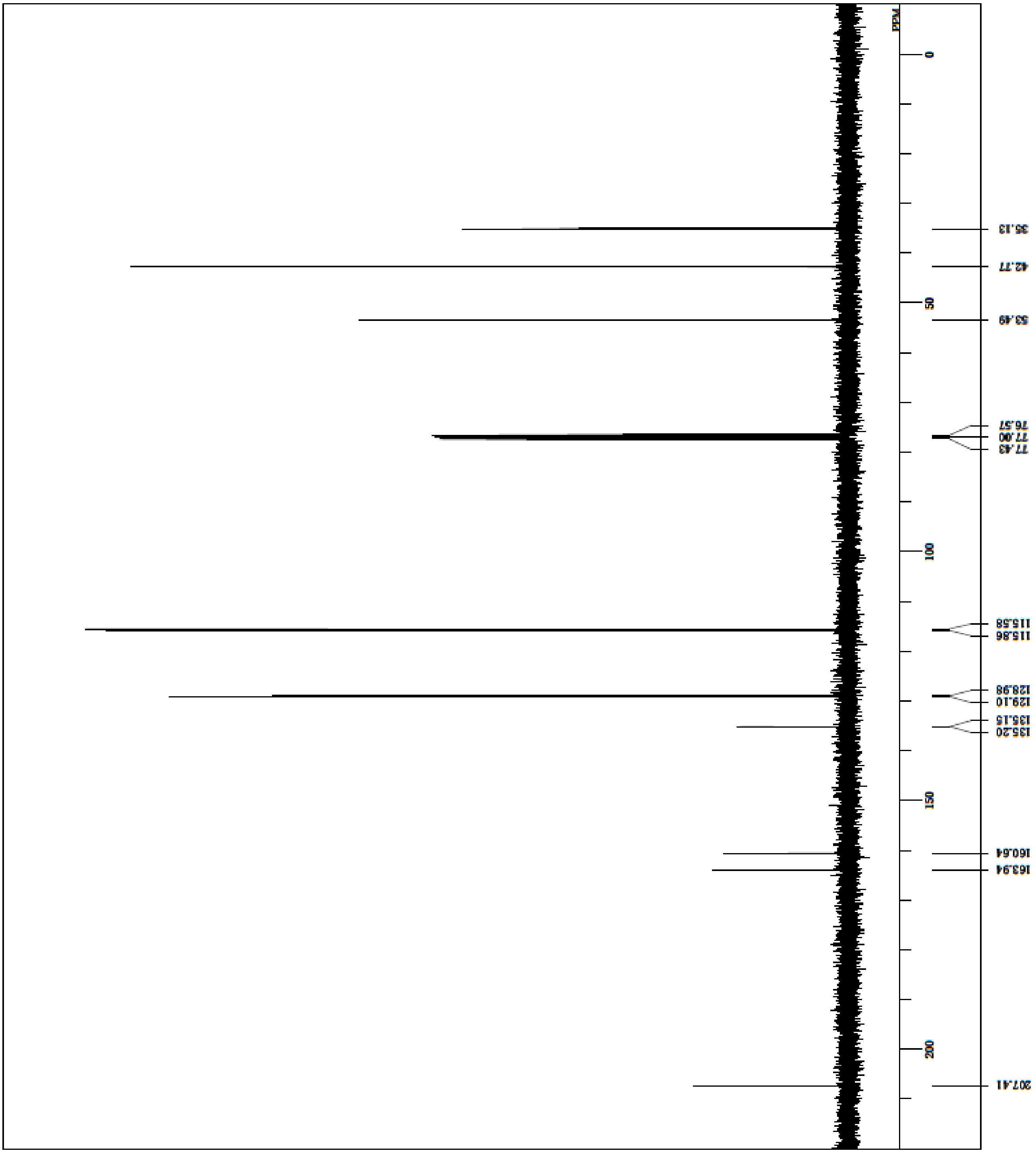

PPM
0
50
100
150
200
35.13
42.77
53.49
76.57
77.00
77.43
115.58
115.86
128.98
129.10
135.15
135.20
160.64
163.94
207.41

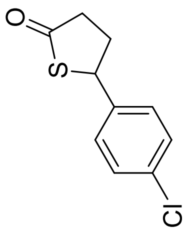
O
S
Cl

2j

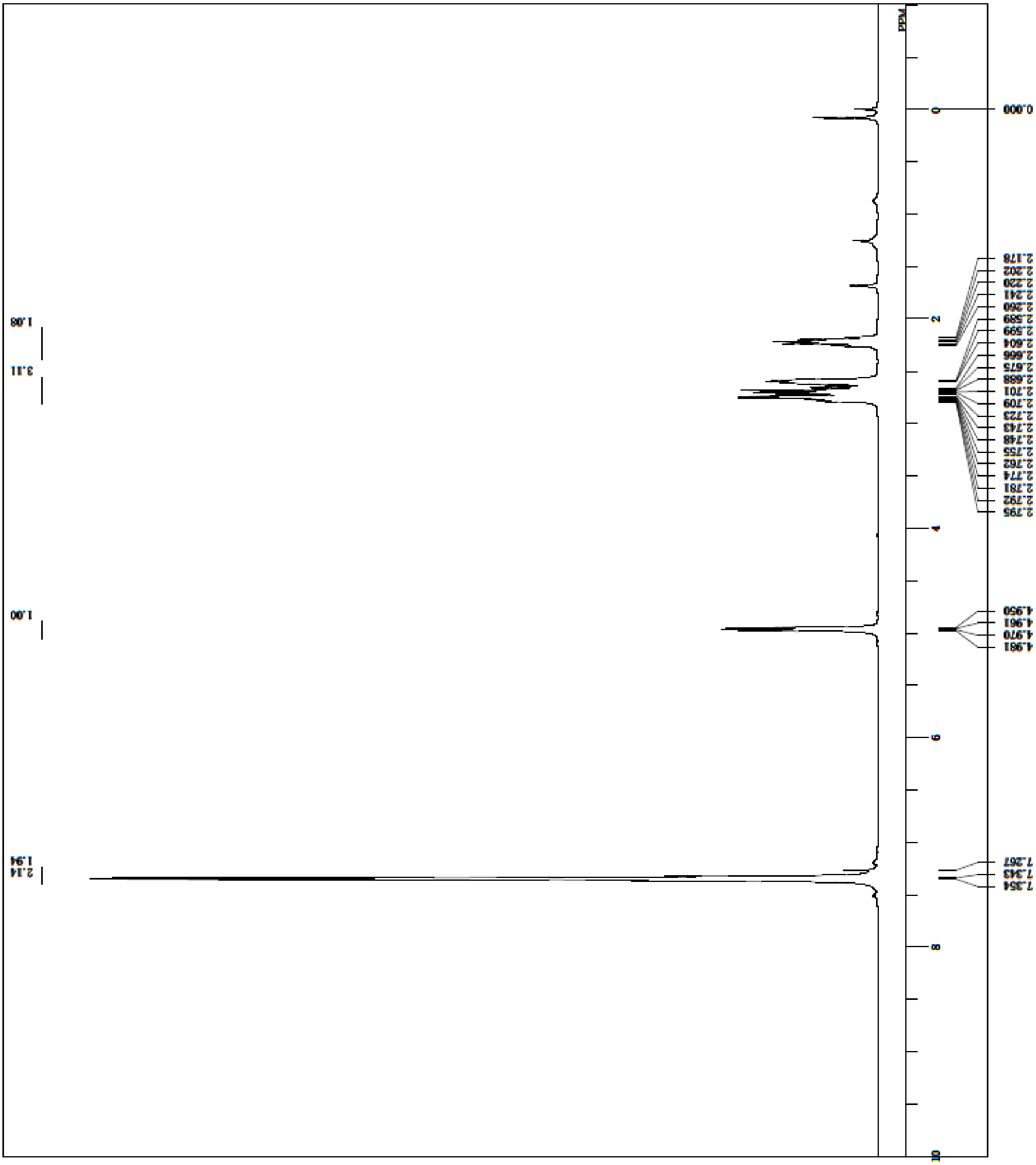
PPM
0.000
2.178
2.202
2.220
2.241
2.260
2.589
2.599
2.604
2.666
2.675
2.688
2.701
2.709
2.723
2.743
2.748
2.755
2.762
2.774
2.781
2.792
2.795
4.950
4.961
4.970
4.981
7.267
7.343
7.354
1.08
3.11
1.00
1.94
2.14

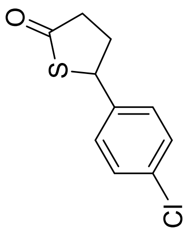
O
S
Cl

2j

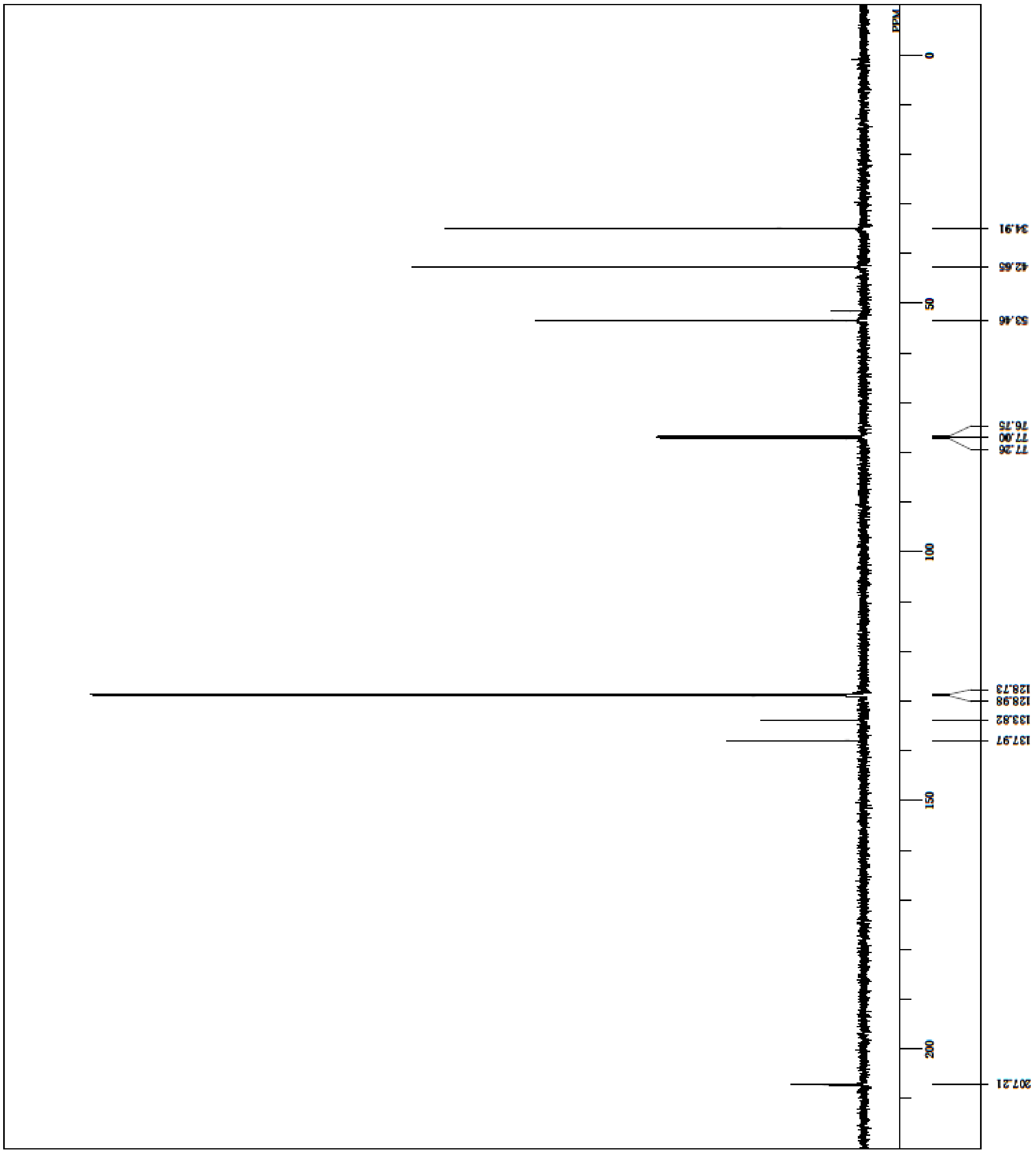
PPM
0
50
100
150
200
34.91
42.65
53.46
76.75
77.00
77.26
128.73
128.98
133.82
137.97
207.21

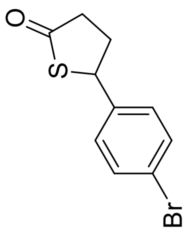
O
S
Br

2k

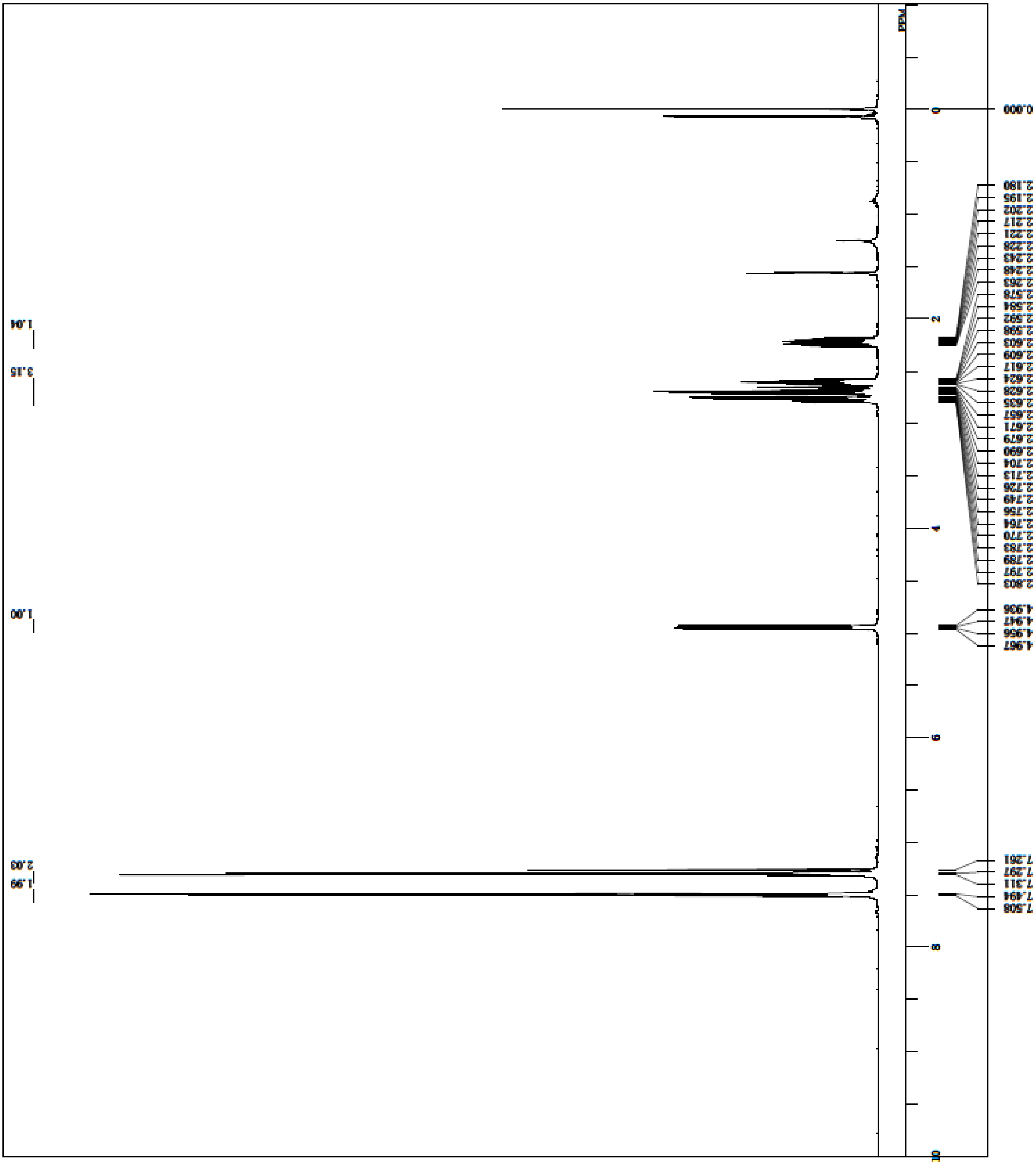
PPM
0
2
4
6
8
10
0.000
2.180
2.195
2.202
2.217
2.221
2.228
2.243
2.248
2.263
2.578
2.584
2.592
2.598
2.603
2.609
2.617
2.624
2.628
2.635
2.657
2.671
2.679
2.690
2.704
2.713
2.726
2.749
2.756
2.764
2.770
2.783
2.789
2.797
2.803
4.936
4.947
4.956
4.967
7.281
7.297
7.311
7.494
7.508
1.04
3.15
1.00
2.03
1.99

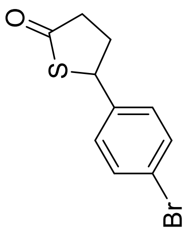
O
S
Br

2k

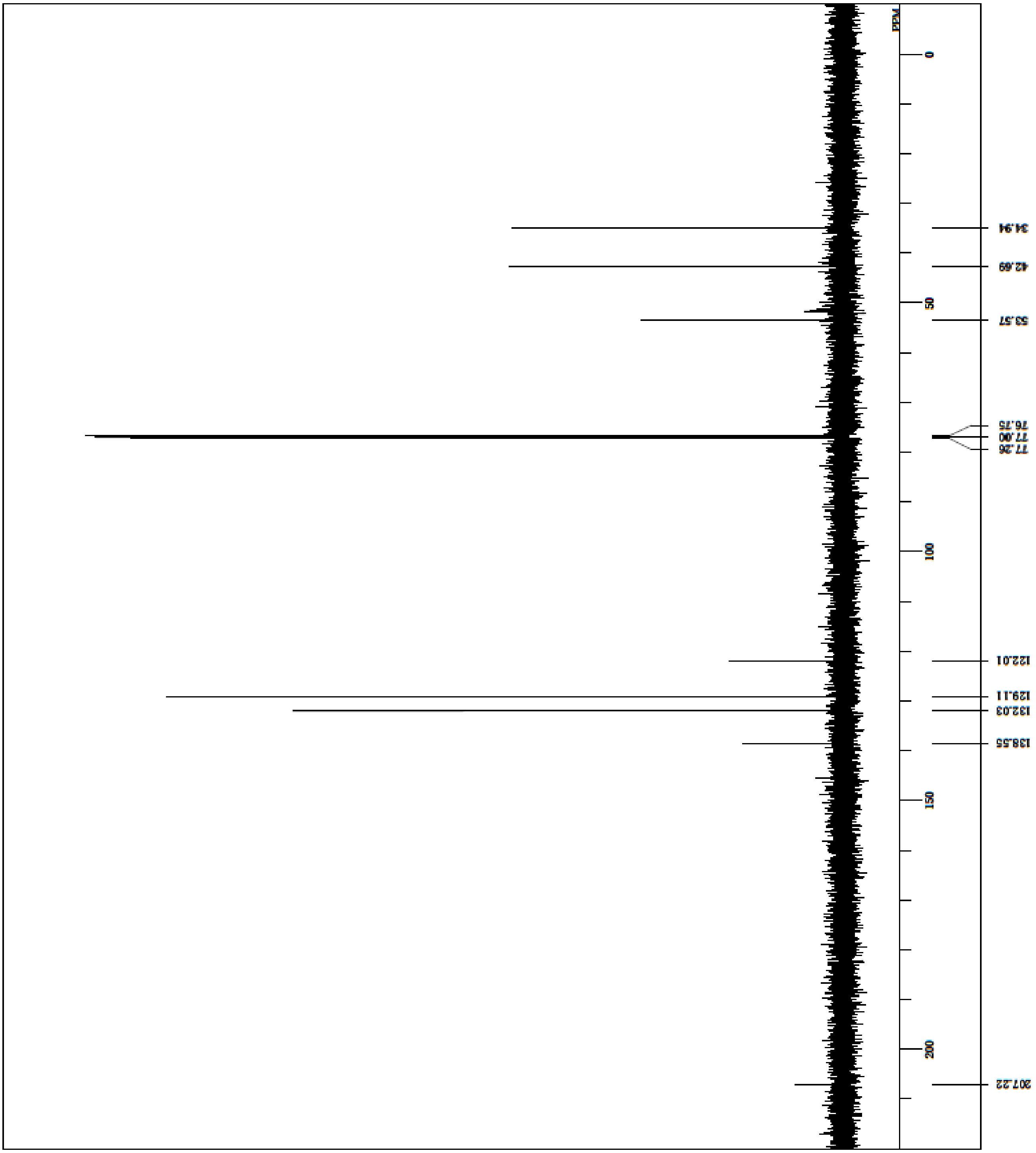
PPM
0
50
100
150
200
34.94
42.69
53.57
76.75
77.00
77.26
122.01
129.11
132.03
138.55
207.22

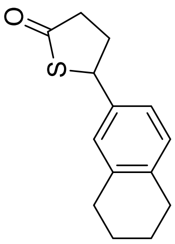

2l

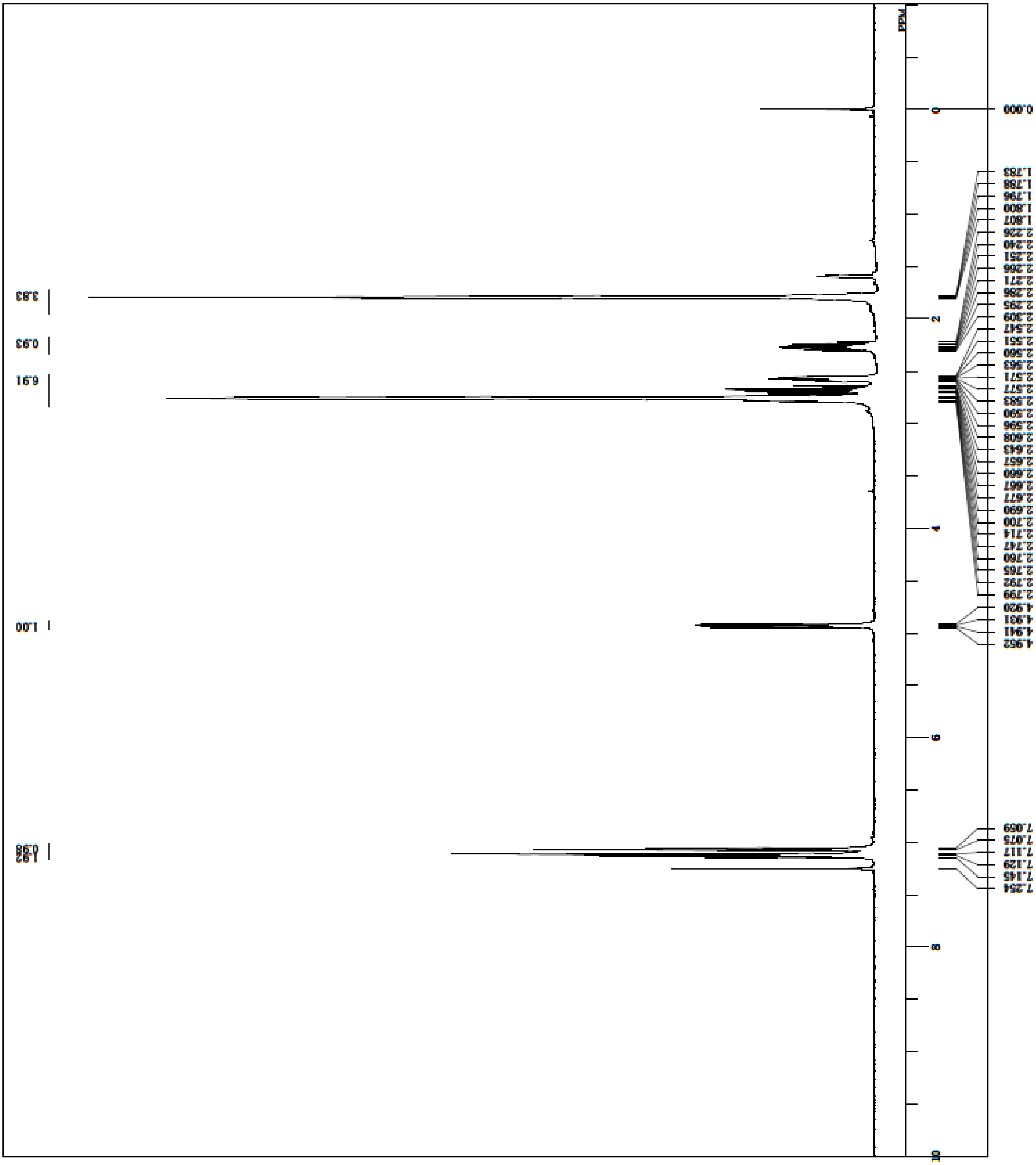

PPM
0.000
1.783
1.788
1.796
1.800
1.807
2.226
2.240
2.251
2.266
2.271
2.286
2.295
2.309
2.547
2.551
2.560
2.563
2.571
2.577
2.583
2.590
2.596
2.608
2.643
2.657
2.660
2.667
2.677
2.690
2.700
2.714
2.747
2.760
2.765
2.792
2.799
4.920
4.931
4.941
4.952
7.059
7.075
7.117
7.129
7.145
7.254
3.83
0.93
6.91
1.00
1.91
0.98

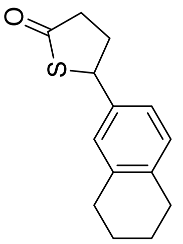
O
S

2l

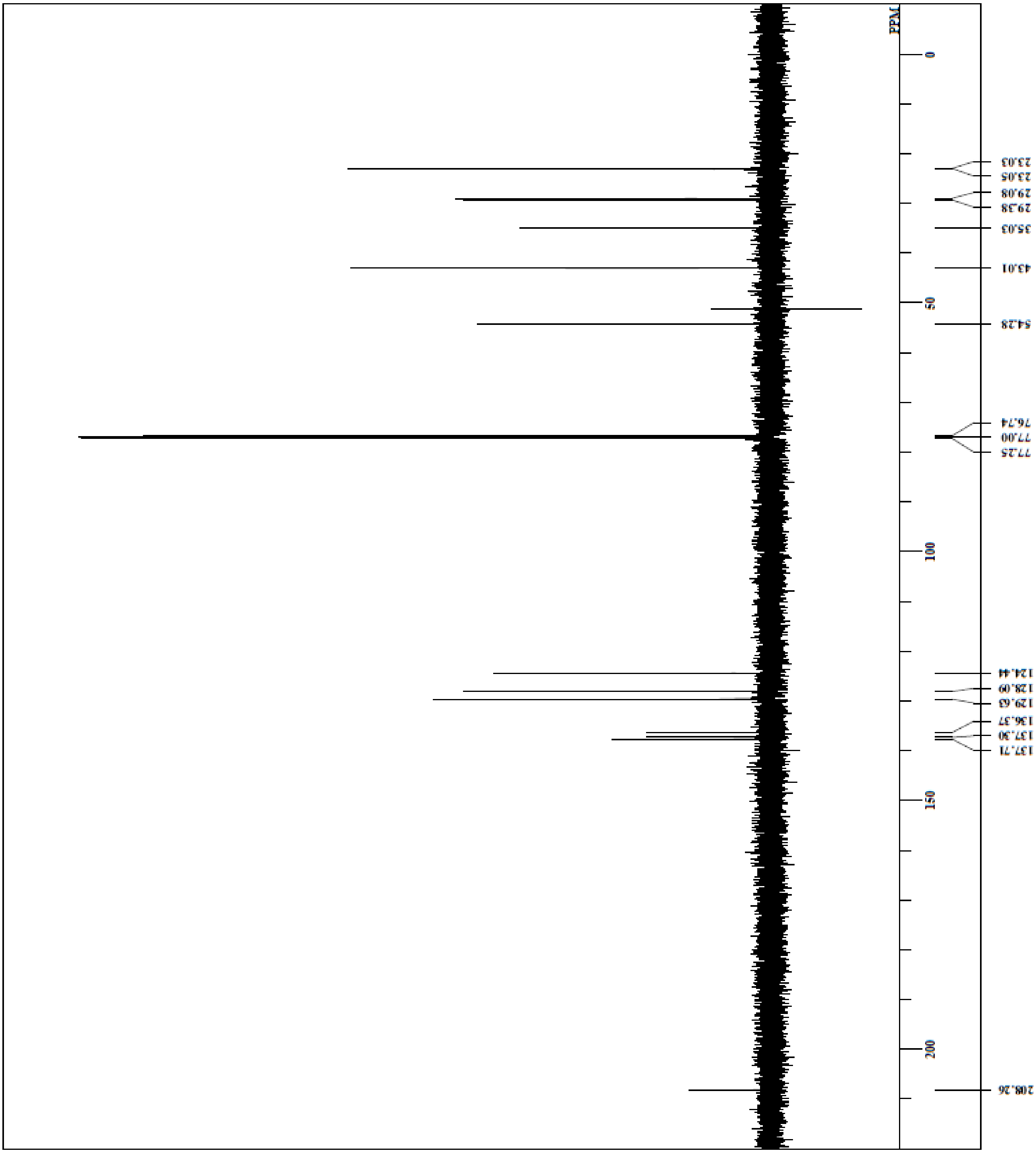
PPM
0
50
100
150
200
23.03
23.05
29.08
29.38
35.03
43.01
54.28
76.74
77.00
77.25
124.44
128.09
129.63
136.37
137.30
137.71
208.26

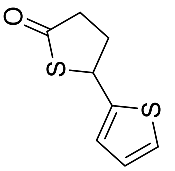

2m

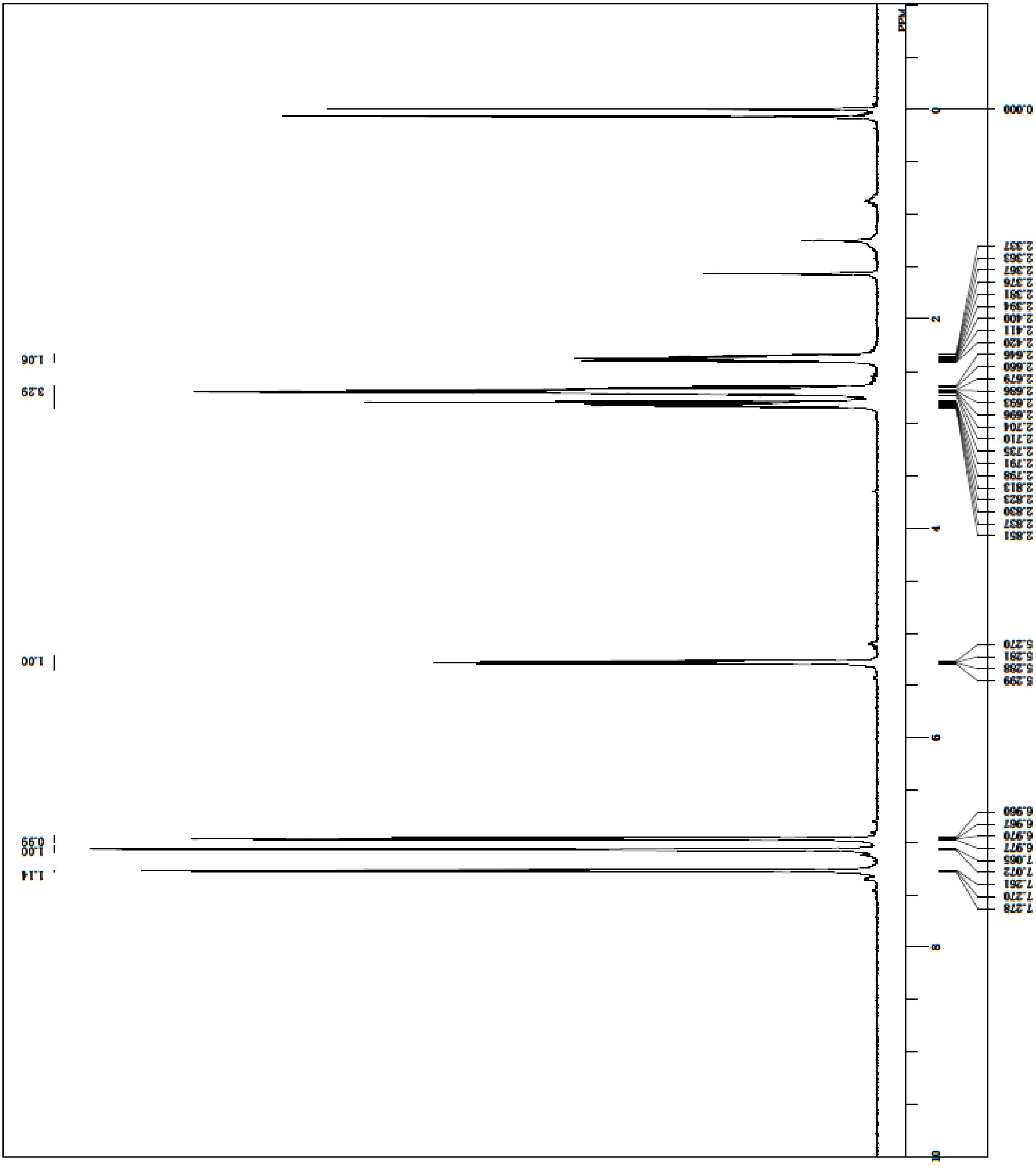

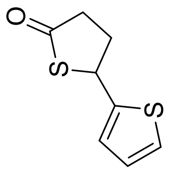

2m

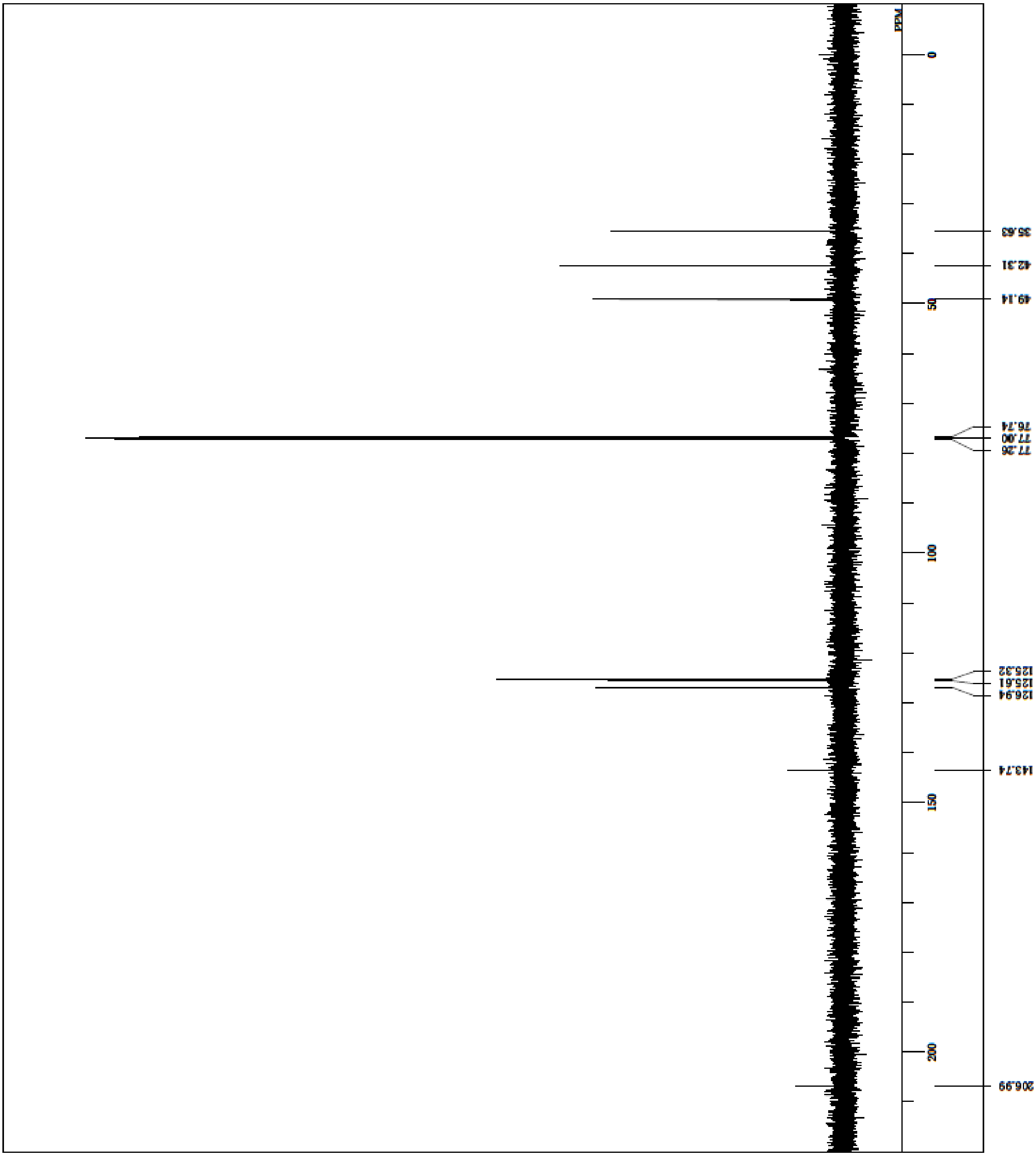
PPM
0
50
100
150
200
35.63
42.31
49.14
76.74
77.00
77.26
125.32
125.61
126.94
143.74
206.99

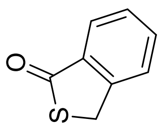
O
S

2o

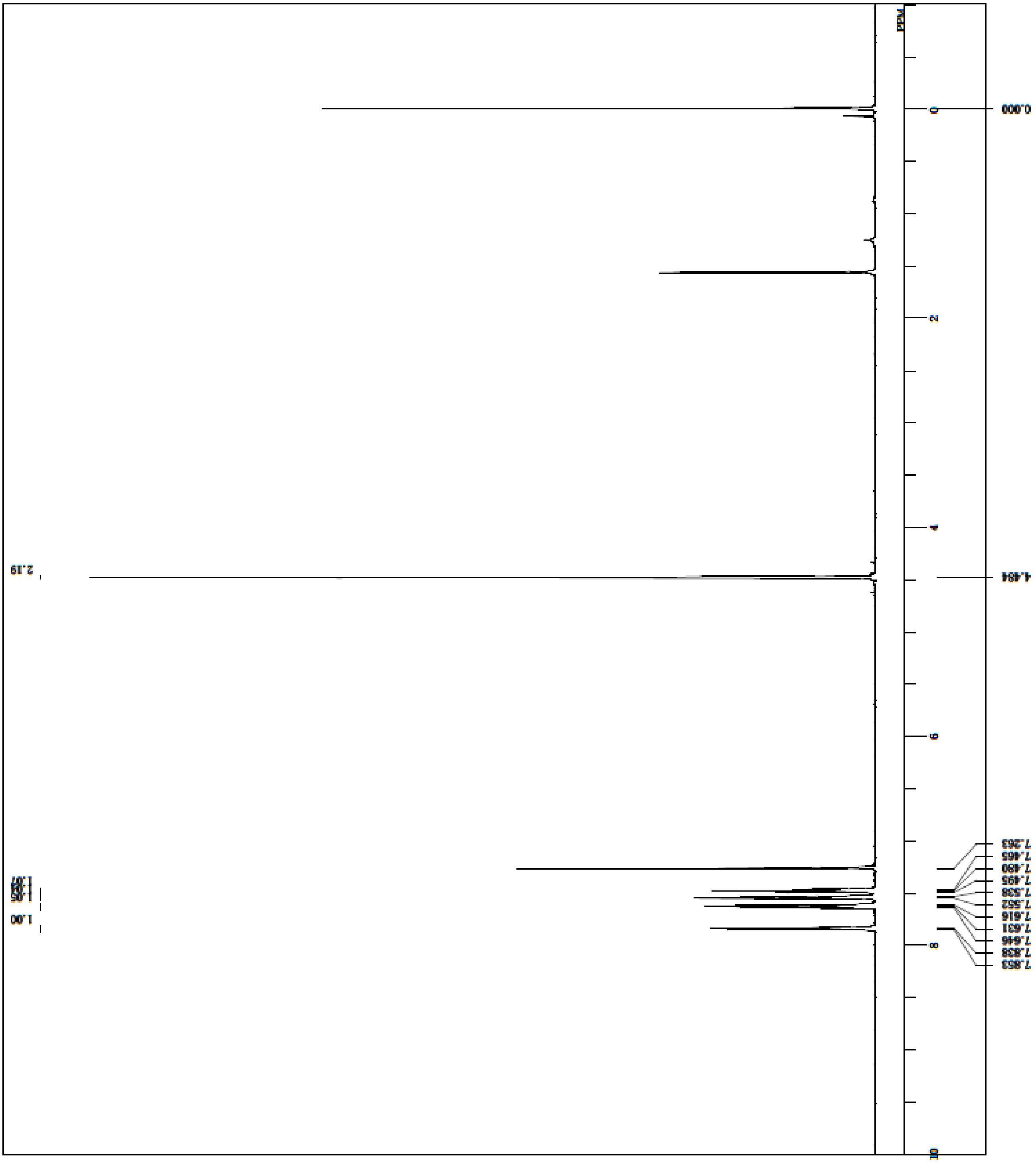
PPM
0.000
4.494
7.353
7.465
7.480
7.495
7.538
7.552
7.616
7.631
7.646
7.838
7.853
2.19
1.00

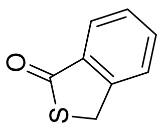

2o

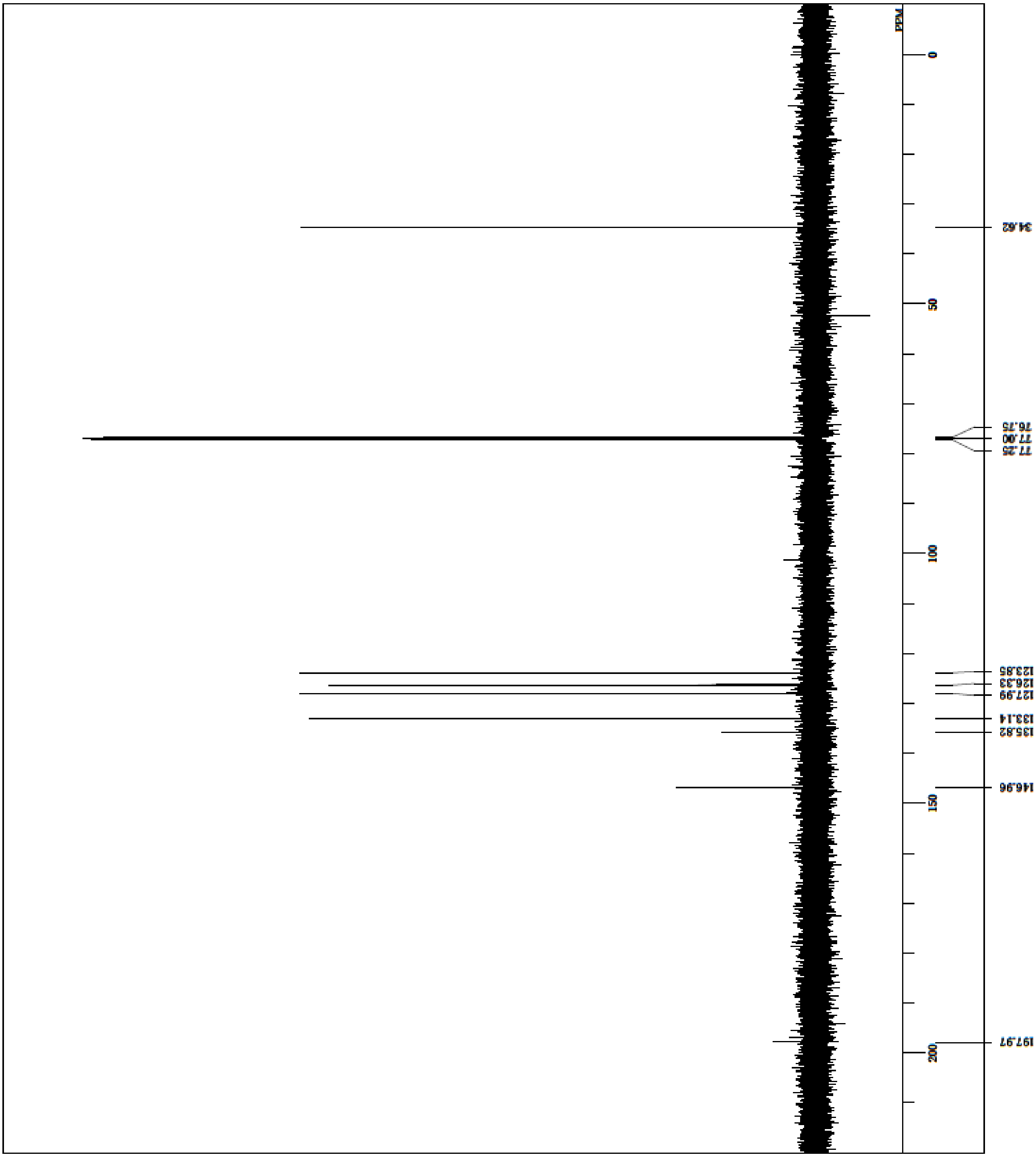

PPM
0
50
100
150
200
34.62
76.75
77.00
77.25
123.85
126.33
127.99
133.14
135.82
146.96
197.97

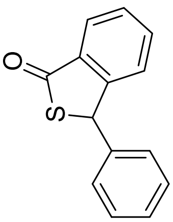
O
S

2p

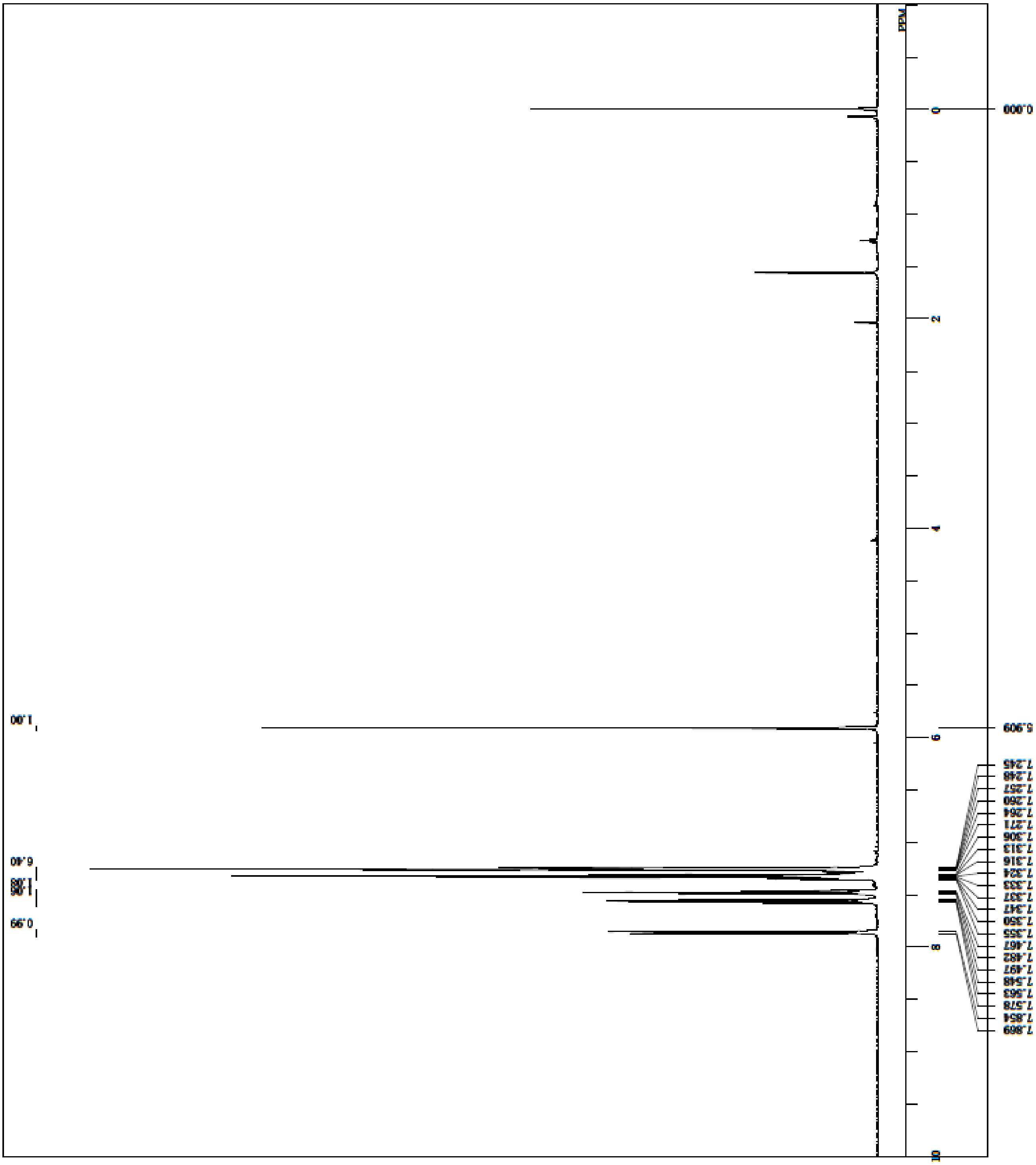
PPM
0
2
4
6
8
10
0.000
5.909
7.245
7.248
7.257
7.260
7.264
7.271
7.306
7.313
7.316
7.324
7.333
7.337
7.347
7.350
7.355
7.467
7.482
7.497
7.548
7.563
7.578
7.854
7.869
1.00
6.40
0.99

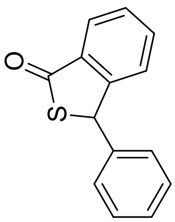
O
S

2p

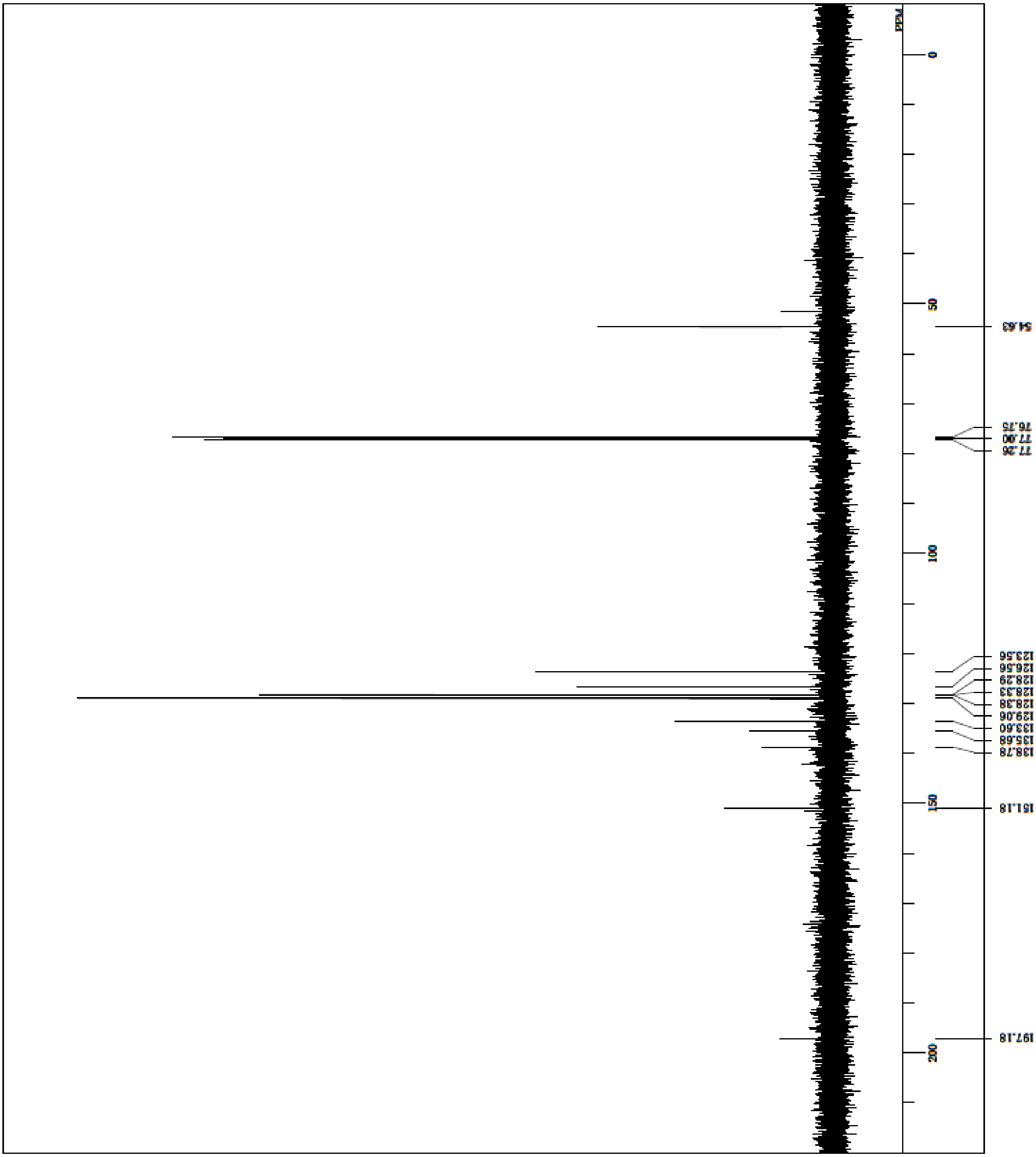
PPM
0
50
100
150
200
54.63
76.75
77.00
77.26
123.56
126.56
128.29
128.33
128.38
129.06
133.60
135.68
138.78
151.18
197.18

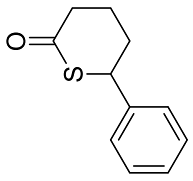

2q

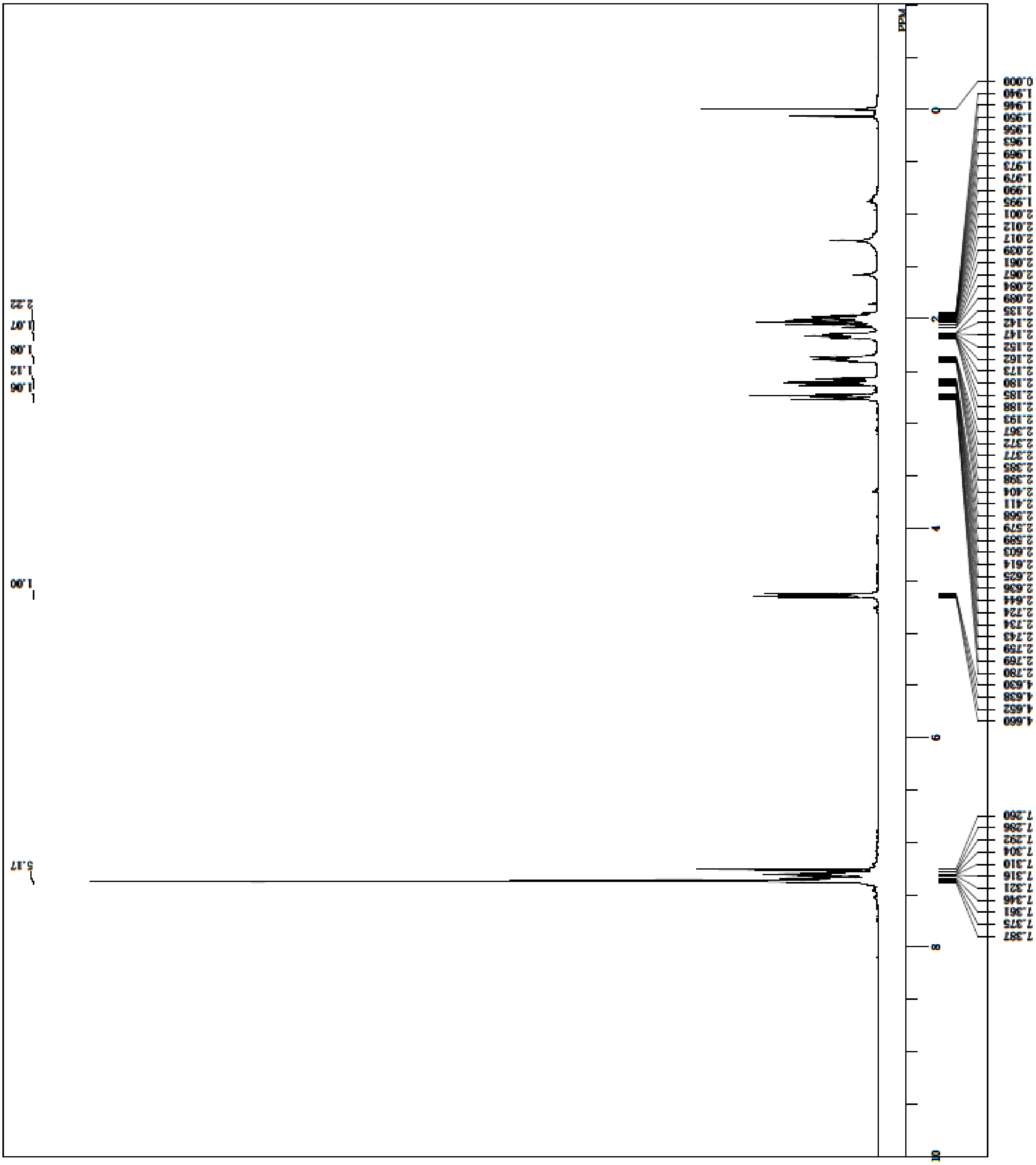

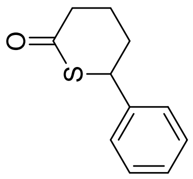

2q

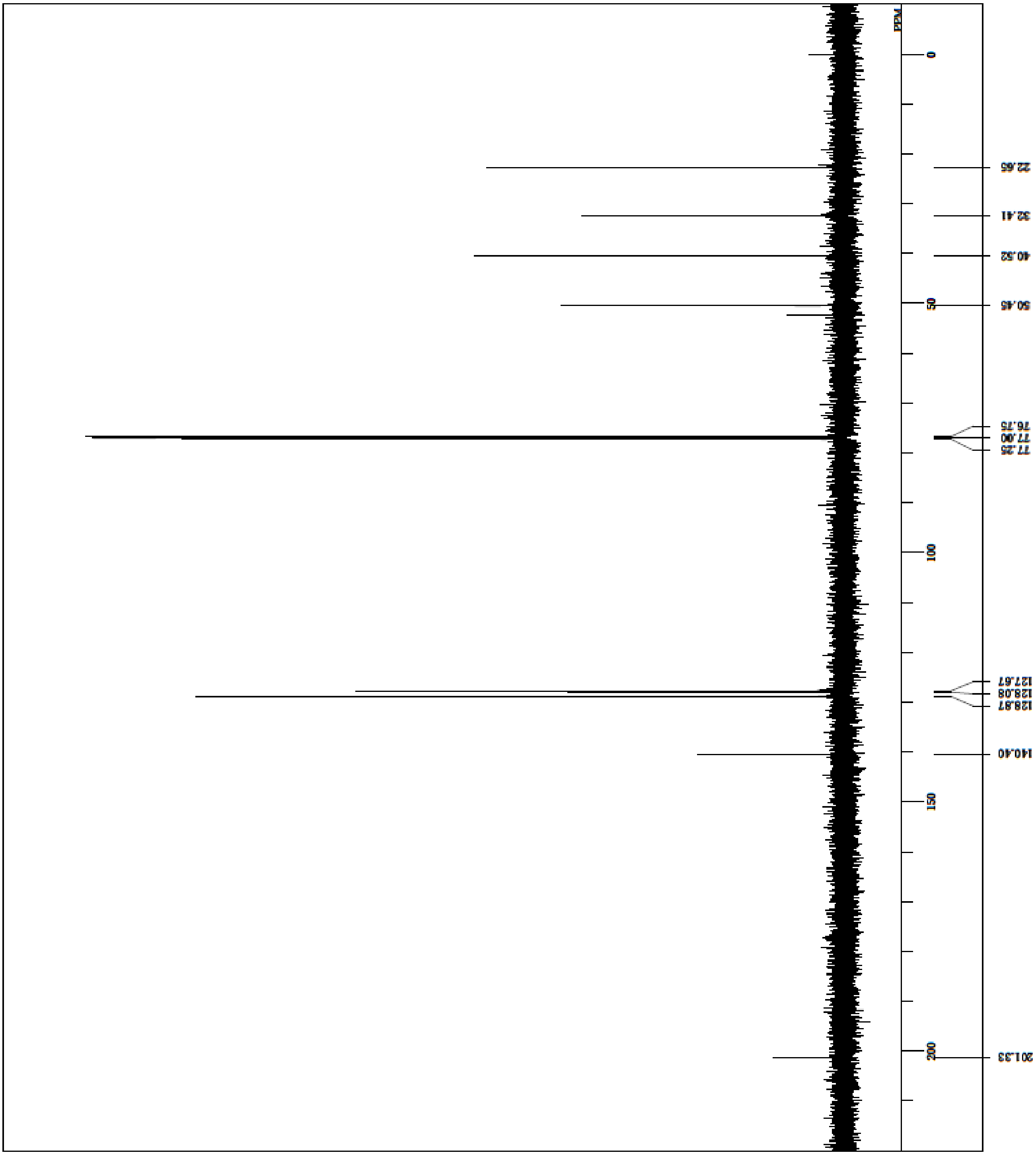
PPM
0
50
100
150
200
22.65
32.41
40.52
50.45
76.75
77.00
77.25
127.67
128.08
128.87
140.40
201.33

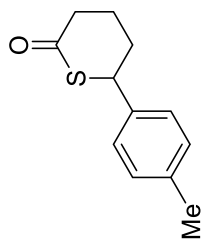

O
S
Me

2r

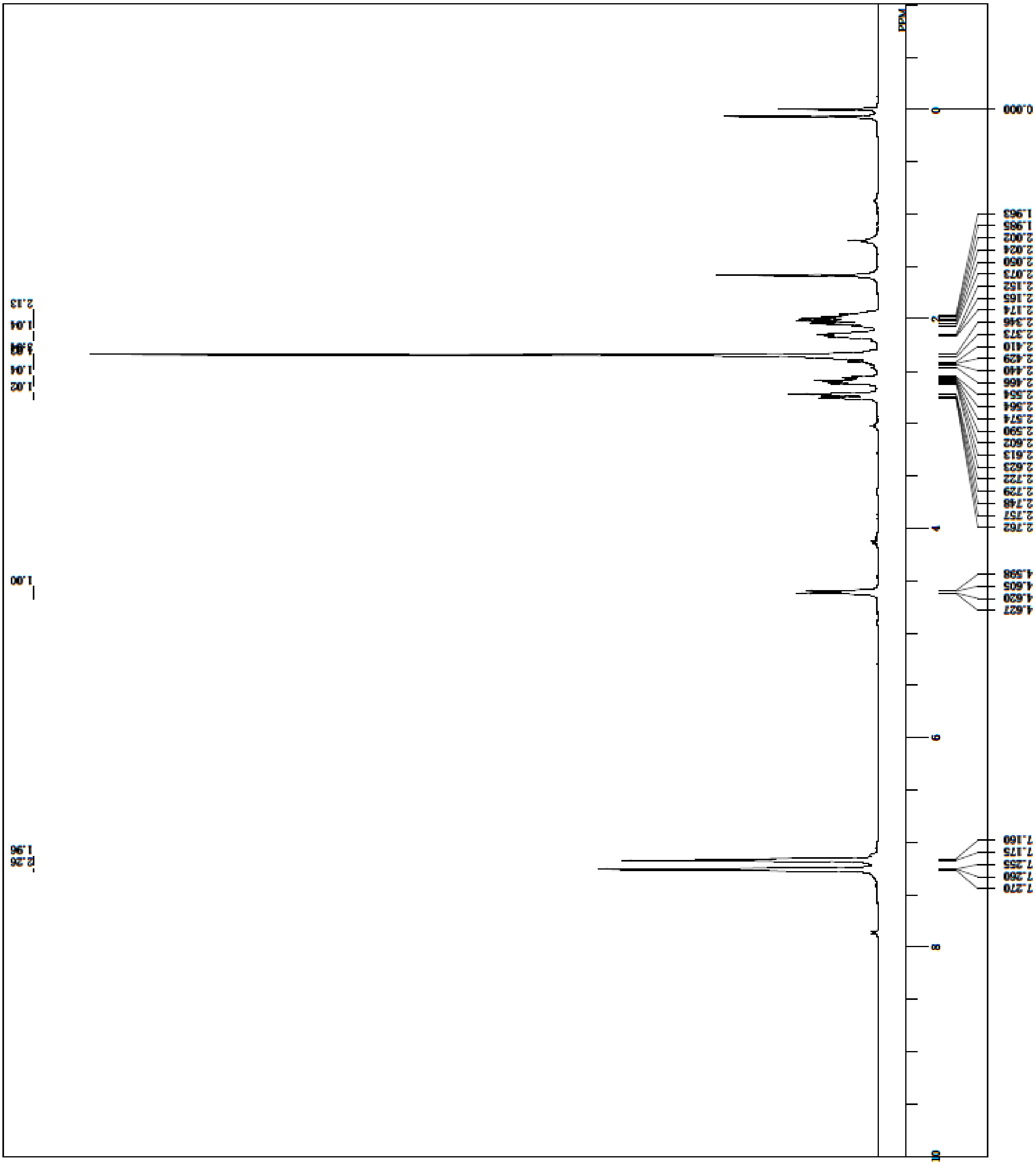

PPM
0
2
4
6
8
10
0.000
1.963
1.985
2.002
2.024
2.050
2.073
2.152
2.165
2.174
2.346
2.373
2.410
2.429
2.440
2.466
2.554
2.564
2.574
2.590
2.602
2.613
2.623
2.722
2.729
2.748
2.757
2.762
4.598
4.605
4.620
4.627
7.160
7.175
7.255
7.260
7.270
2.13
1.04
1.04
1.02
1.00
1.96
2.26

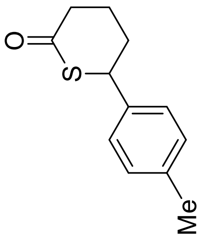
O
S
Me

2r

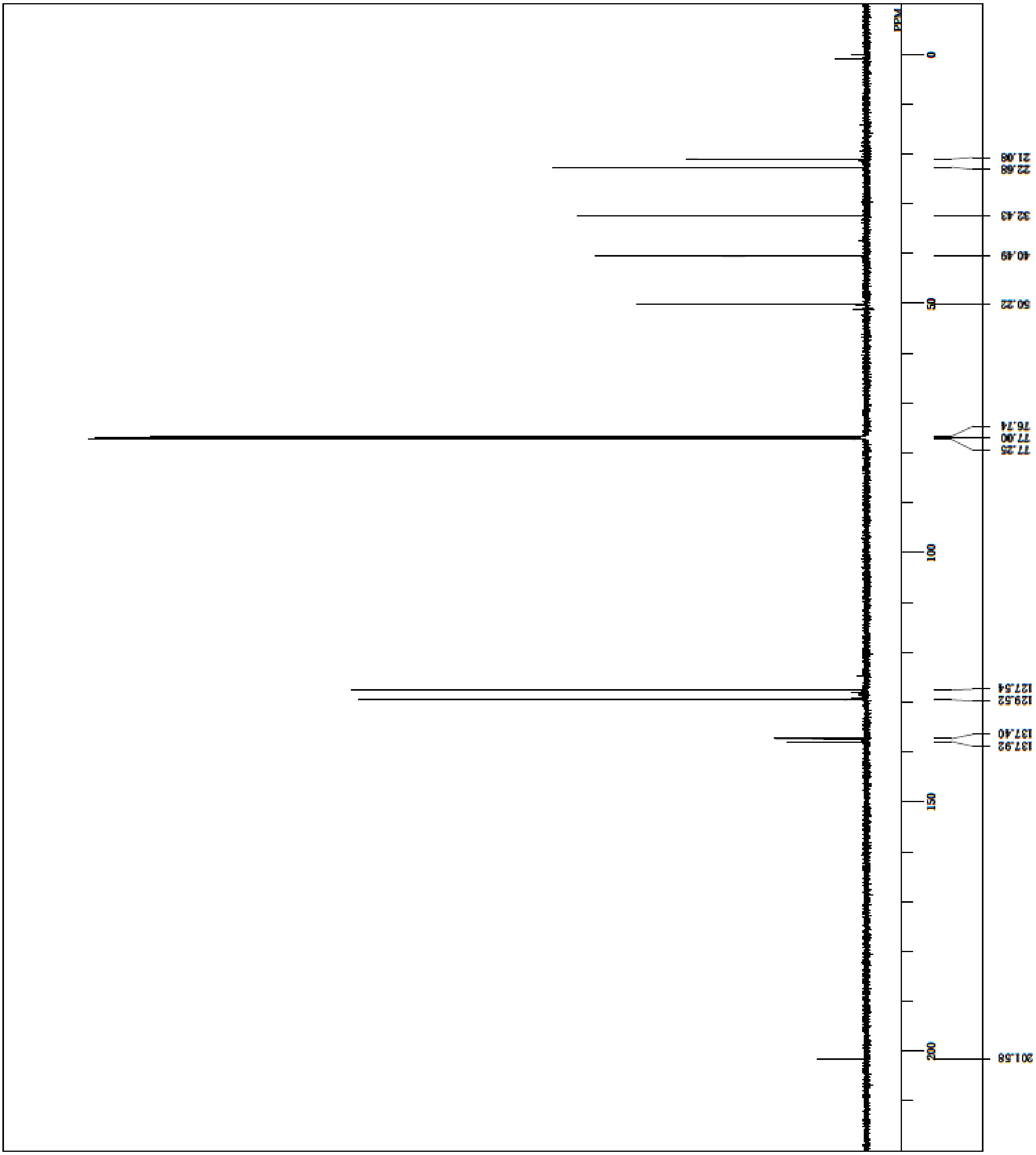
PPM
0
50
100
150
200
21.08
22.68
32.43
40.49
50.22
76.74
77.00
77.25
127.54
129.52
137.40
137.92
201.58

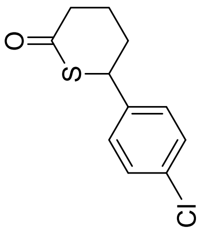
O
S
Cl

2s

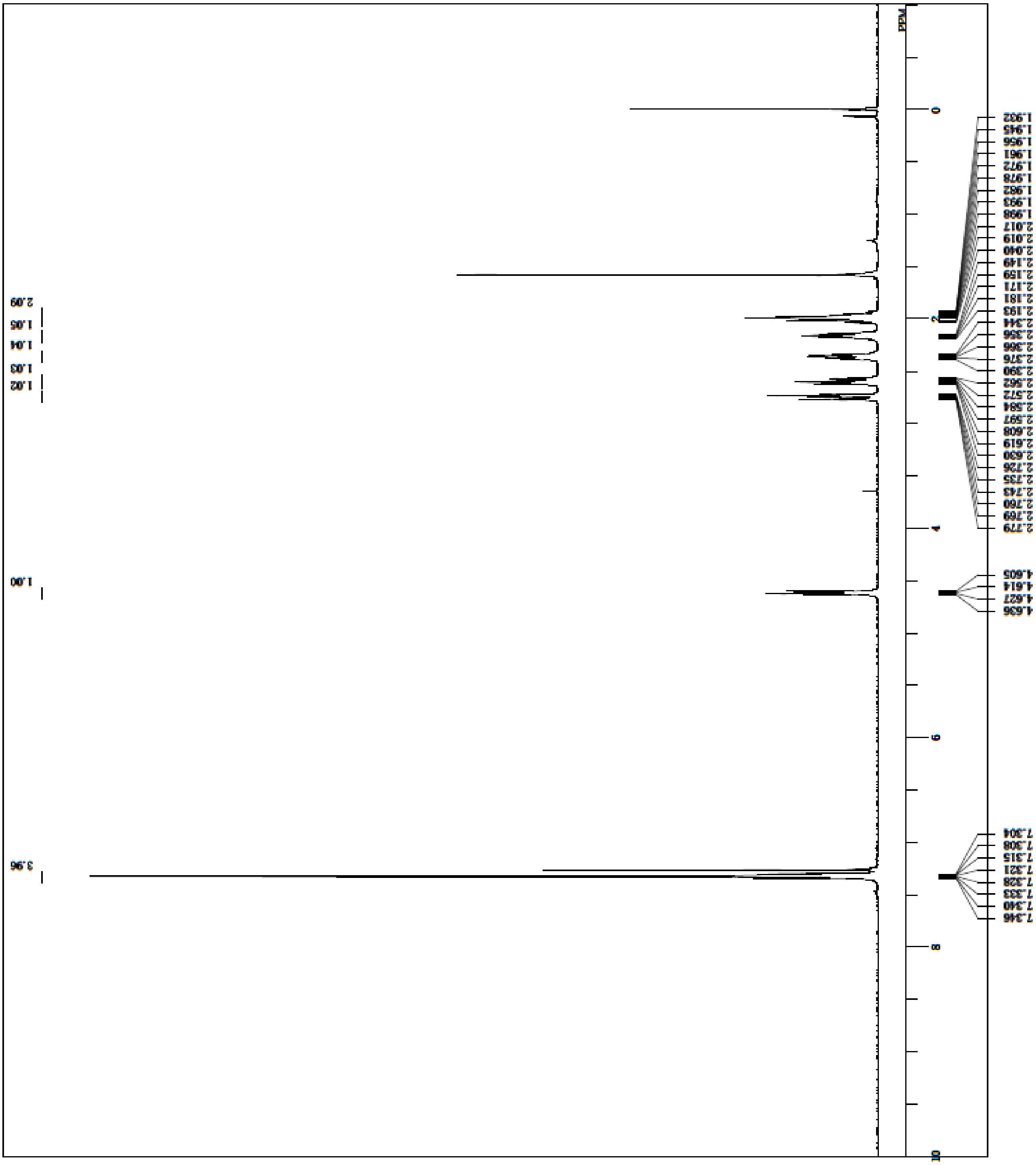
PPM
0
2
4
6
8
10
2.09
1.05
1.04
1.03
1.02
1.00
3.96
1.932
1.945
1.956
1.961
1.972
1.978
1.982
1.993
1.998
2.017
2.019
2.040
2.149
2.159
2.171
2.181
2.193
2.344
2.356
2.366
2.376
2.390
2.562
2.572
2.584
2.597
2.608
2.619
2.630
2.726
2.735
2.743
2.760
2.769
2.779
4.605
4.614
4.627
4.636
7.304
7.308
7.315
7.321
7.328
7.333
7.340
7.346

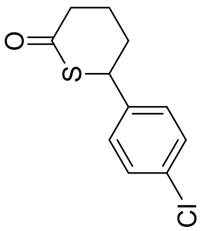
O
S
Cl

2s

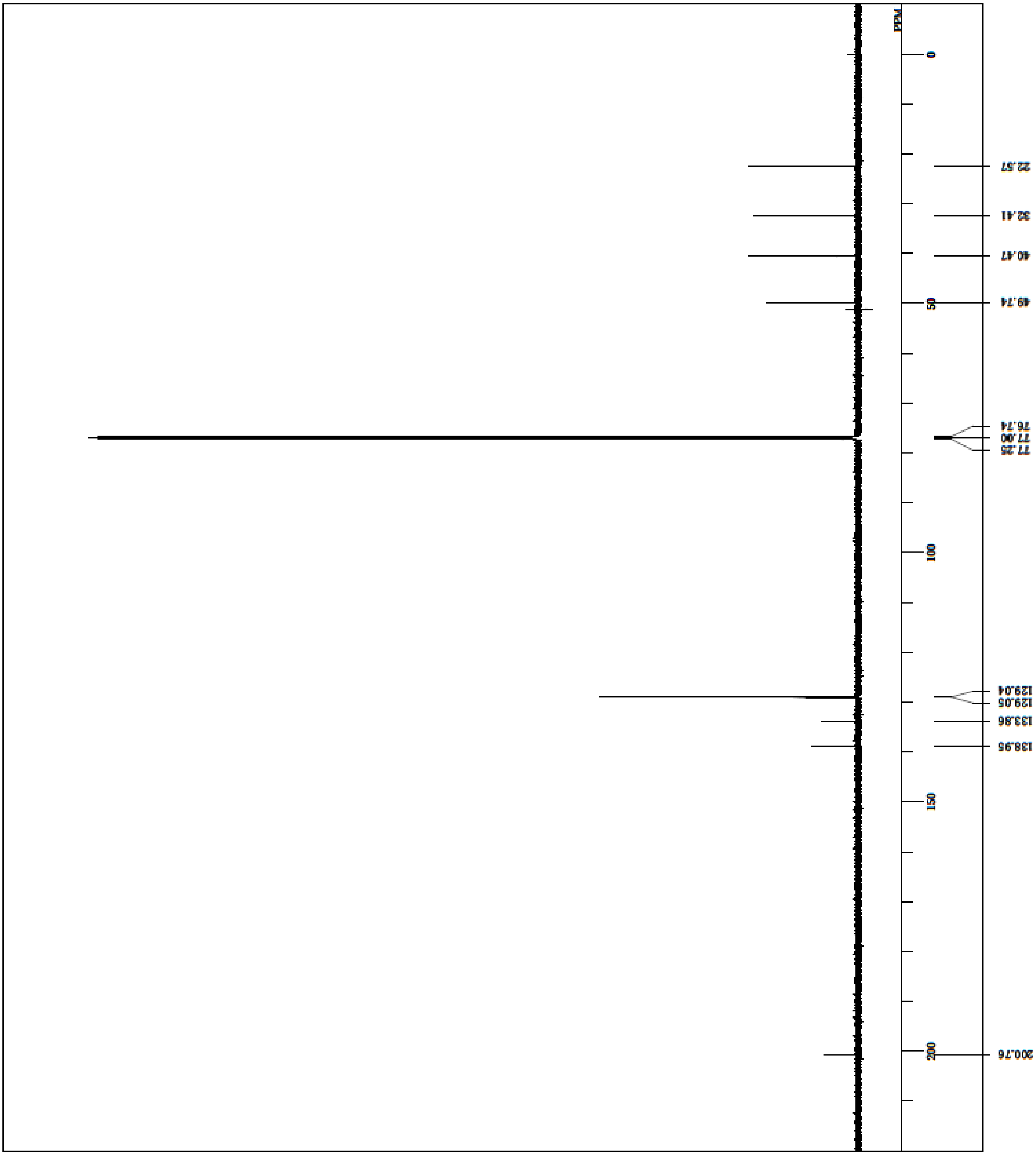
PPM
0
50
100
150
200
22.57
32.41
40.47
49.74
76.74
77.00
77.25
129.04
129.05
133.86
138.95
200.76

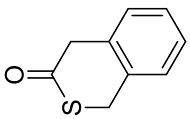

2t

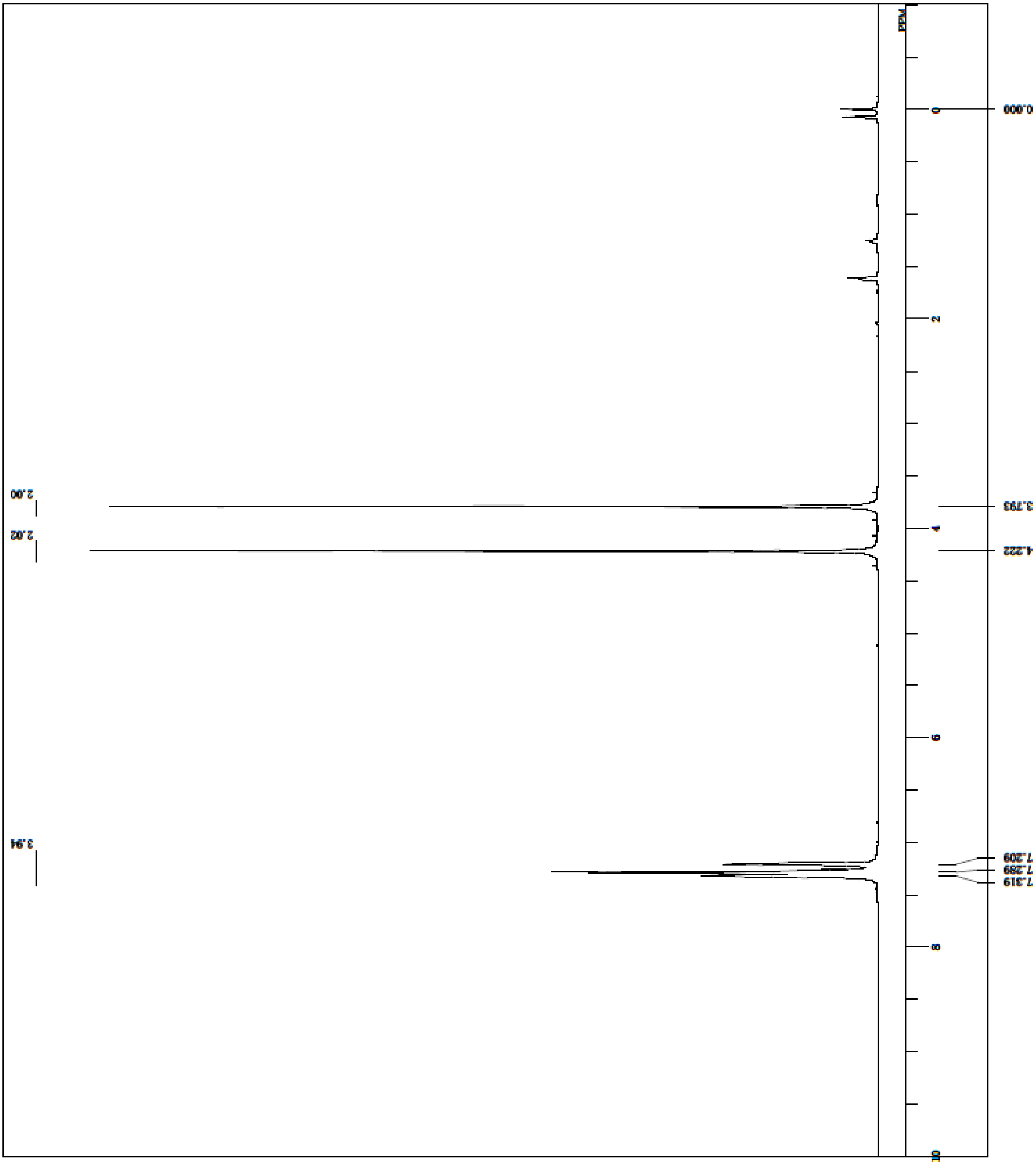

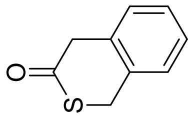

2t

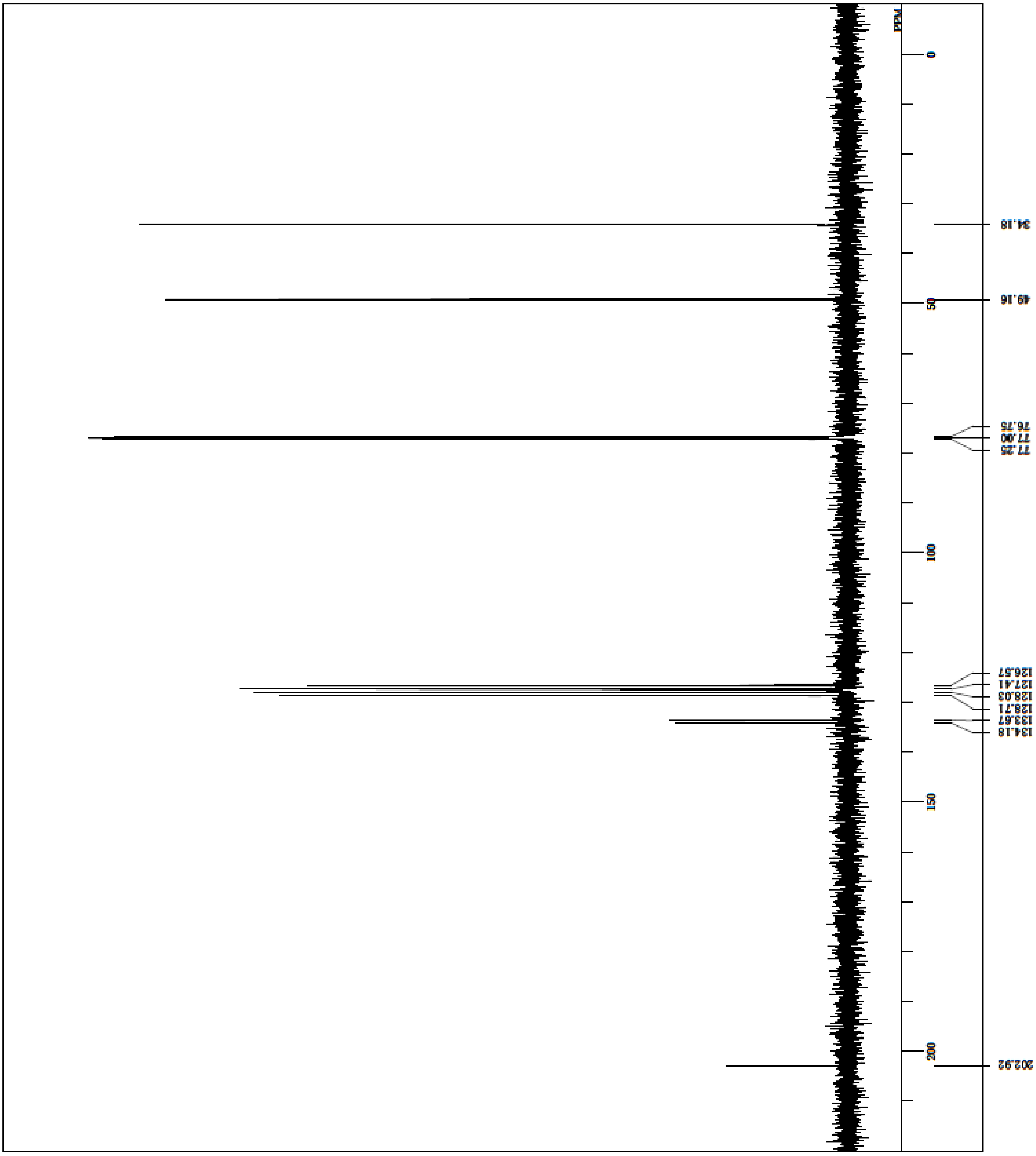

PPM
0
50
100
150
200
34.18
49.16
76.75
77.00
77.25
126.57
127.41
128.03
128.71
133.67
134.18
202.92

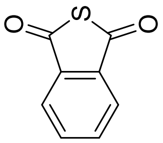

2u

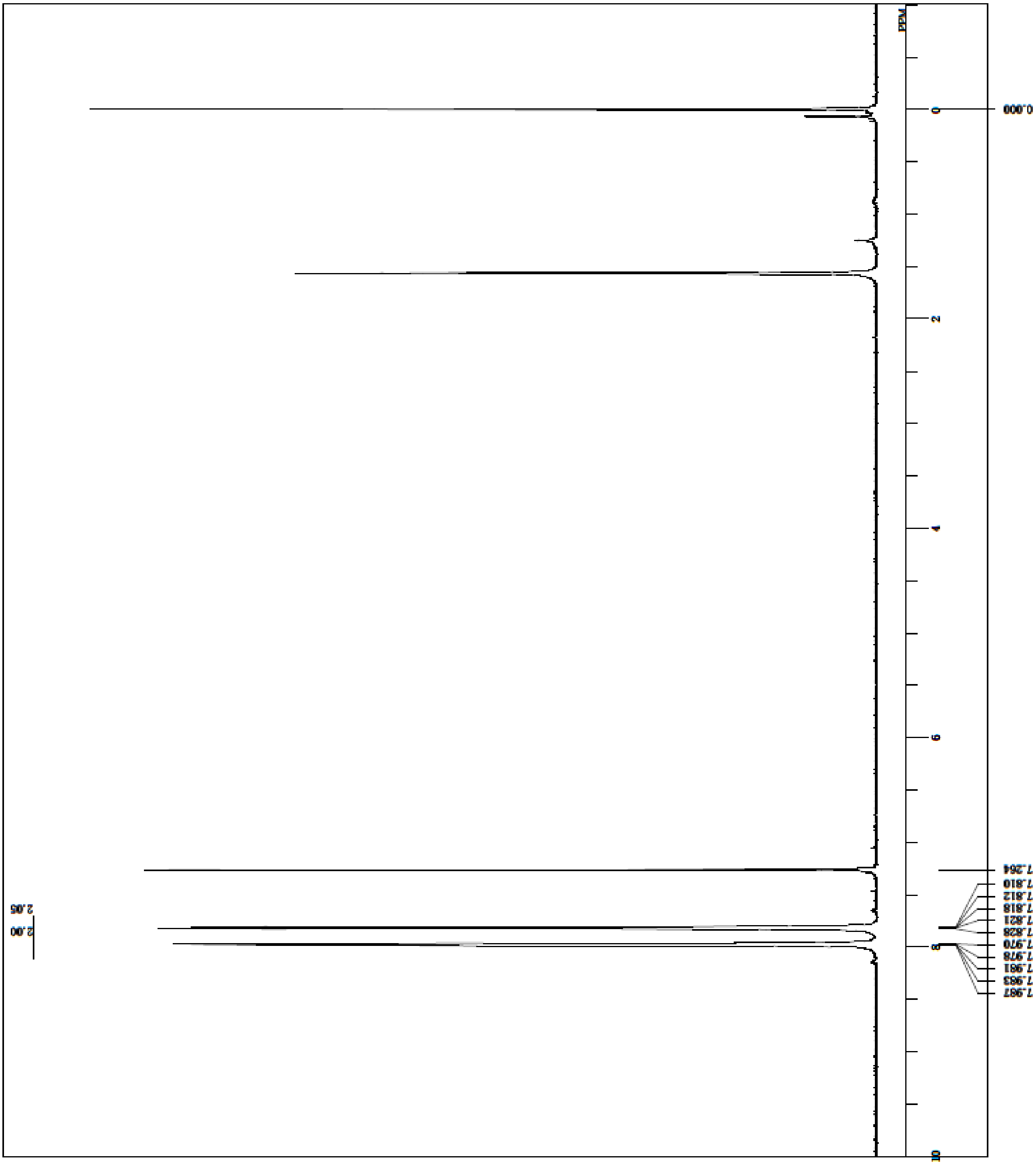

PPM
0
2
4
6
8
10
0.000
7.264
7.810
7.812
7.818
7.821
7.828
7.970
7.978
7.981
7.983
7.987
2.05
2.00

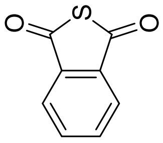

2u

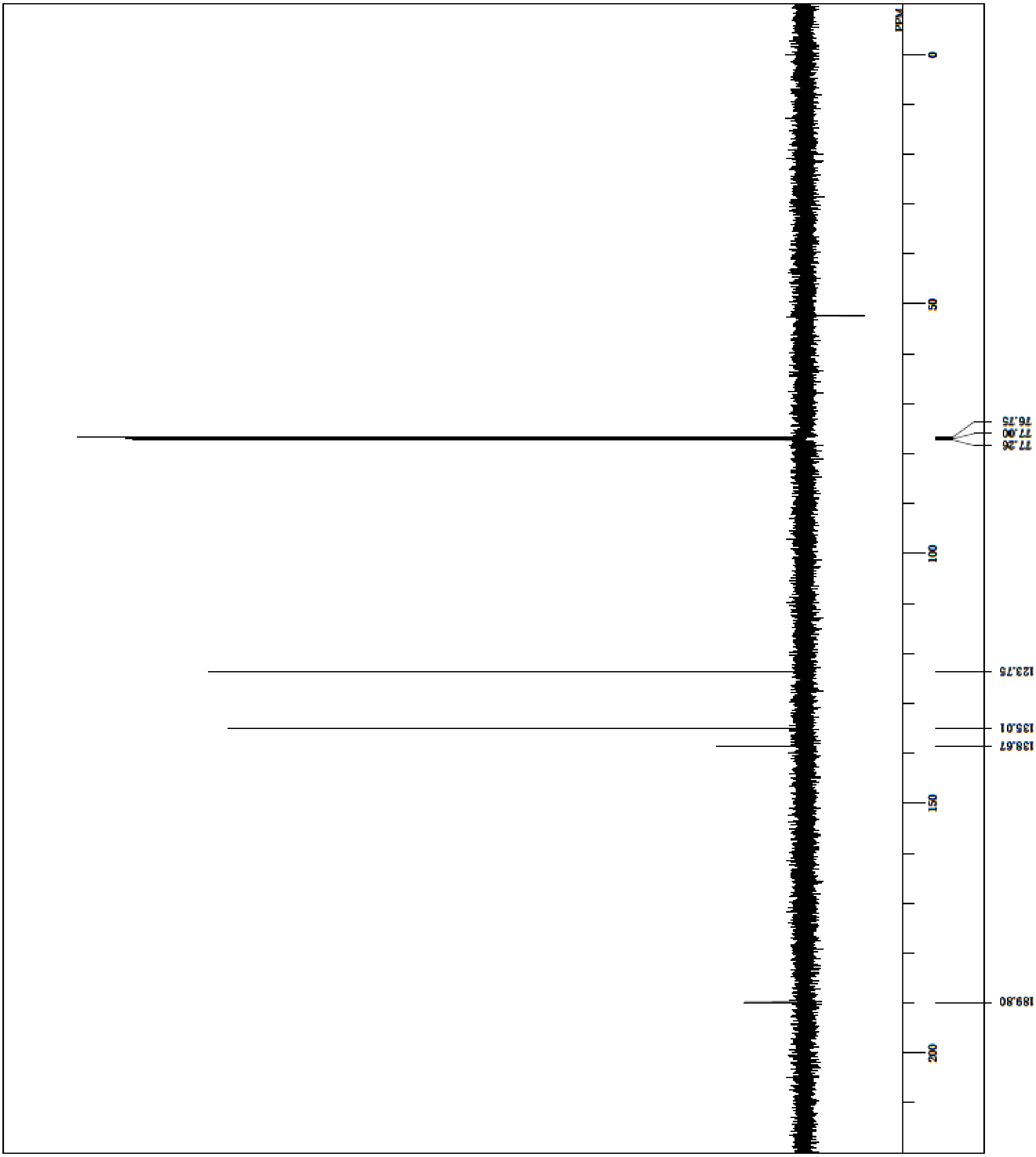

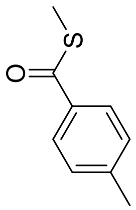

2v

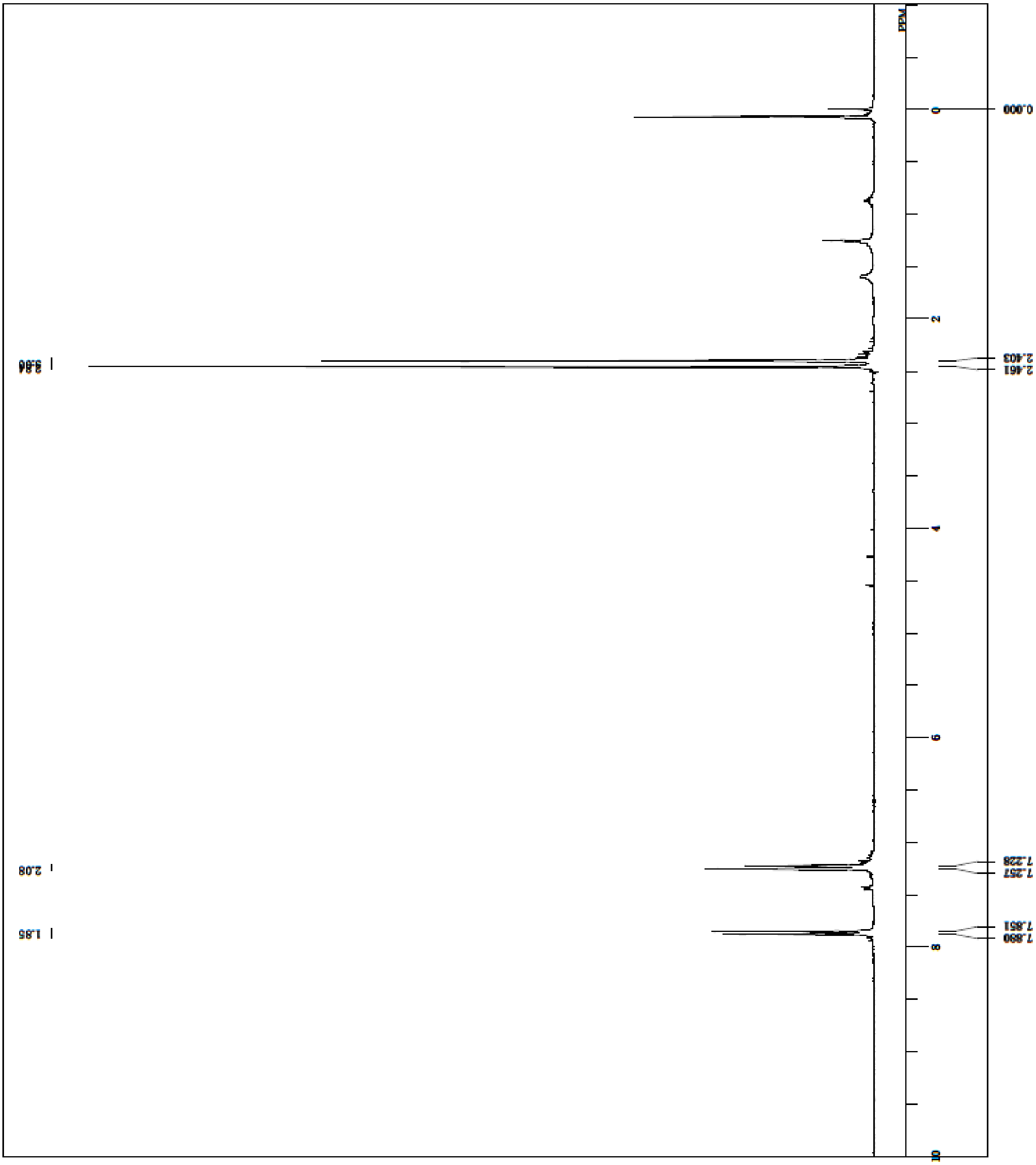

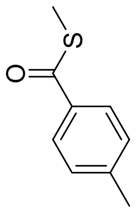
O
S

2v

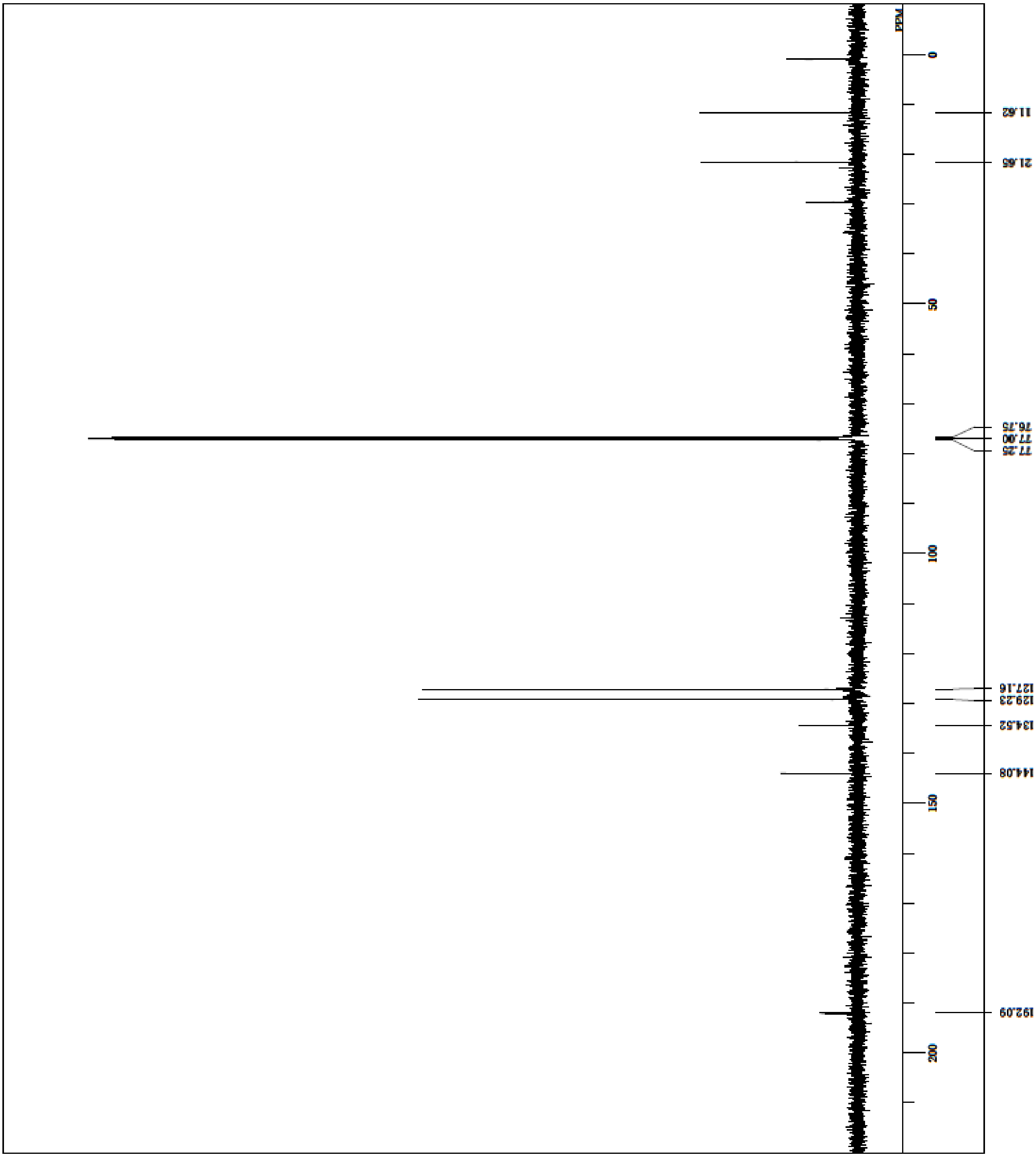
PPM
0
50
100
150
200
11.62
21.65
76.75
77.00
77.25
127.16
129.23
134.52
144.08
192.09

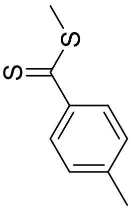
S
S

4v

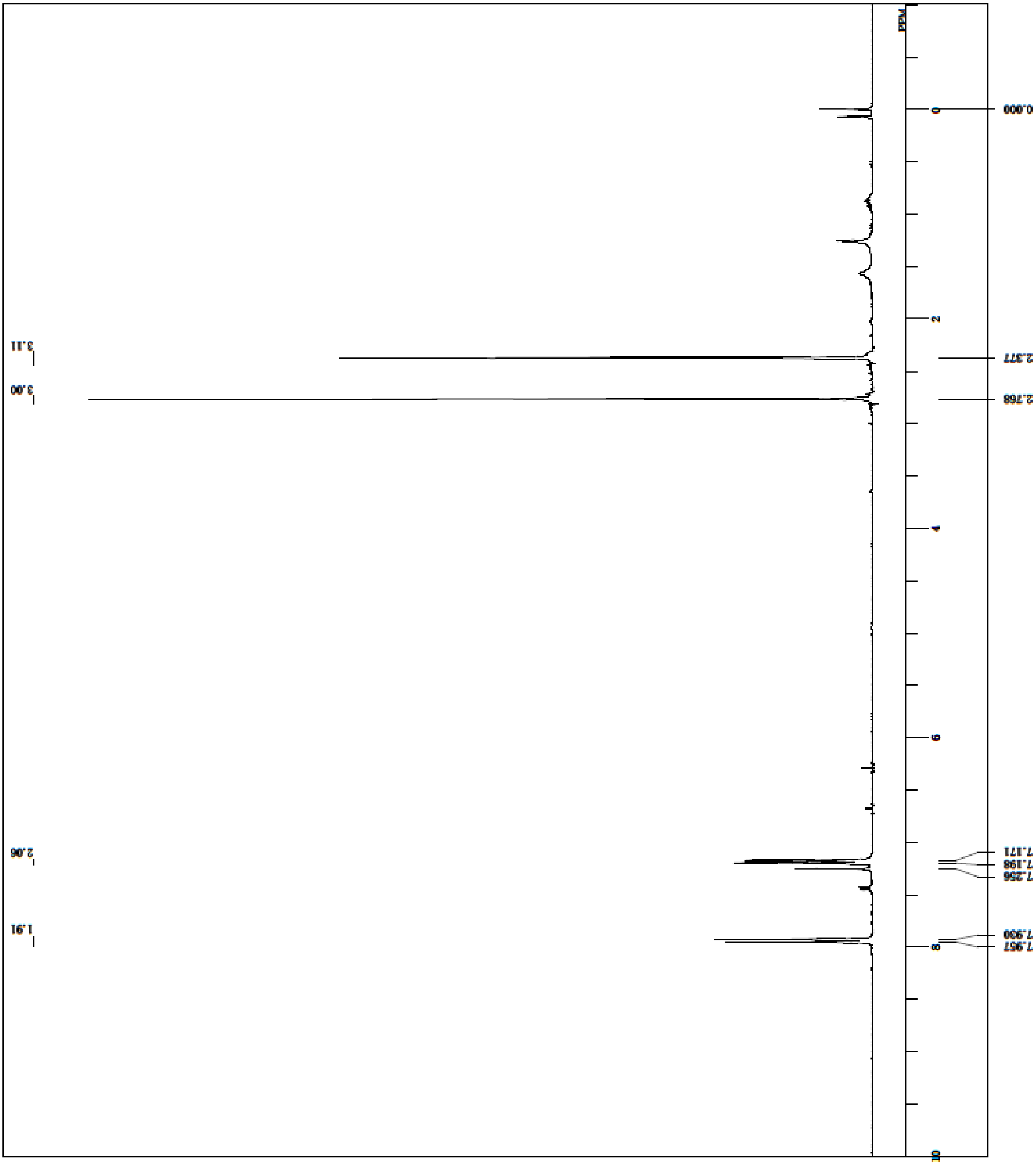
PPM
0
2
4
6
8
10
0.000
2.377
2.768
7.171
7.198
7.256
7.930
7.957
3.11
3.00
2.06
1.91

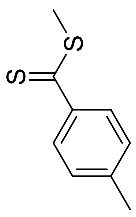
S
S

4v

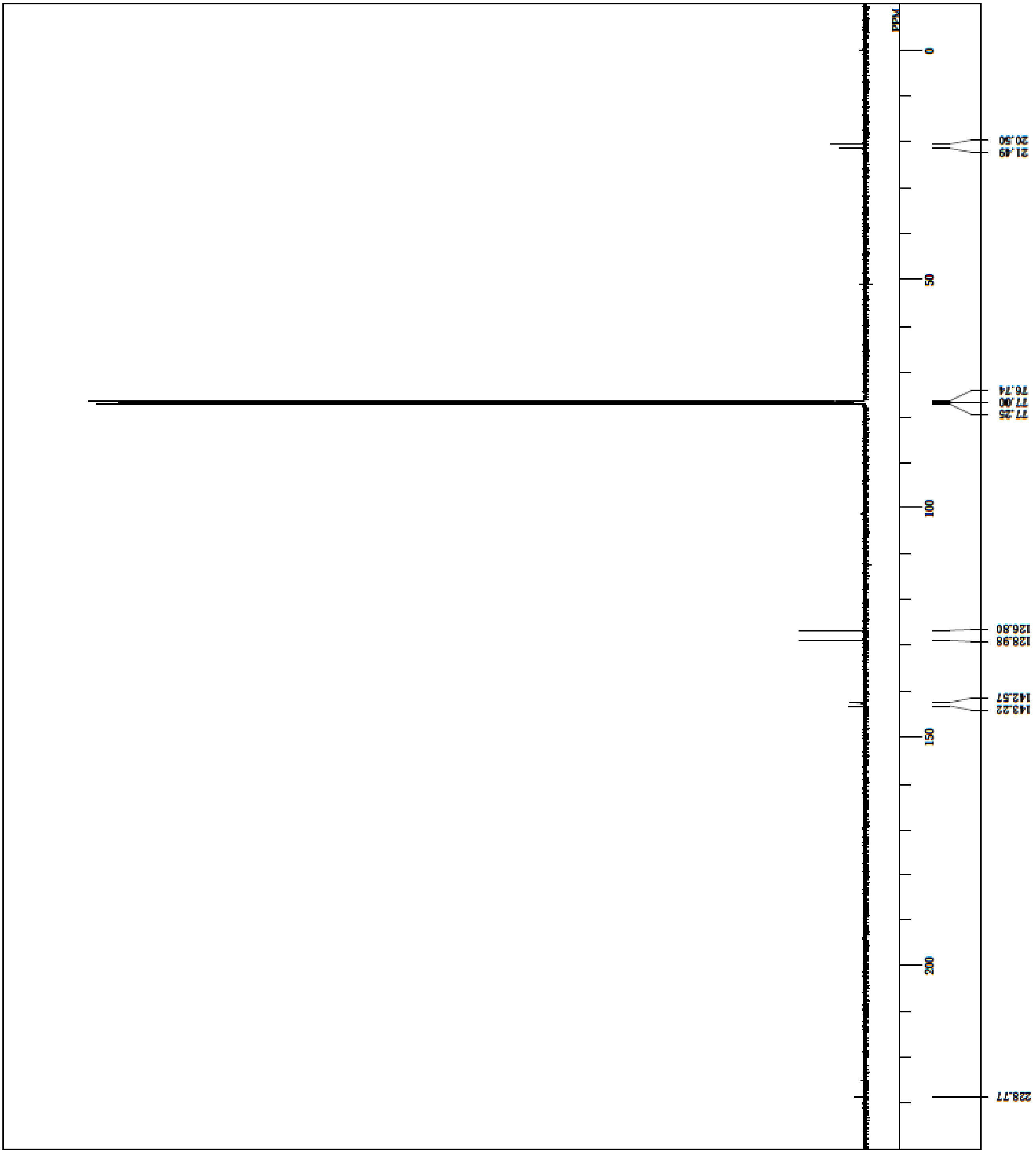
PPM
0
50
100
150
200
20.50
21.49
76.74
77.00
77.25
126.80
128.98
142.57
143.22
228.77

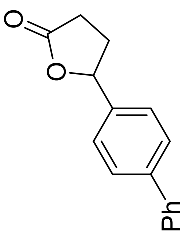

O
O
Ph

1f

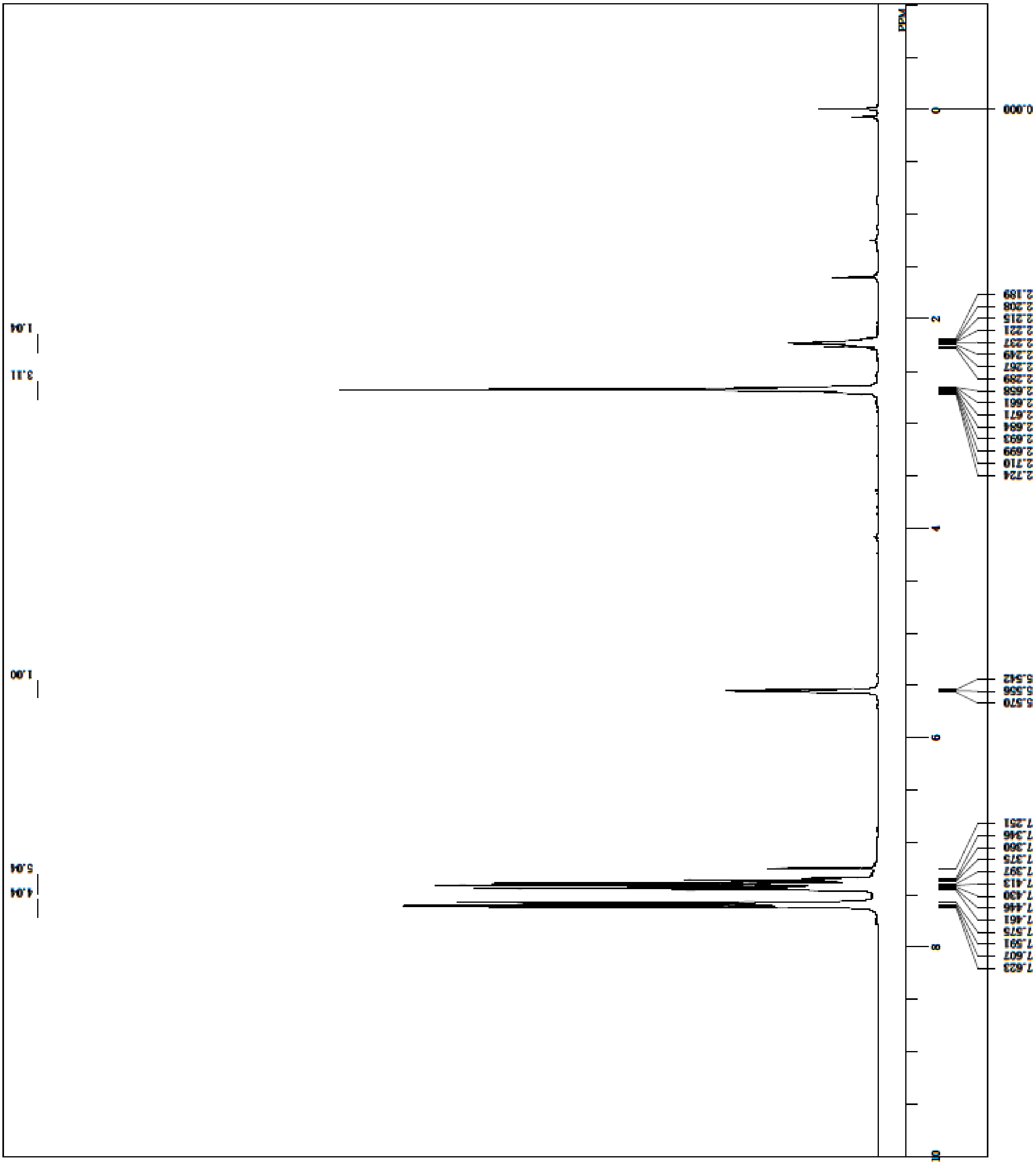

PPM
0.000
2.189
2.208
2.215
2.221
2.237
2.249
2.267
2.289
2.658
2.661
2.671
2.684
2.693
2.699
2.710
2.724
5.542
5.556
5.570
7.251
7.346
7.360
7.375
7.397
7.413
7.430
7.446
7.461
7.575
7.591
7.607
7.623
1.04
3.11
1.00
5.04
4.04

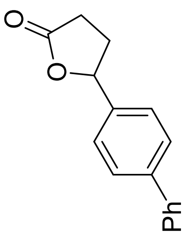
O
O
Ph

1f

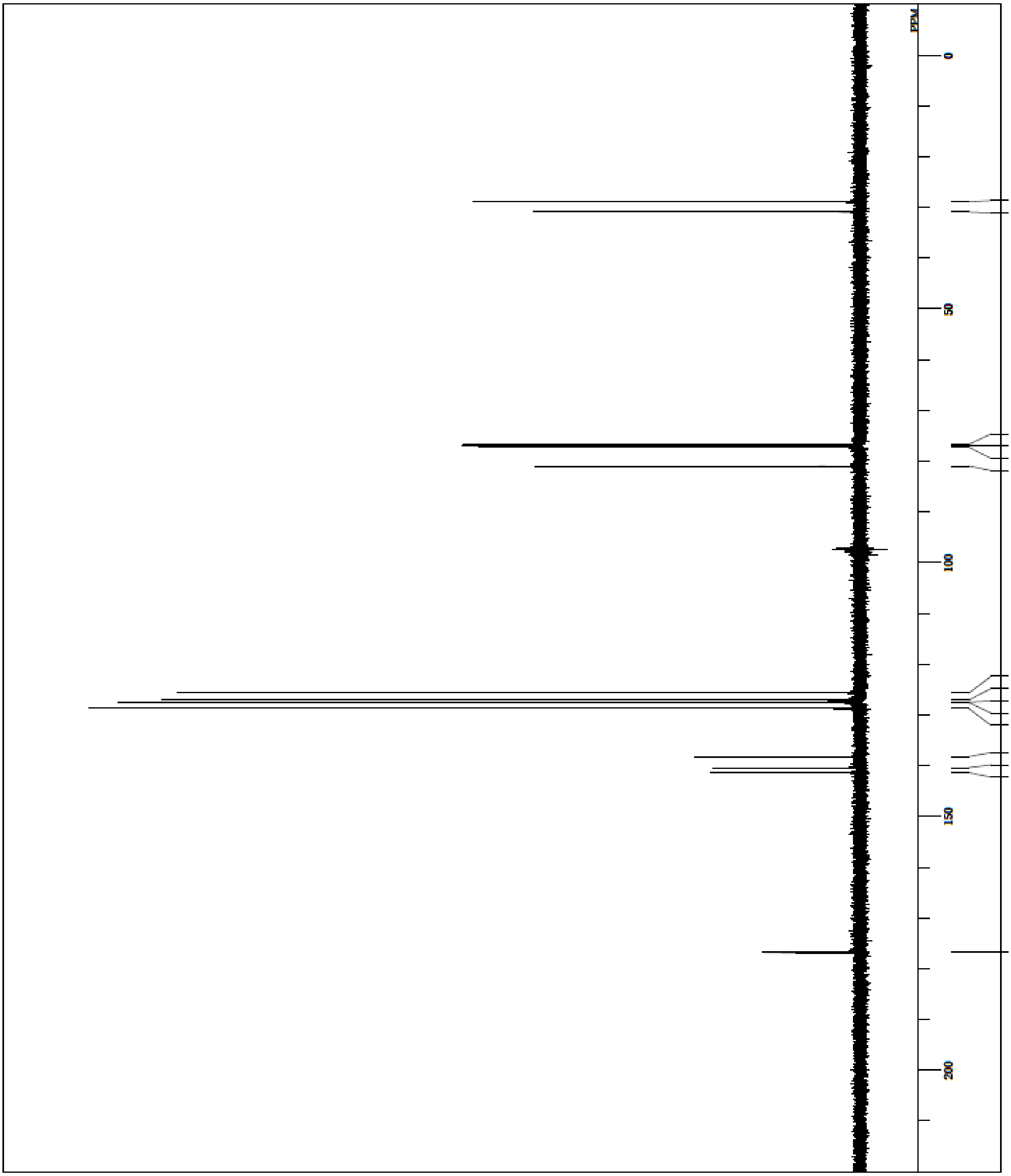
PPM
0
50
100
150
200
28.95
30.92
76.74
77.00
77.25
81.03
125.75
127.06
127.45
127.53
128.91
138.26
140.37
141.42
176.86

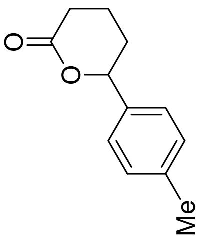

1r

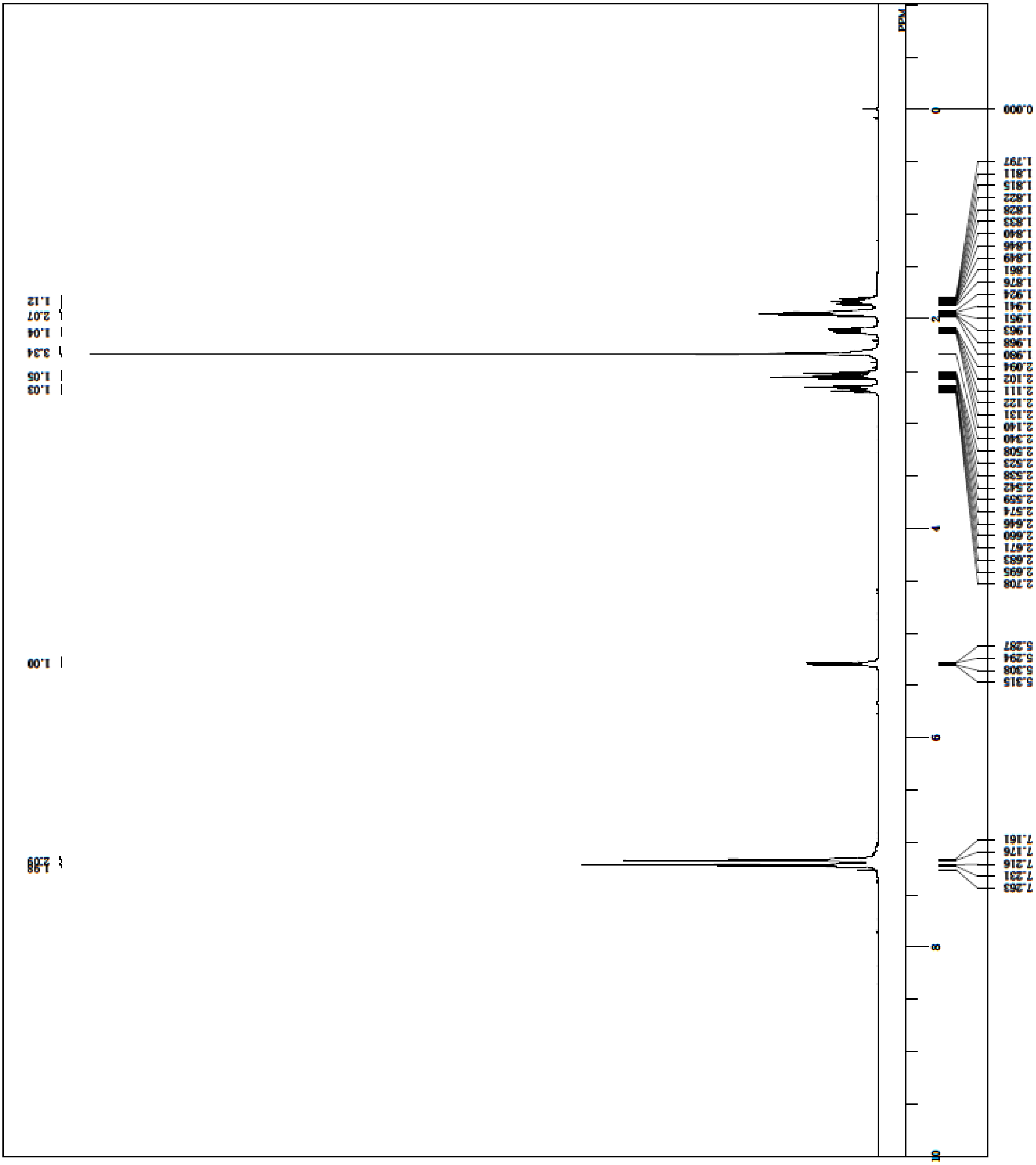

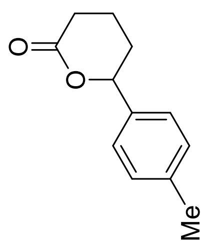

1r

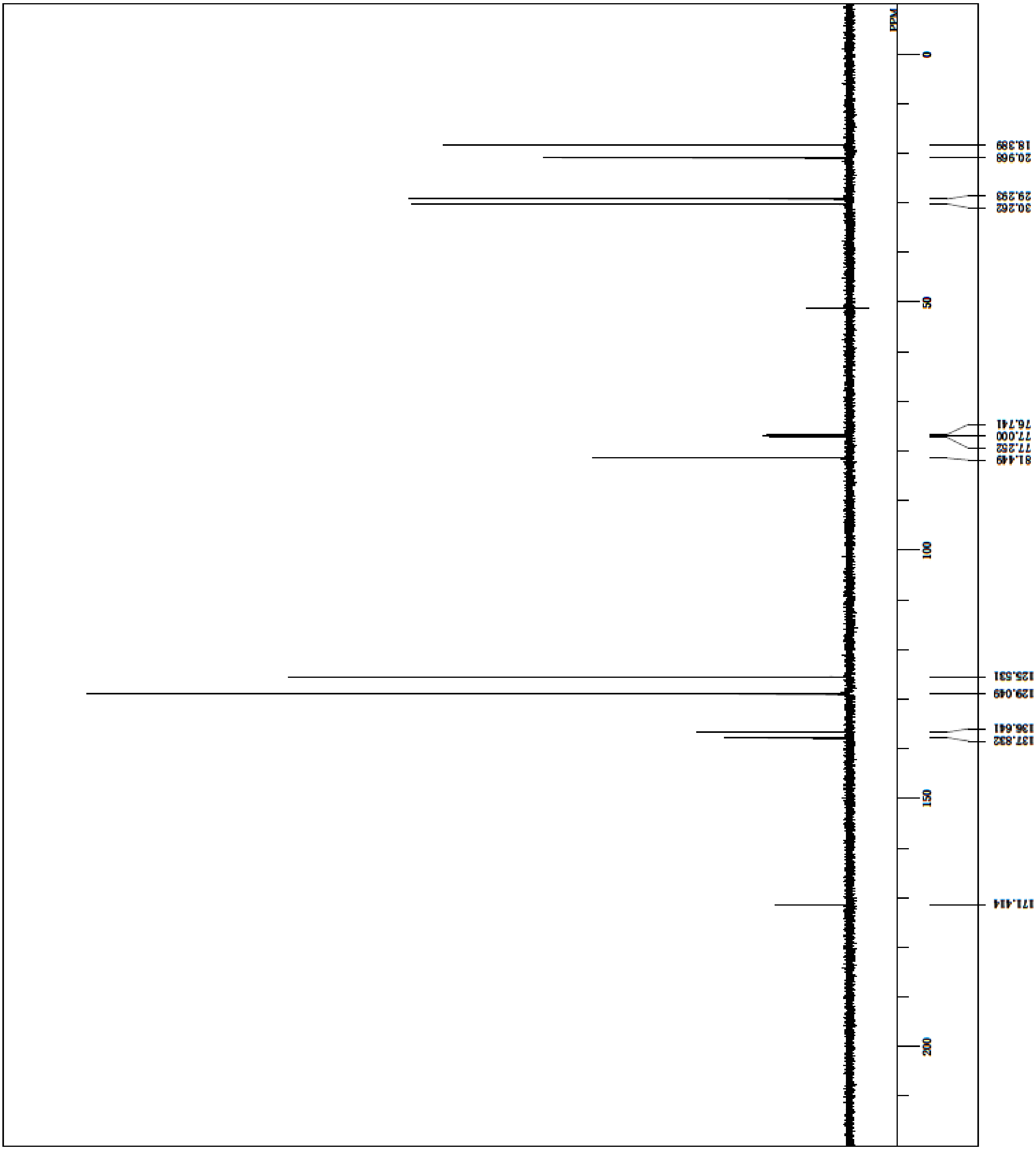
PPM
0
50
100
150
200
18.389
20.968
29.293
30.262
76.741
77.000
77.252
81.449
125.531
129.049
136.641
137.832
171.414

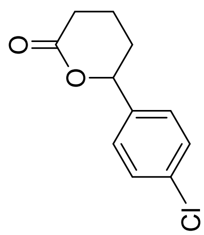
O
O
Cl

1s

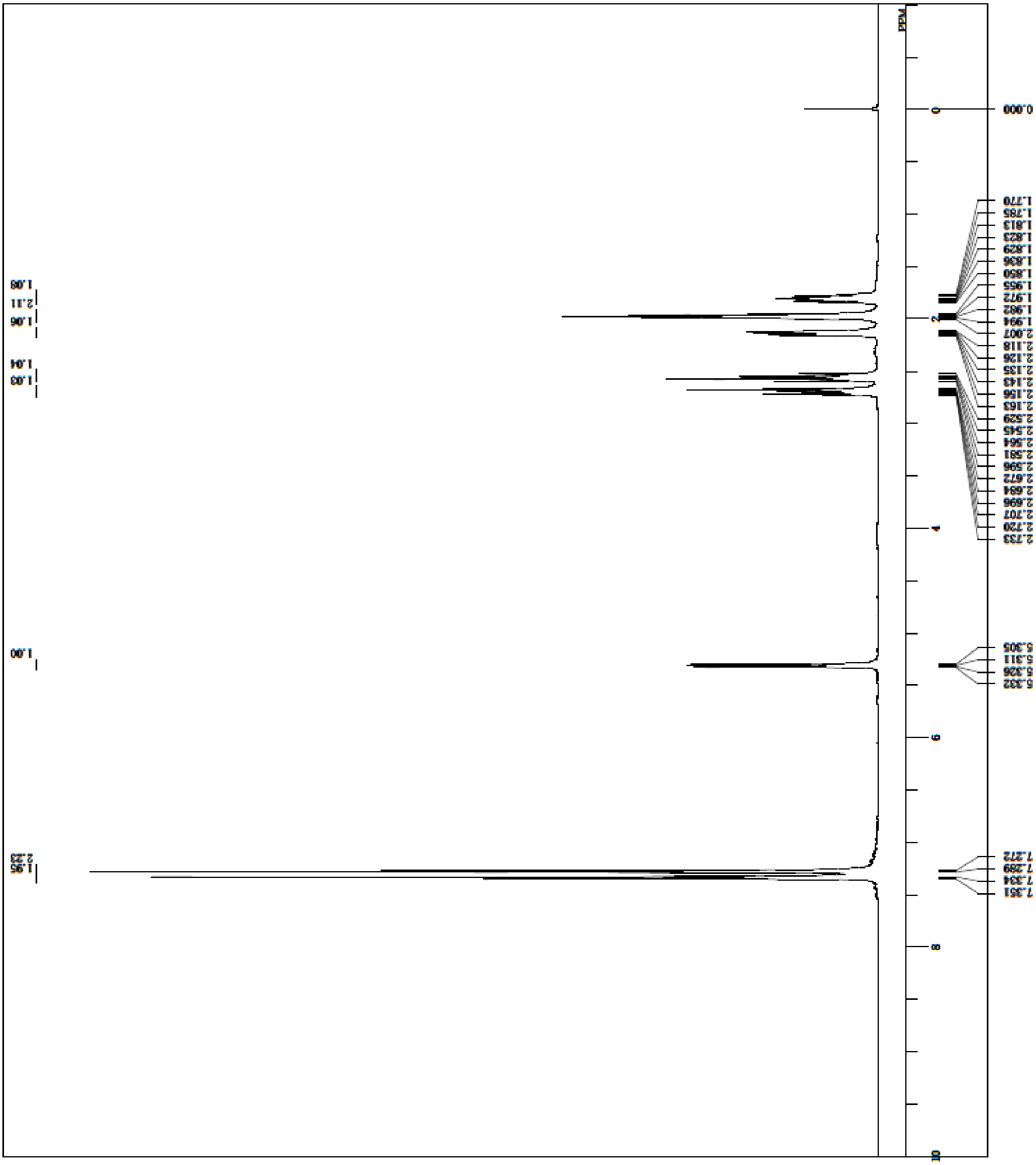
PPM
0.000
1.770
1.785
1.813
1.823
1.829
1.836
1.850
1.955
1.972
1.982
1.994
2.007
2.118
2.126
2.135
2.143
2.156
2.163
2.529
2.545
2.564
2.581
2.596
2.672
2.684
2.696
2.707
2.720
2.733
5.305
5.311
5.326
5.332
7.272
7.299
7.334
7.351

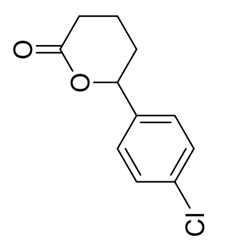
O
O
Cl

1s

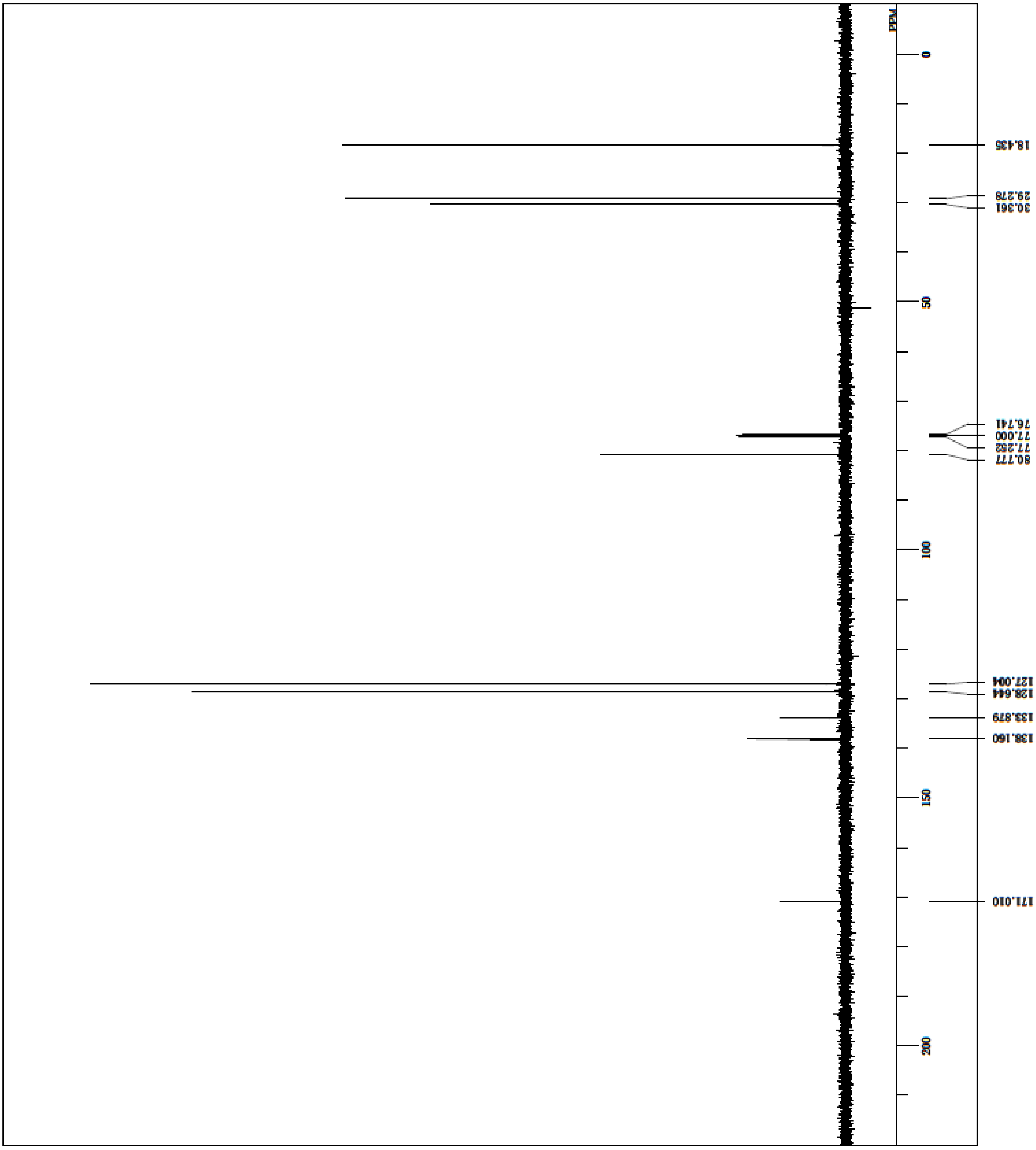
PPM
0
50
100
150
200
18.435
29.278
30.361
76.741
77.000
77.252
80.777
127.004
128.644
133.879
138.160
171.010
